# Biochemical and structural characterization of a novel arginine kinase from the spider *Polybetes pythagoricus*

**DOI:** 10.7717/peerj.3787

**Published:** 2017-09-11

**Authors:** Aldana Laino, Alonso A. Lopez-Zavala, Karina D. Garcia-Orozco, Jesus S. Carrasco-Miranda, Marianela Santana, Vivian Stojanoff, Rogerio R. Sotelo-Mundo, Carlos Fernando Garcia

**Affiliations:** 1 Instituto de Investigaciones Bioquímicas de La Plata “Dr. Prof. Rodolfo R. Brenner”, Universidad Nacional de La Plata, La Plata, Buenos Aires, Argentina; 2 Departamento de Ciencias Químico-Biológicas, Universidad de Sonora, Hermosillo, Sonora, Mexico; 3 Laboratorio de Estructura Biomolecular, Centro de Investigación en Alimentación y Desarrollo, A.C., Hermosillo, Sonora, Mexico; 4 Photon Science Directorate, National Synchrotron Light Source II, Brookhaven National Laboratory, Upton, NY, United States of America

**Keywords:** Arginine kinase, Spider, cDNA cloning, Crystal structure, Phosphagen, *Polybetes pytagoricus*, Arthropoda, Arachnida, Open conformation, Allergen

## Abstract

Energy buffering systems are key for homeostasis during variations in energy supply. Spiders are the most important predators for insects and therefore key in terrestrial ecosystems. From biomedical interest, spiders are important for their venoms and as a source of potent allergens, such as arginine kinase (AK, EC 2.7.3.3). AK is an enzyme crucial for energy metabolism, keeping the pool of phosphagens in invertebrates, and also an allergen for humans. In this work, we studied AK from the Argentininan spider *Polybetes pythagoricus* (*Pp*AK), from its complementary DNA to the crystal structure. The *Pp*AK cDNA from muscle was cloned, and it is comprised of 1068 nucleotides that encode a 384-amino acids protein, similar to other invertebrate AKs. The apparent Michaelis-Menten kinetic constant (*K_m_*) was 1.7 mM with a *k*_cat_ of 75 s^−1^. Two crystal structures are presented, the apo*Pv*AK and *Pp*AK bound to arginine, both in the *open* conformation with the active site lid (residues 310–320) completely disordered. The guanidino group binding site in the apo structure appears to be organized to accept the arginine substrate. Finally, these results contribute to knowledge of mechanistic details of the function of arginine kinase.

## Introduction

Arginine kinase (AK) (ATP: L-arginine phosphotransferase. EC 2. 7. 3. 3) is an enzyme which catalyzes the reversible transfer of the γ-phosphoryl group (PO${}_{4}^{-}$) of ATP to L-arginine (Arjunwadkar & Raghupathi Rami Reddy, 1985; Blethen, 1970) and is widely distributed in invertebrates. AK is found mainly in muscle and heart tissue, although it is also expressed in gills, intestine, digestive glands (Blethen & Kaplan, 1968) and eggs (Ratto & Christen, 1988). Phosphoarginine is accumulated in tissues when ATP is present, and ADP is back-phosphorylated by AK.

AK has been described in insects such as beetles (Tanaka et al., 2007), lepidopterans (Binder et al., 2001; Chamberlin, 1997), hymenopterans (Wang et al., 2009a), cockroaches (Brown & Grossman, 2004) and locusts (Wu et al., 2007). It is present in crustaceans (García-Orozco et al., 2007), mollusks and echinoderms among other invertebrates (Morrison, 1973). Considering that the sub-phylum Chelicerate is comprised of more than 75,000 species, only the AK from horseshoe crab (Strong & Ellington, 1995), scorpion (Arjunwadkar & Raghupathi Rami Reddy, 1985) and spider (Blethen & Kaplan, 1968; Bobolea et al., 2011) have been studied.

In spiders, fast movements are limited by depletion of phosphagens, accumulation of lactate and limited aerobic metabolism (Prestwich, 1988), suggesting that AK is key for this process. AK has a similar function to creatine kinase (CK) from vertebrates (ATP: N-creatine phosphotransferase, EC 2.7.3.2,) providing fast energy during muscular contraction by the back-phosphorylation of ADP by phospho-arginine (Bessman & Carpenter, 1985). In the fire ant (*Solenopsis invicta*), AK is highly expressed in the tissues (thorax and head) and castes (workers) with a larger energetic demand, confirming the connection between the biochemical function and the physiology (Wang et al., 2009a).

Besides the role of AK in energy maintenance, it is also important as an allergen. Crustacean AK is a well-characterized crustacean allergen (García-Orozco et al., 2007; Yu & Li, 2003) and also described in insects (Binder et al., 2001; Sookrung et al., 2006) and a spider (Bobolea et al., 2011). Recently it has been shown that there is cross-reactivity between crustacean and insect AKs, possibly due to structural epitopes (Srinroch et al., 2015). On the other hand, because the enzyme plays a significant role in energy metabolism and it is absent in vertebrates, AK was proposed as a target for insecticides (Wang et al., 2009a; Wu et al., 2009) like the insect *Ctenocephalides sp.* (Werr, Cramer & Ilg, 2009). To contribute to the biochemical and structural knowledge of phosphagen kinases, we studied the AK from *Polybetes pytagoricus* (*Pp*AK). This represents the first structural study of a chelicerate phosphagen kinase and allergen.

## Materials & Methods

### RNA isolation and cDNA cloning of arginine kinase

Adult spider *P. pythagoricus* were collected from eucalyptus trees in La Plata City, (Province of Buenos Aires, Argentina; 34°52′56″S, 57°56′07″W). Spiders were anesthetized by cooling, and leg muscle was dissected. Total RNA was isolated from 100 mg of tissue using Trizol reagent (Thermo Fisher Scientific, Waltham, MA, USA) following the manufacturer’s instruction. The RNA concentration was measured using a nano spectrophotometer (Nanodrop ND-1000) and the integrity was determined by agarose gel electrophoresis. A double stranded cDNA library was constructed following the instructions of Smart cDNA library Construction Kit (Clontech Laboratories Inc. Palo Alto, CA, USA). The synthesized cDNAs were electrophoresed and excised from the agarose gel separating three groups according to the size (0.2–1 kb, >1–3 kb, >3 kb) to be ligated in the TA cloning vector TOPO XL PCR (Invitrogen, Carlsbad, CA, USA). The ligation and transformation were carried out according to the manufacturer’ manual. White colonies containing inserts bigger than 300 bp were detected by PCR and selected to be cultured overnight in 2 ml of LB broth with ampicillin. From these, 384 colonies were selected, plasmid was obtained using an alkaline lysis protocol (Ausubel et al., 1995), and sent for Sanger DNA sequencing to the University of Arizona Genetics Core Lab (Tucson AZ, USA). ESTs were assembled into contiguous sequences (contigs) using the CLC Main Workbench program (CLC Bio, a QIAGEN Company; Aarhus, Denmark). All contigs were searched against public databases in GenBank and Swiss-Prot by BLASTX and BLASTN and one 632 bp contigs with high identity to invertebrate AK was obtained. The Rapid Amplification of cDNA Ends (RACE) kit (Thermo Fisher Scientific, Waltham, MA, USA) was used to complete the partial sequence of cDNA that codify for arginine kinase (*Pp*AK). The *Pp*AK specific primers used for RACE were AKAF1 5’ACCATGGTTGACCAAGCTAC, AKAF2 5’ GACAAACATCCTCCAACCGAC and AKAF2nes 5’CACCATTGTCAACGTCGATCC. The nucleotide and deduced amino acid sequence were deposited at GenBank with the accession number MF001441.

### Purification of recombinant *Pp*AK protein

A synthetic gene optimized for *E. coli* overexpression including a His-tag at the N-terminus was synthesized using the *P. pythagoricus* AK amino acid sequence by DNA 2.0. The recombinant construct was transformed into the *E. coli* BL21(DE3) Rosetta strain. Cultures were grown at 37 °C by inoculating 1 ml of an overnight culture into 100 ml of LB medium containing 50 mg/ml ampicillin. Incubation in an orbital shaker at 200 rpm and 37 °C was continued until the culture reached an A_600_ of 0.6. and isopropyl thiogalactopyranoside (IPTG) was added to a final concentration of 0.1 mM. After 4 h of induction at 28 °C, bacteria were harvested by centrifugation at 4,000× g for 5 min. The pellet was resuspended in cold lysis buffer (50 mM Tris, 0.5 mM PMSF, 500 mM NaCl, 5 mM EDTA, 1 mM DTT), sonicated on ice (10 W, 10 s, 10 times with 10 s interval). The supernatant was obtained by centrifuging the lysate at 10,000× g for 20 min at 4 °C.

The His-tagged recombinant protein was purified using immobilized metal ion affinity chromatography (IMAC) (IMAC Sepharose 6 Fast Flow; GE Healthcare, Little Chalfont, UK). The resin was equilibrated with buffer A (0.5 M NaCl, 20 mM Tris-HCl pH 7.9). The sample was loaded onto the IMAC column. The column was washed with 5 column volumes of buffer A and the protein was eluted with 5 volumes of buffer B (0.5 M NaCl, 20 mM Tris-HCl pH 7.9, 500 mM imidazole (Sigma-Aldrich, St. Louis, MO, USA)) at a flow rate of 0.5 ml/min. Fractions of 1 ml were collected during the elution and monitored at 280 nm to detect the presence of protein. The fraction containing protein was isolated and dialyzed against 100 mM glycine and 10 mM beta-mercapto-ethanol. The purity of the AK was determined by 12% SDS-PAGE using stain-free gels (Bio-Rad Laboratories, Hercules, CA, USA), and the protein concentration was estimated by the bicinchoninic acid method (Pierce Biotechnology, Waltham, MA, USA) using bovine serum albumin as standard.

### AK activity

AK Activity was measured by determining the rate of formation of ADP according to the protocol described by Blethen (1970) with modifications (García-Orozco et al., 2007). In this system three coupled reactions are used: (1) the AK enzyme phosphorylates L-arginine from ATP to form ADP, (2) ADP is again phosphorylated by the action of pyruvate kinase forming pyruvate, (3) the latter is reduced by oxidizing NADH lactate dehydrogenase. The rate of NADH oxidation was measured spectrophotometrically at 340 nm. The reaction was done at 30 °C in a quartz cuvette containing 1 ml of buffer containing 178 mM glycine, 0.33 mM 2-mercaptoethanol, 133 mM potassium chloride, 13 mM magnesium sulfate, 20 mM phospho (enol) pyruvate, 6.7 mM ATP, 0.13 mM nicotinamide adenine dinucleotide (NADH), 2 U of pyruvate kinase, 3 U of lactic dehydrogenase, 17 mM L-arginine and 30 μg of *Pv*AK at pH 8.6. This amount of enzyme is equivalent to 0.75 μmol using the amino acid deduced molecular mass or a final concentration of 0.76 mM in the reaction cuvette.

Control reactions were performed with all reagents except the AK enzyme and were monitored by absorbance at 340 nm. Absorbance data was collected for 5 min. All data processing was done in GraphPad Prism version 6.0 using the non-linear regression kinetic model to calculate the Michaelis–Menten constants. One unit or arginine kinase is defined as the amount of enzyme that generates 1.0 μmol of phospho-L-arginine per minute using ATP and L-arginine as substrates, at pH 8.6 and 30 °C (Blethen & Kaplan, 1968).

### Crystallization of apo form and arginine complex of *Pp*AK

Purified *Pp*AK was exhaustively dialyzed against buffer Tris-HCl 20 mM pH 7.9 to eliminate salts and imidazole and concentrated to 24 mg/ml using a 10-kDa membrane cartridge (Amicon; Millipore, Billerica, MA, USA). Crystallization experiments were done by the vapor-diffusion method using the hanging-drop technique in 24-well Limbro plates. Hampton Research Crystal Screen I kit was used to set up each drop by adding 0.4 mL of each precipitating solution to the well and mixing in the siliconized coverglass 2 μl *Pp*AK and 2 μl of each crystallization solution. Each well was carefully sealed with vacuum grease and stored at 16 °C. Plates were monitored each week with a stereoscope and under polarized light. Apo-*Pp*AK crystal grew after two weeks as a bunch of large square and clear plates (0.25 × 0.15 × 0.05 mm) in solution # 9 (0.2 M ammonium acetate, 0.1 M sodium citrate tribasic dihydrate pH 5.6 and 30% w/v polyethyleneglycol 4000). An acupuncture needle was used to carefully separate single crystals, transferred to a cryoprotectant solution for 5 min (solution # 9 supplemented with 25% glycerol) and then loop-mounted and super-cooled under direct liquid nitrogen at 100 K. *Pp*AK in arginine (Arg-*Pp*AK) binary complex crystal was prepared by soaking apo-*Pp*AK single crystal in the same crystallization solution containing 20 mM arginine by 10 min at 16 °C. The soaked crystal was then super-cooled by using the cryoprotectant solution with arginine, to keep the substrate concentration constant.

### X-ray data collection and crystal structure determination

Apo-*Pp*AK X-ray diffraction experiments were performed in a Bruker D8 QUEST diffractometer equipped with a CuKα Incoatec microfocus sealed tube at 1.54 Å wavelength and a Photon 100 CMOS detector (100 cm^2^). X-ray data were collected at 100 K by omega-phi scan with 30 s exposure time with and oscillation range of 0.5°and the crystal to detector distance was kept at 60 mm. Dataset was integrated, scaled and space group determination by using Proteum II^®^ software suite provided by Bruker. Apo-*Pp*AK crystal belongs to P2_1_ space group with unit cell dimensions of *a* = 48.13, *b* = 61.11, *c* = 60.99; α = γ = 90°and β = 95.86°. Cell content analysis indicates that was one *Pp*AK molecules per asymmetric unit with a *V*_*m*_ = 2.23Å^3^ D^−1^ and solvent content = 44.74% (Matthews, 1968). Whereas, Arg-*Pp*AK crystals were diffracted on beam line 14-1 of the Stanford Synchrotron Light Source at SLAC National Accelerator Laboratory (Menlo Park, CA, USA) using a MARmosaic CCD detector. X-ray data set (386 frames) was collected at λ 0.98 Å with an oscillation range of 0.7°at 100 K. The crystal was exposed to 6 s to the beam and the crystal-to-detector distance was 230 mm. Arg-*Pp*AK data set was analyzed using the XDS package (Kabsch, 2010). Also, Arg-*Pp*AK crystal has the same space group as apo-*Pp*AK crystal and slight differences in cell dimensions and cell content.

Phases were determined by molecular replacement in *Phaser* (McCoy et al., 2005), as part of the CCP4 suite (Winn et al., 2011) using *Litopenaeus vannamei* arginine kinase coordinates as the template (PDB 4AM1) for apo*-Pp*AK. While apo*-Pp*AK was used as the search model for solving phase of Arg-*Pp*AK data set. Refinement was achieved by combining several automated and manual building-cycles using *Phenix* (Adams et al., 2010) and *Coot* (Emsley et al., 2010) programs, respectively. Final models were validated by Molprobity (Adams et al., 2010) and deposited in the Protein Data Bank (apo*-Pp*AK = PDB 5U8E and Arg-*Pp*AK = PDB 5U92). Figures were drawn using CCP4-molecular graphics software (McNicholas et al., 2011). Electron density of ligands was calculated using Polder maps (Liebschner et al., 2017) as implemented in *Phenix*, which removes solvent effects and provides a better representation for ligand into the active site. Quality of the structures was assesed by paremeters like clash score, rotamer outliers, C-β outliers and residues with bad angles and bad bond distances as implemented in Molprobity (Chen et al., 2010).

## Results

During the sequencing of an *P. pythagoricus* cDNA library, a partial cDNA sequence that clearly matched for an arginine kinase was obtained. Using RACE, we obtained a 1068 cDNA sequence that encodes an open reading frame for AK, with a deduced amino acid sequence of 43 kDa protein, with a theoretical isoelectric point of 6.37.

**Figure 1:**
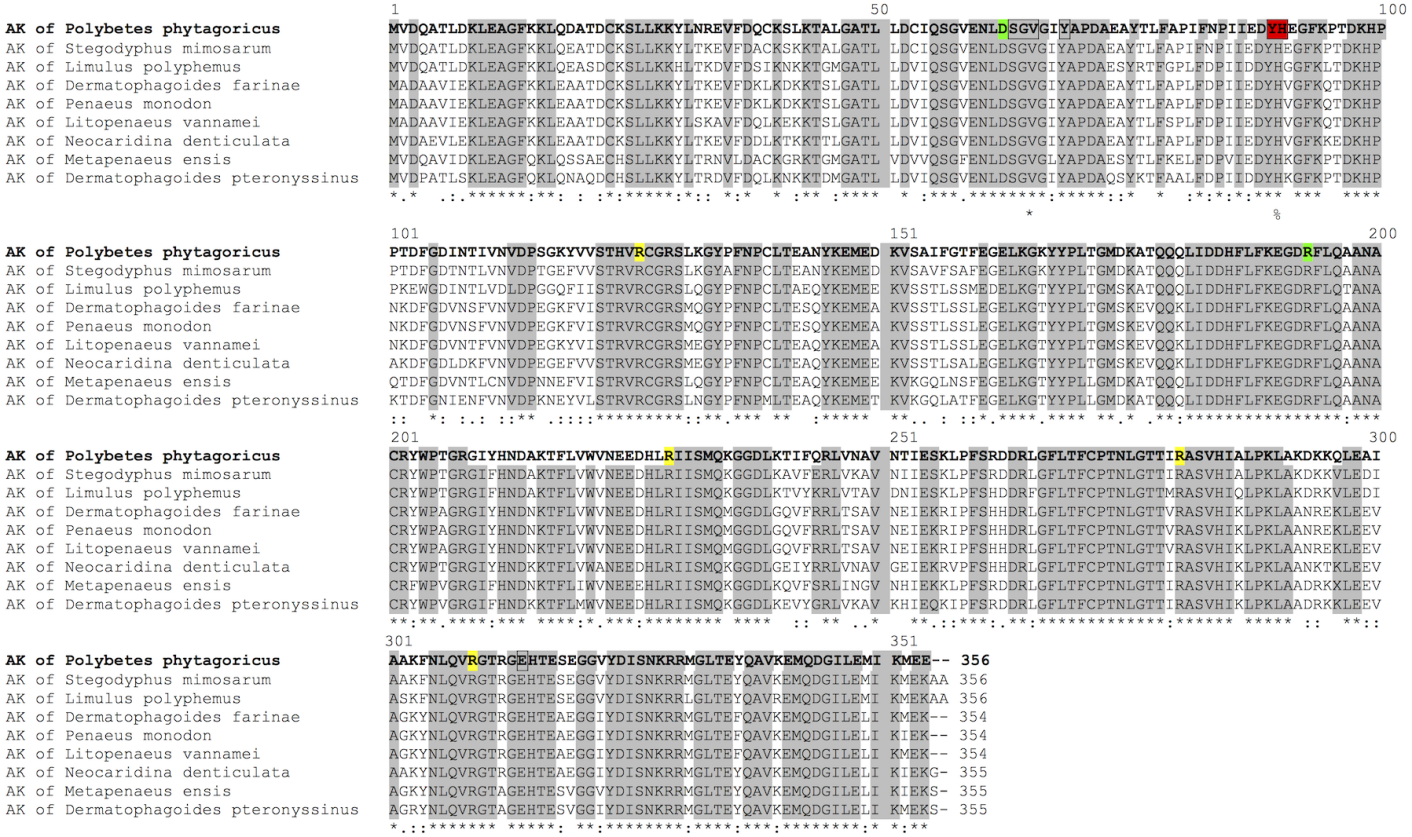
Sequence alignment. Aligment of *Pp*AK of spider *P. pythagoricus* amino acid sequences with AK of: *Stegodyphus mimosarum* (KFM68792.1), *Limulus polyphemus* (NP_001301013.1), *Dermatophagoides farinae* (AAP57094.1), *Penaeus monodon* (AGV55412.1), *Litopenaeus vannamei* (ABY57915.1), *Neocaridina denticulata* (BAH56609.1), *Metapenaeus ensis* (ACA51932.1), *Dermatophagoides pteronyssinus* (ACD50950.1). Arginine and ADP-binding residues in AK from *Limulus* crystal structure (Zhou et al., 1998) are indicated in yellow, respectively; highly conserved residues D62 and R193 (Suzuki et al., 2000) in green, and guanidino group substrate-specific residues (Edmiston et al., 2001; Tanaka & Suzuki, 2004) in red.

AK is highly conserved as observed in a sequence alignment with invertebrates (Fig. 1). The sequence contains the arginine and ADP-binding site, as described for *Limulus* AK, and the highly-conserved residues Asp62 and Arg193 that interact with the substrates (Fig. 1).

Based on the amino acid sequence, an *E. coli* codon-optimized synthetic gene was designed for expression in *E. coli*. An N-terminal histidine tag was included in the design of the synthetic gene to allow amenable purification by nickel affinity chromatography (IMAC). Upon expression in *E. coli* BL21 (DE3) cells, induction of a protein with the expected size was obtained in the soluble fraction. Using IMAC, a single protein was purified with molecular weight of 43 kDa.

The steady-state kinetic analysis of *Pp*AK using L-Arg as substrate led to an apparent Michaelis–Menten kinetic constant (*K*_*m*_) was 1.7 mM; *V*_max_ was and 27.8 μmol min^−1^, and *k*_cat_ was 75 s^−1^. Table 1 shows in comparative form the kinetics of AK in relation to other arthropods.

**Table 1:** Comparison of kinetic parameters of different arthropod AKs.

Species	Reference	${K}_{m}^{\mathrm{Arg}}$ (mM)	*k*_cat_ (s^−1^)	${k}_{\mathrm{cat}}{\mathop{{/ K}}\nolimits }_{m}^{\mathrm{Arg}}$ (s^−1^ mM^−1^)
***Polybetes pythagoricus* (spider)**	**Present study**	**1.7**	**75**	**44.1**
*Dugesiella hentzi* (spider)	Blethen (1972)	0.27		
*Limulus polyphemus* (horseshoe crab)	Blethen (1972)	0.35		
*Palamneus phipsoni* (scorpion)	Arjunwadkar & Raghupathi Rami Reddy (1985)	0.08		
*Locusta migratoria manilensis* (Insect)	Wu et al. (2007)	0.95	159.4	169
*Cissites cephalotes* (insect)	Tanaka et al. (2007)	1.01	2.02	2.01
*Periplaneta americana* (insect)	Brown & Grossman (2004).	0.49	1.30	2.65
*Antarctic krill* (Crustacea)	Si et al. (2014)	0.30	84.8	282.7
*Litopenaeus vannamei* (marine shrimp)	Lopez-Zavala et al. (2016)	0.32		

**Figure 2:**
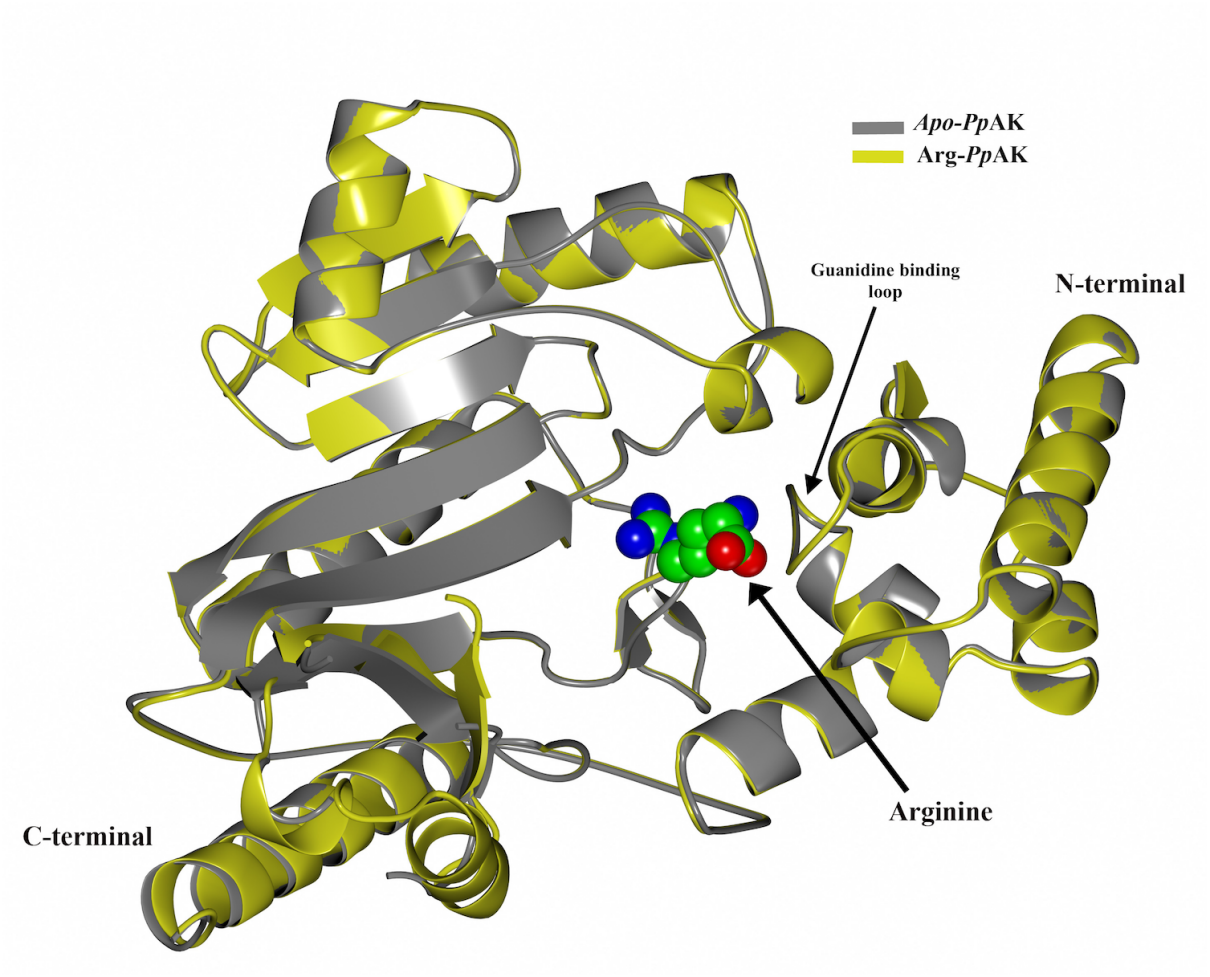
Structural alignment. Superposition of crystallographic structures of free-ligand (*grey*) and arginine binary complex (*yellow*) *Pp*AK. The arginine substrate is shown as spheres colored by atom type (*green for carbon, blue for nitrogen and red for oxygen*) in the hinge between N-terminal and C-terminal domains.

Besides the biochemical characterization, we obtained the crystallographic structures of *Pp*AK (Fig. 2) in substrate-free from (apo*-Pp-*AK) and in a binary complex with the guanidino group substrate-arginine (Arg-*Pp*AK) at 2.1 and 2.0 Å resolution, respectively (Table 2). Both structures crystallized in the same spacegroup and were solved by the molecular replacement method using the *Litopenaeus vannamei* (marine Pacific shirmp) arginine kinase (PDB 4AM1) as the search model (Lopez-Zavala et al., 2012). Structures were well refined and good stereochemical parameters were obtained (Table 3).

**Table 2:** Data reduction statistics of *Pp*AK structures. Values in parenthesis represent the statistics at the highest resolution bin.

Data set	apo-*Pp*AK	arg-*Pp*AK
X-ray source	Bruker D8-Quest	SSRL Beam line-14-1
Detector	Photon 100 CMOS	MAR-Mosaic 325 CCD
Wavelenght (Å)	1.54 Å	0.98 Å
Space group	P2_1_	P2_1_
Unit-cell parameters (Å)	*a* = 48.13, *b* = 61.11, *c* = 60.99	*a* = 48.09.3, *b* = 61.45, *c* = 60.6
	β = 95.86^°^	β = 95.75^°^
Number of residues	357	357
Monomers per asymmetric unit	1	1
Mathews coefficient (Å Da^−1^)	2.23	2.21
Solvent content (%)	44.74	44.22
Resolution range (Å)	20.3–2.18 (2.25–2.18)	19.73–2.0 (2.07–2.0)
Total reflections	246,281 (18,398)	130,527 (12,408)
Unique reflections	35,766 (4,274)	23,798 (2,308)
Redundancy	6.8 (4.3)	5.5 (5.4)
Completeness (%)	99.43 (94.27)	99.79 (98.80)
Mean *I*∕σ(*I*)	24.7 (3.34)	17.5 (5.64)
CC_1∕2_	0.98 (0.739)	0.99 (0.97)
R_symm_^a^	0.0847 (0.389)	0.0635 (0.293)
R_meas_^b^	0.0476 (0.2845)	0.0703 (0.328)
Wilson plot *B* value	19.38	24.9

**Notes.**

a R_symm_ = ∑_*hkl*_∑_*i*_|*I*_*i*_(*hkl*) − (*I*(*hkl*))|∕∑_*hkl*_∑_*i*_*I*_*i*_(*hkl*), where *I*_*i*_(*hkl*) and (*I*(*hkl*)) represent the diffraction-intensity values of the individual measurements and the corresponding mean values. The summation is over all unique measurements.

b R_meas_ is a redundancy-independent version of R_symm_, R${}_{\mathrm{meas}}={\sum }_{h}\sqrt{}{n}_{h}/{n}_{h}-1{\mathop{\sum }\nolimits }_{\mathrm{i}}^{\mathrm{nh}}{|}{\hat {I}}_{\mathrm{h}}$ –I ${}_{\mathrm{h,i}}{|}/{\sum }_{h}{\mathop{\sum }\nolimits }_{\mathrm{i}}^{\mathrm{nh}}{I}_{\mathrm{h,i}}$, where ${\hat {I}}_{\mathrm{h}}=1/{n}_{\mathrm{h}}{\mathop{\sum }\nolimits }_{\mathrm{i}}^{\mathrm{nh}}{I}_{\mathrm{h,i}}$.

**Table 3:** Refinement statistics. Crystallographic refinement statistics of *Pp*AK structures.

Data set	apo-*Pp*AK	Arg-*Pp*AK
R_work_/R_free_(5%)	0.1747/0.2316	0.1679/0.2365
Content of asymetric unit		
Protein residues	340	348
Ligands	Sodium	Arginine, sodium
Water molecules	278	338
RMSD from ideal		
Bond length (Å)	0.008	0.007
Bond angles (°)	1.07	1.034
Mean overall *B* value (Å^2^)		
Protein	22.3	27.8
Solvent	30.40	38.7
Ramachandran plot, residues in		
Most favored regions	333 (98%)	341 (98%)
Additionally allowed regions	7 (2.10%)	7 (2.06%)
Outliers	1 (0.3%)	0 (0%)
Clash score^a^ (all atoms)	5.0	2.93
Rotamer outliers (%, goal < 1%)	0	0.34
C-β outliers devations > 0.25 Å(goal 0)	0	0
Residues with bad bonds (%, goal < 1%)	0	0
Residues with bad angles (%, goal < 0.5%)	0	0.03
PDB code	5U8E	5U92

**Notes.**

a Clash score is the number of serious steric overlaps (>0.4 Å) per 1,000 atoms.

RMSD root mean square deviation

In AKs and in phosphagen kinases in general, the canonical fold is comprised of two domains; a small N-terminal globular domain and a large C-terminal α-β domain. In *Pp*AK, the N-terminal domain (residues 1–100), is comprised of α-helices and the amino acids residues involved in arginine substrate binding (Fig. 2). This domain is classified as the ATP:guanido phosphotransferase N-terminal domain on the InterPro database (IPR022413) (Finn et al., 2017), or as the transferase creatine kinase superfamily 1.10.135.10 in the CATH (Class-Architecture-Topology-Homologous superfamily) protein structure classification (Dawson et al., 2017).

The large C-terminal domain (residues 101–357), is formed by an α-β fold and contains the ATP-binding site. It is classified as the ATP:guanido phosphotransferase, catalytic domain (IPR022414) or as the CATH superfamily 3.30.590.10.

Interestingly, the active site is located between both domains and a large conformational change occurs during catalysis as a hinged rotation (Zhou, Ellington & Chapman, 2000). The crystallographic model includes 340 of the 357 amino acids deduced from the cDNA sequence. Two loops (292–297 and 310–320) show weak electron density and therefore those residues are not present in the final structural model. The guanidino group binding loop (Asp62, Ser63, Gly64 and Val65) and stabilizing residues (Try68, Cys271 and Glu225) also are well-ordered. 347 amino acid residues were fitted in the final model (see Tables 2 and 3 for statistical details), as in apo-*Pp*AK structure residues 310–320 are disordered. Arg-*Pp*AK binary complex was found is the *open* conformation similar to the free substrate *Pp*AK model (Lopez-Zavala et al., 2013). Superposition of both structures do not show overall conformational changes upon arginine binding (RMSD = 0.36 Å for C-α atoms) (Fig. 2).

**Figure 3:**
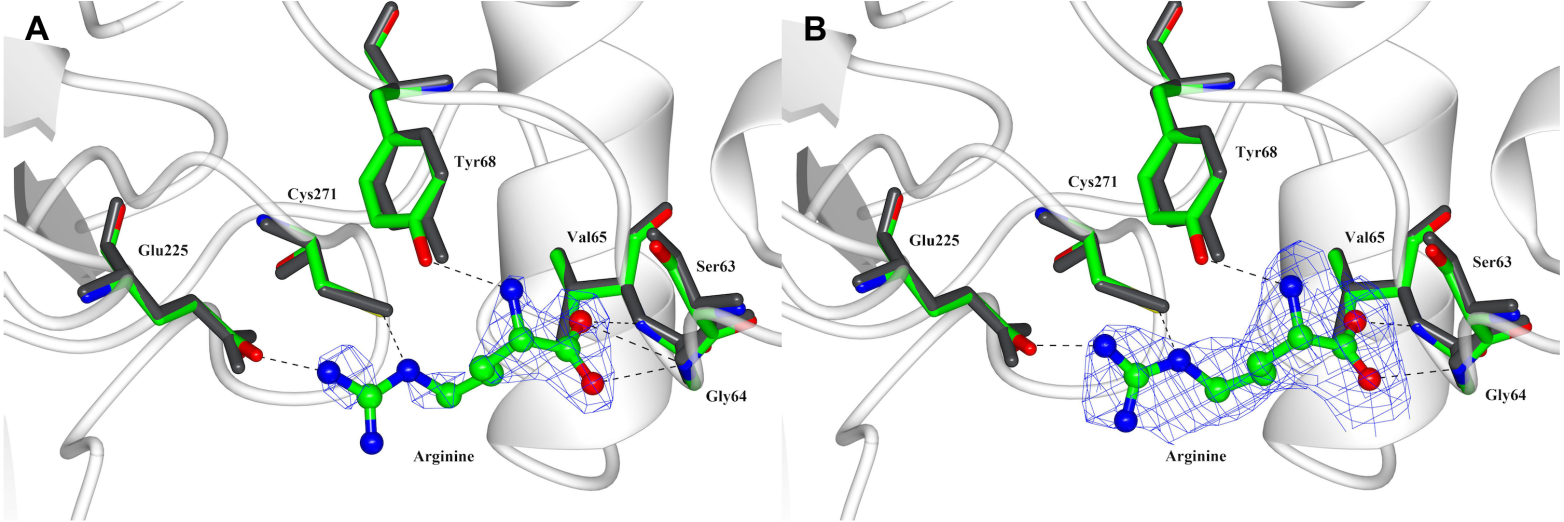
Arginine binding-site of *Pp*AK. Arginine substrate (ball-sticks colored by atom type) is stabilized by the guanidino group binding loop (Gly64 and Val65), Cys 271 and Glu225. All amino acids are presented as cylinders colored by atom type (Arg-*Pp*AK) and in dark-grey (apo-*Pp*AK). Hydrogen bonds are shown as a dotted line. (A) Electron density 2Fo-Fc map of arginine is displayed as a blue mesh with a 1.5 σ contour level. (B) Polder omit density map of arginine is displayed as a blue mesh with a 3 σ contour level.

The structure of substrate-bound *Pp*AK was obtained by soaking crystals of the apoAK in 20 mM arginine dissolved in the crystallization solution. The electron density was weak in a regular electron density map, although the substrate could be modelled in the arginine-binding site (Fig. 3A). Using a Polder map, which is an omit map where the bulk solvent is excluded during calculations, generated a much better electron density that supports modeling L-arginine into the active site and the interactions with *Pp*AK side chains (Fig. 3B).

Arginine-substrate carboxylate is stabilized by hydrogen bonds to backbone Gly64, Val65 and Gly66 and the hydroxyl group of Tyr68 to arginine amino group. The guanidino group of the arginine substrate has specific contacts in the active site. A salt bridge with Glu225 and a hydrogen bond with Cys271 position the substrate in a region with poor density. The sidechains around the C α carbons are better ordered and have contacts with Tyr68, Ser63 and Gly64 (Fig. 3B).

Also, a superposition of N-terminal domain of both *Pp*AK structures we found that all residues involved in arginine binding-site are in the same position (Fig. 3). Comparing the apo *vs*. Arg-bound *Pp*AK structures, it is observed that the Glu225 carboxylate lateral chain is slightly rotated to favour a hydrogen bond of arginine guanidino group end (Fig. 3). As mentioned before, in both apo- and *Pp*AK-Arg structures, the loop 310–320 was disordered. A structural similarity search of *Pp* AK-Arg using the *Dali* server (Holm & Rosenstrom, 2010) against the PDB database, we found that the most similar structure was the shrimp apo-AK, (PDB entry 4AM1, RMSD 1.0 Å). The most similar fully occupied active site AK was the one from the horseshoe crab (*Limulus polyphemus* transition state complex, PDB entry 1M15, RMSD = 2.86 Å).

## Discussion

Although spiders are one of the most significant and numerous orders of the arachnid class, they are underrepresented in terms of molecular and structural information. At this time, the information regarding the AK from spiders is restricted to only a few species, such as *Holocnemus pluchei*, (Bobolea et al., 2011), *Pholucus phalmgioides* and *Dugesiella hentzi* (Blethen & Kaplan, 1968). In this work, we sequenced, cloned and overexpressed a spider arginine kinase (PpAK), an enzyme crucial for rapid energy energy fluxes in invertebrates.

Based on the cDNA *Pp*AK sequence, the recombinant enzyme was overexpressed showing an electrophoretical molecular mass of 40 kDa, similar to the AKs of arthropods, horseshoe crab, crustaceans and insects (Rosenthal, Dahlman & Robinson, 1977; Wang et al., 2009b). In relation to the calculated isoelectric point pH 6.4 of expressed *Pp*AK which is different to native AK from lobster *Homarus vulgaris* at pH 5.4 (Virden, Watts & Baldwin, 1965), and expressed AK2 and AK3 from shrimp *Neocaridina denticulata* at pH 5.8, 6.0 (Iwanami et al., 2009), but similar to the AK from insect *Locusta migratoria manilensis* pH 6.3 (Wu et al., 2007) and AK from the crustacean *N. denticulata* (AK1) pH 6.3 (Iwanami et al., 2009).

A multiple alignment revealed that *Pp*AK has the highest identity with the unique AK sequence from the spider *Stegodyphus mimosarum* (93%). Although *P. pythagoricus* and *S. mimosarum* share the araneomorphae suborder they belong to two phylogenetically distant families. The AK from *Limulus polyphemus* (the horseshoe crab) shares 77% identity which is expected for an arthropod in the class Chelicerata as they diverged early in the evolution of insect and crustaceans (Strong & Ellington, 1995), and is considered one of the oldest/primitive arthropods.

Since AK is related in function to creatine kinase of vertebrate, glycocyamine kinase of invertebrates, lombricine of annelida and taurocyamine kinase of protics and invertebrates (Dumas & Camonis, 1993; Suzuki et al., 2000), the relationship of these different enzymes with AK possibly due to these different enzymes evolved from a common ancestor (Morrison, 1973; Perovic-Ottstadt et al., 2005; Suzuki & Furukohri, 1994).

*Pp*AK expression in *E. coli* and determination of enzymatic activity in the purified recombinant protein showed that the gene encodes an active AK. The specific activity of the recombinant protein (14 U/mg) is similar to that obtained from AK in muscle of the spider *Pholucus phalangoides* (15.3 U/mg) (Blethen & Kaplan, 1968) and in the claw muscle of the scorpion *Palamneus phipsoni* (14.1 U/mg) (Arjunwadkar & Raghupathi Rami Reddy, 1985). It also matches with other recombinant AKs: fly *Lucilia cuprina* 14 U/mg (Ilg & Werr, 2012), *Musca domestica* (10.3 U/mg) (Rockstein & Kumar, 1972). Furthermore, it is close to the activity values of the AK purified from muscle from *Litopenaeus vannamei* (8.8 U/mg) (García-Orozco et al., 2007) and *Homarus americanus* (15.6 U/mg) (Blethen & Kaplan, 1968). The specific activities of the tarantula *Dugestella hentzi* (58.5 and 75 U/mg) (Blethen & Kaplan, 1968) and activity of the recombinant AK from the insect *Ctenocephalides felis* 75 U/mg (Werr, Cramer & Ilg, 2009) were much higher. The variations seen may be due to differences in experimental techniques.

The kinetic parameters determined for the substrate L-arginine were *K*_*m*_ 1.7 mM and *V*_max_ 27.8 μmol min^−1^ mg protein^−1^; within the range described for other arthropods. The horseshoe crab *Limulus polyphemus* (Blethen, 1972), the scorpion *Palamneus phipsoni* (Arjunwadkar & Raghupathi Rami Reddy, 1985) and tarantula *Dugesiella hentzi* (Blethen & Kaplan, 1968) had a *K*_*m*_ between 0.08 and 0.67 mM; in insects *Cissites cephalotes* (Tanaka et al., 2007), *Locusta migratoria* (Ilg & Werr, 2012; Wu et al., 2007), *Periplaneta americana* (Brown & Grossman, 2004), *K*_*m*_ was between 0.49–1.0 mM; and in the crustacean *Litopenaeus vannamei* (Lopez-Zavala et al., 2016), *Euphausia superba* (Si et al., 2014), the *K*_*m*_ was 0.3 (Table 1).

The value of *k*_cat_ calculated for *Pp*AK (as shown in Table 1) is within the range reported in arthropods, which seems to be highly variable from 1.3 s^−1^ in *P. americana* (Brown & Grossman, 2004) to 159 s^−1^ for recombinant AK and AK from *L. migratoria* leg muscle (Wu et al., 2007). The *Pp*AK catalytic efficiency for arginine (*k*_*ca*__t_∕*K*_*m*_ Arg) was 44.1 s^−1^ mM^−1^, well within the range reported in arthropods: 2.01 s^−1^ mM^−1^ for *C. cephalotes* (Tanaka et al., 2007) to 282.7 s^−1^ mM^−1^ for *Euphausia superba* (Si et al., 2014).

Structural characterization has been focused in both free-ligand and fully occupied active site of AK, that are called *open* and *closed* conformation, respectively (Fernandez et al., 2007; Pruett et al., 2003; Zhou et al., 1998). Only, a few reports show structural details of binding of arginine in a binary complex, which is a crucial step during catalysis (Lopez-Zavala et al., 2013; Wang et al., 2015). To date, horseshoe crab crystal structure has been reported for Chelicerate, which is comprised of more than 75,000 species (Strong & Ellington, 1995). Apo-*Pp*AK structure was found in the *open* conformation as reported for other phosphagen kinases in the substrate-free form (Fig. 3). In this conformation, the loop comprised from amino acid residue 310–320 is disordered and therefore, was not present in final experimental model. Conversely, this loop is well ordered and serves as a *lid* to the active site when both substrates are the catalytic closed conformation.

The guanidino group binding loop (residues 59–64 ENLDSG for *Pp*AK) in the phosphagen kinases are invariant and are located in the N-terminal domain. The length of this loop is related to the size of the substrate (Azzi et al., 2004). *Pp*AK has a well-conserved guanidino group binding loop (see Fig. 2) that is indicative of its preferred substrate, arginine. The binary complex of *Pp*AK and L-Arg has a similar conformation compared to the unbound structure (Fig. 3). This suggest that the enzyme has a conformation ready to bind the guanidino group and the loop sequence is optimal for ligand binding. In more detail, there are precise hydrogen bonds with residues of the guanidino group binding loop (Fig. 3). Specifically, arginine carboxylate groups are in contact with the amide backbone of Gly64, Val65 and Gly66; also, the hydroxyl group of Tyr68 stabilizes the arginine amide. Another highly conserved residue is Cys271, which has been implicated in catalysis (Buechter et al., 1992; Marletta & Kenyon, 1979). In *Pp*AK this residue interacts via hydrogen bonds with N(ε) guanidino group. Glu225 has been proposed as one of two bases that assist acid–base catalysis mechanisms in phosphagen kinases by substrate alignment during phosphate group transference (Pruett et al., 2003). *Litopenaeus vannamei* crystal structure in arginine binary complex (*Lv* AK-Arg; PDB entry 4BHL) shows that Glu225 stabilizes the arginine guanidino group end by two ionic bonds in a pre-stabilized catalytic intermediate (Lopez-Zavala et al., 2013). In the *Pp*AK-Arg structure we found that Glu225 is positioned in a similar way, making only one salt-bridge with NH_2_(ω)-1 of the arginine guanidino group end. This small local conformational change was also observed in *Lv*AK-Arg structure and it is postulated to lead to a productive active site in the closed conformation (López-Zavala et al., 2014; Zhou et al., 1998).

In spiders, the resting metabolic rate is much lower compared to other animals (Anderson, 1970), about half of the expected rate for other poikilotherm (Schmitz, 2016). Fast movements are limited by depletion of phosphagens, accumulation of lactate and a limited aerobic metabolism (Prestwich, 1988), besides a low number of mitochondrias in the muscle and the respiratory system (Figueroa et al., 2010). Biochemical studies realized in spiders indicate low activity of mitochondrial citrate synthase, glutamate dehydrogenase and isocitrate synthase, suggesting a low aerobic metabolism (Linzen & Gallowitz, 1975). Also in spiders, the structure of muscle fiber suggests that most of the fiber is comprised of the contractile apparatus, with limited energy production. This in contrast to the flight muscle of *Locusta migratoria*, where 40% of the volume in muscle fibers comes from mitochondria (Linzen & Gallowitz, 1975).

## Conclusion

In conclusion, we successfully studied molecular, enzymatic and structural aspects of the arginine kinase from the spider *P. pythagoricus*. Our resolved structures are the first views of AK in arachnids. Both ligand-free and arginine binary complex were found in the *open* conformation. The *Pp*AK guanidino group binding site appears to be pre-organized to accept the arginine substrate since it overlaps with the unbound crystal structure. Binding of the second substrate is needed for the conformational changes to occur in the AK structure of other invertebrates. Finally, these results contribute to increase the basic knowledge of spider bioenergetics.

##  Supplemental Information

10.7717/peerj.3787/supp-1 Supplemental Information 1 Crystallographic coordinates of apo *Pp*AK

10.7717/peerj.3787/supp-2 Supplemental Information 2 MolProbity report for the *Pp*AK structure bound to Arg, final structure

10.7717/peerj.3787/supp-3 Supplemental Information 3 Coordinates of the arginine-bound *Pp* AK

10.7717/peerj.3787/supp-4 Supplemental Information 4 MolProbity report of the final ApoAK 5u8E structyre

10.7717/peerj.3787/supp-5 Supplemental Information 5 Binary structure factors in CCP4 MTZ file format for *Pp* apo AK

10.7717/peerj.3787/supp-6 Supplemental Information 6 Supplemental materials text

10.7717/peerj.3787/supp-7 Supplemental Information 7 Binary structure factors in CCP4 MTZ file format for arginine-bound *Pp* AK

## Additional Information and Declarations

### DNA Deposition

The following information was supplied regarding the deposition of DNA sequences: The nucleotide and deduced amino acid sequence were deposited at GenBank under the accession number MF001441.

### Data Availability

The following information was supplied regarding data availability: The coordinates and structure factors deposited at PDB are included as Supplemental Files. The assigned PDB codes are 5U8E and 5U92.
