# Supplementary material for: Biochemical and structural characterization of a novel arginine kinase from the spider Polybetes pythagoricus
Source: PeerJ. 2017 Sep 11;5:e3787. doi: 10.7717/peerj.3787 (PMC5598448; doi:10.7717/peerj.3787)
Supplement: Supplemental Information 2 [file peerj-05-3787-s002.pdf]

M[illegible]

# Viewing spiderAK-ARG_refine_47FH-multi.table

When finished, you should close this window .

*Hint: Use File | Save As... to save a copy of this page.*

| | | | | |
|---|---|---|---|---|
| All-Atom Contacts | Clashscore, all atoms: | 2.93 | | 99th percentile* (N=715, 2.000Å ± 0.25Å) |
| | Clashscore is the number of serious steric overlaps (> 0.4 Å) per 1000 atoms. | | | |
| Protein Geometry | Poor rotamers | 2 | 0.68% | Goal: <0.3% |
| | Favored rotamers | 270 | 91.84% | Goal: >98% |
| | Ramachandran outliers | 0 | 0.00% | Goal: <0.05% |
| | Ramachandran favored | 334 | 98.24% | Goal: >98% |
| | MolProbity score^ | 1.08 | | 100th percentile* (N=12522, 2.000Å ± 0.25Å) |
| | Cβ deviations >0.25Å | 0 | 0.00% | Goal: 0 |
| | Bad bonds: | 0 / 2800 | 0.00% | Goal: 0% |
| | Bad angles: | 1 / 3778 | 0.03% | Goal: <0.1% |
| Peptide Omegas | Cis Prolines: | 1 / 15 | 6.67% | Expected: ≤1 per chain, or ≤5% |

In the two column results, the left column gives the raw count, right column gives the percentage.
* 100th percentile is the best among structures of comparable resolution; 0th percentile is the worst. For clashscore the comparative set of structures was selected in 2004, for MolProbity score in 2006.
^ MolProbity score combines the clashscore, rotamer, and Ramachandran evaluations into a single score, normalized to be on the same scale as X-ray resolution.

| # | Alt | Res | High B | Clash > 0.4Å | Ramachandran | Rotamer | Cβ deviation | Bond lengths | Bond angles | Cis Peptides |
|---|---|---|---|---|---|---|---|---|---|---|
| | | | Avg: 34.13 | Clashscore: 2.93 | Outliers: 0 of 340 | Poor rotamers: 2 of 294 | Outliers: 0 of 323 | Outliers: 0 of 348 | Outliers: 1 of 348 | Non-Trans: 1 of 345 |
| A 1 | | ALA | 39.83 | 0.47Å N with S 251 HOH O | - | - | 0.01Å | - | - | - |
| A 2 | | VAL | 36.16 | - | Favored (24.63%) Ile or Val / -78.1,136.2 | Favored (83.8%) *t* chi angles: 177 | 0.07Å | - | - | - |
| A 3 | | ASP | 45.67 | - | Favored (37.17%) General / -75.4,154.3 | Favored (46%) *p0* chi angles: 65.9,348.1 | 0.04Å | - | - | - |
| A 4 | | GLN | 37.12 | - | Favored (71.05%) General / -62.8,-29.9 | Favored (77.3%) *tp40* chi angles: 184,68.3,41.7 | 0.05Å | - | - | - |

| | | | | | | | | | |
|---|---|---|---|---|---|---|---|---|---|
| A 5 | ALA 35.34 | - | Favored (97.9%) General / -63.7,-42.5 | - | 0.02Å | - | - | - |
| A 6 | THR 34.43 | - | Favored (86.6%) General / -67.2,-40.2 | Favored (47.6%) *m* chi angles: 304.4 | 0.03Å | - | - | - |
| A 7 | LEU 36.01 | - | Favored (88.36%) General / -61.5,-38.8 | Favored (93.8%) *mt* chi angles: 294.2,171.6 | 0.04Å | - | - | - |
| A 8 | ASP 38.21 | - | Favored (85.1%) General / -67.0,-42.4 | Favored (51.3%) *m-30* chi angles: 278.6,340.8 | 0.02Å | - | - | - |
| A 9 | LYS 41.09 | - | Favored (98.32%) General / -63.3,-40.8 | Allowed (0.9%) *mttt* chi angles: 270.7,146.3,201.5,136.9 | 0.02Å | - | - | - |
| A 10 | LEU 36.56 | - | Favored (81.3%) General / -59.0,-48.5 | Favored (28.4%) *mt* chi angles: 282.2,171 | 0.03Å | - | - | - |
| A 11 | GLU 38.06 | - | Favored (83.59%) General / -62.7,-36.6 | Favored (52.4%) *mm-30* chi angles: 287.5,283.9,358.4 | 0.02Å | - | - | - |
| A 12 | ALA 37.73 | - | Favored (75.25%) General / -69.7,-35.8 | - | 0.02Å | - | - | - |
| A 13 | GLY 37.35 | - | Favored (70.47%) Glycine / -66.5,-47.3 | - | - | - | - | - |
| A 14 | PHE 39.36 | 0.41Å O with A 18 GLN HG2 | Favored (77.63%) General / -56.4,-48.1 | Favored (72.9%) *t80* chi angles: 174.8,85.7 | 0.01Å | - | - | - |
| A 15 | LYS 41.89 | - | Favored (91.82%) General / -60.8,-40.2 | Allowed (1.3%) *tttm* chi angles: 154.1,146.4,198.7,293.3 | 0.01Å | - | - | - |
| A 16 | LYS 41.8 | - | Favored (75.82%) General / -62.6,-33.8 | Favored (18.2%) *tttt* chi angles: 175.7,172.2,182.6,212.8 | 0.02Å | - | - | - |
| A 17 | LEU 38.38 | - | Favored (63.13%) General / -74.3,-33.4 | Favored (28.9%) *mt* chi angles: 285.6,159.9 | 0.02Å | - | - | - |
| A 18 | GLN 44.72 | 0.41Å HG2 with A 14 PHE O | Favored (62.9%) General / | Favored (14.6%) *mm-40* chi angles: | 0.04Å | - | - | - |

| | | | | | | | | | | |
|---|---|---|---|---|---|---|---|---|---|---|
| | | | | | -72.6,-28.7 | 324.4,313.6,308.6 | | | | |
| A 19 | | ASP | 44.62 | - | Favored (58.06%) General / -82.5,-12.0 | Favored (79.5%) *m-30* chi angles: 294,349.8 | 0.02Å | - | - | - |
| A 20 | | ALA | 43.88 | - | Favored (6.29%) General / -90.8,76.8 | - | 0.03Å | - | - | - |
| **#** | **Alt** | **Res** | **High B** | **Clash > 0.4Å** | **Ramachandran** | **Rotamer** | **Cβ deviation** | **Bond lengths** | **Bond angles** | **Cis Peptides** |
| | | | Avg: 34.13 | Clashscore: 2.93 | Outliers: 0 of 340 | Poor rotamers: 2 of 294 | Outliers: 0 of 323 | Outliers: 0 of 348 | Outliers: 1 of 348 | Non-Trans: 1 of 345 |
| A 21 | | THR | 46.84 | - | Favored (65.73%) General / -59.0,-27.7 | Favored (70.9%) *p* chi angles: 59.3 | 0.06Å | - | - | - |
| A 22 | | ASP | 47.1 | - | - | Favored (59.5%) *m-30* chi angles: 288.7,319.7 | 0.01Å | - | - | - |
| A 23 | | CSO | 41.03 | - | - | - | 0.06Å | - | - | - |
| A 24 | | LYS | 57.67 | - | - | Allowed (1.2%) *mttp* chi angles: 280.1,130.3,163.7,51 | 0.03Å | - | - | - |
| A 25 | | SER | 30.41 | - | Favored (32.8%) General / -74.6,158.9 | Favored (75.6%) *p* chi angles: 59.9 | 0.04Å | - | - | - |
| A 26 | | LEU | 26.73 | - | Favored (73.62%) General / -62.2,-32.9 | Favored (76.3%) *mt* chi angles: 299.7,181.4 | 0.04Å | - | - | - |
| A 27 | | LEU | 25.22 | - | Favored (75.87%) General / -55.7,-48.0 | Favored (75.5%) *tp* chi angles: 177.3,62.4 | 0.01Å | - | - | - |
| A 28 | | LYS | 39.43 | - | Favored (77.36%) General / -68.5,-35.3 | Allowed (0.6%) *tttm* chi angles: 181.7,225.7,205.5,324.4 | 0.02Å | - | - | - |
| A 29 | | LYS | 28.74 | - | Favored (99.69%) General / -62.4,-42.8 | Favored (65.1%) *tttt* chi angles: 175.9,167.9,192.7,183.3 | 0.03Å | - | - | - |
| A 30 | | TYR | 27.59 | - | Favored (16.49%) General / -106.4,-9.9 | Favored (75.7%) *m-80* chi angles: 297,290.3 | 0.04Å | - | - | - |
| A 31 | | LEU | 32.73 | - | Favored (10.23%) General / -86.5,72.7 | Favored (32.6%) *tp* chi angles: 186,66.1 | 0.04Å | - | - | - |

| | | | | | | | | | | |
|---|---|---|---|---|---|---|---|---|---|---|
| A 32 | | ASN | 36.58 | - | Favored (7.11%) General / -84.4,-179.2 | Favored (34.8%) *p0* chi angles: 62.8,39.9 | 0.06Å | - | - | - |
| A 33 | | ARG | 30 | - | Favored (92.14%) General / -65.4,-39.5 | Favored (3.9%) *tpt170* chi angles: 182.3,74.9,217.1,185.7 | 0.02Å | - | - | - |
| A 34 | | GLU | 36.53 | - | Favored (69.73%) General / -71.8,-39.9 | Favored (3.9%) *tp30* chi angles: 198.1,45.9,67.9 | 0.01Å | - | - | - |
| A 35 | | VAL | 31.29 | - | Favored (95.62%) Ile or Val / -63.9,-42.4 | Favored (51.9%) *t* chi angles: 169.7 | 0.06Å | - | - | - |
| A 36 | | PHE | 32.71 | - | Favored (95.02%) General / -60.6,-45.2 | Favored (85.5%) *t80* chi angles: 181.3,76.1 | 0.03Å | - | - | - |
| A 37 | | ASP | 35.85 | - | Favored (67.97%) General / -69.8,-30.4 | Favored (4.6%) *m-30* chi angles: 274.5,304.8 | 0.03Å | - | - | - |
| A 38 | | GLN | 33.08 | - | Favored (84.94%) General / -59.1,-40.3 | Favored (35.4%) *mt0* chi angles: 284.1,183.9,261.8 | 0.06Å | - | - | - |
| A 39 | | CYS | 31.11 | - | Favored (13.58%) General / -102.0,-26.0 | Favored (94.9%) *m* chi angles: 292 | 0.05Å | - | - | - |
| A 40 | | LYS | 34.66 | - | Favored (67.72%) General / -64.7,-24.9 | Favored (8.2%) *pttm* chi angles: 65.7,181.1,205,289.8 | 0.01Å | - | - | - |
| **#** | **Alt** | **Res** | **High B** | **Clash > 0.4Å** | **Ramachandran** | **Rotamer** | **Cβ deviation** | **Bond lengths** | **Bond angles** | **Cis Peptides** |
| | | | Avg: 34.13 | Clashscore: 2.93 | Outliers: 0 of 340 | Poor rotamers: 2 of 294 | Outliers: 0 of 323 | Outliers: 0 of 348 | Outliers: 1 of 348 | Non-Trans: 1 of 345 |
| A 41 | | SER | 37.81 | - | Favored (36.8%) General / -102.6,12.1 | Allowed (0.9%) *m* chi angles: 272.6 | 0.04Å | - | - | - |
| A 42 | | LEU | 31.51 | - | Favored (17.19%) General / -92.9,156.0 | Favored (54.6%) *mt* chi angles: 303.9,173.1 | 0.02Å | - | - | - |
| A 43 | | LYS | 34.53 | - | Favored (9.36%) General / -153.7,128.3 | Favored (20%) *ttmt* chi angles: 180.6,187.1,271.5,177.8 | 0.01Å | - | - | - |

| | | | | | | | | |
|---|---|---|---|---|---|---|---|---|
| A 44 | THR 31.89 | - | Favored (9.13%) General / -67.2,169.1 | Favored (50.5%) *p* chi angles: 65.6 | 0.05Å | - | - | - |
| A 45 | ALA 33.83 | - | Favored (67.24%) General / -66.7,-26.9 | - | 0.03Å | - | - | - |
| A 46 | LEU 34.85 | - | Favored (57.83%) General / -87.4,-1.3 | Favored (79.2%) *mt* chi angles: 293.5,167 | 0.02Å | - | - | - |
| A 47 | GLY 32.06 | - | Favored (44.64%) Glycine / 104.2,1.0 | - | - | - | - | - |
| A 48 | ALA 28.96 | - | Favored (44.94%) General / -73.1,137.1 | - | 0.03Å | - | - | - |
| A 49 | THR 27.09 | - | Favored (4.71%) General / -110.4,177.4 | Favored (2.3%) *p* chi angles: 81.2 | 0.05Å | - | - | - |
| A 50 | LEU 28.53 | - | Favored (71.46%) General / -63.4,-30.3 | Favored (69.6%) *tp* chi angles: 176.3,62.9 | 0.03Å | - | - | - |
| A 51 | LEU 29.62 | - | Favored (89.72%) General / -63.6,-38.1 | Favored (24.7%) *tp* chi angles: 189.6,61.8 | 0.01Å | - | - | - |
| A 52 | ASP 24.33 | - | Favored (23.96%) General / -65.8,-8.9 | Favored (60.6%) *m-30* chi angles: 302.4,309.2 | 0.01Å | - | - | - |
| A 53 | CYS 25.9 | - | Favored (5.28%) General / -94.0,-50.5 | Favored (50.4%) *m* chi angles: 303.5 | 0.03Å | - | - | - |
| A 54 | ILE 24.47 | - | Favored (9.73%) Ile or Val / -102.2,-2.0 | Favored (7.7%) *pt* chi angles: 71.4,183.5 | 0.05Å | - | - | - |
| A 55 | GLN 25.37 | - | Favored (84.92%) General / -61.5,-37.8 | Favored (6.5%) *pt0* chi angles: 53.5,195.4,346.8 | 0.03Å | - | - | - |
| A 56 | SER 26.46 | - | Favored (84.72%) General / -66.0,-37.0 | Favored (38.1%) *t* chi angles: 181.2 | 0.01Å | - | - | - |
| A 57 | GLY 27.65 | - | Favored (79.71%) Glycine / -77.7,-16.8 | - | - | - | - | - |

| # | Alt | Res | High B | Clash > 0.4Å | Ramachandran | Rotamer | Cβ deviation | Bond lengths | Bond angles | Cis Peptides |
|---|---|---|---|---|---|---|---|---|---|---|
| A 58 | | VAL | 31.56 | - | Favored (27.4%) Ile or Val / -77.5,-39.3 | Favored (18.2%) *t* chi angles: 164.3 | 0.09Å | - | - | - |
| A 59 | | GLU | 36.09 | - | Favored (40.14%) General / -79.9,-33.0 | Favored (48.8%) *mm-30* chi angles: 295.4,303.9,293.6 | 0.03Å | - | - | - |
| A 60 | | ASN | 29.84 | - | Favored (3.48%) General / -112.4,91.0 | Favored (51.1%) *m-40* chi angles: 302.8,285.1 | 0.04Å | - | - | - |
| **#** | **Alt** | **Res** | **High B** | **Clash > 0.4Å** | **Ramachandran** | **Rotamer** | **Cβ deviation** | **Bond lengths** | **Bond angles** | **Cis Peptides** |
| | | | Avg: 34.13 | Clashscore: 2.93 | Outliers: 0 of 340 | Poor rotamers: 2 of 294 | Outliers: 0 of 323 | Outliers: 0 of 348 | Outliers: 1 of 348 | Non-Trans: 1 of 345 |
| A 61 | | LEU | 34.62 | - | Favored (61.62%) General / -71.2,-12.7 | Favored (31.5%) *mt* chi angles: 296.3,162.7 | 0.03Å | - | - | - |
| A 62 | | ASP | 29.43 | - | Favored (28.14%) General / -87.1,7.4 | Favored (24.9%) *p0* chi angles: 58.9,335 | 0.04Å | - | - | - |
| A 63 | | SER | 27.1 | - | Favored (48.5%) General / -57.8,141.5 | Favored (59.7%) *m* chi angles: 299.1 | 0.02Å | - | - | - |
| A 64 | | GLY | 26.54 | - | Favored (30.58%) Glycine / -76.1,-40.1 | - | - | - | - | - |
| A 65 | | VAL | 26.06 | - | Allowed (0.8%) Ile or Val / -105.7,-70.2 | Favored (89.4%) *t* chi angles: 176.1 | 0.09Å | - | - | - |
| A 66 | | GLY | 22.04 | - | Favored (55.33%) Glycine / 97.5,9.3 | - | - | - | - | - |
| A 67 | | ILE | 21.83 | - | Favored (25.35%) Ile or Val / -138.4,147.6 | Favored (6.6%) *tt* chi angles: 199.3,176.8 | 0.07Å | - | - | - |
| A 68 | | TYR | 26.17 | - | Favored (42.98%) General / -138.3,159.1 | Favored (89.2%) *m-80* chi angles: 290.9,273.3 | 0.07Å | - | - | - |
| A 69 | | ALA | 21.3 | - | Favored (46.35%) Pre-Pro / -98.8,124.3 | - | 0.03Å | - | - | - |
| A 70 | | PRO | 23.71 | - | Favored (3.58%) Trans-Pro / -84.8,-20.7 | Favored (65.2%) *Cg_endo* chi angles: 31.4,319.6,33.2 | 0.02Å | - | - | - |

| | | | | | | | | | | |
|---|---|---|---|---|---|---|---|---|---|---|
| A 71 | | ASP | 26.26 | - | Favored (11.41%) General / -159.3,177.1 | Favored (43.6%) *p0* chi angles: 62,22.1 | 0.05Å | - | - | - |
| A 72 | | ALA | 28.73 | - | Favored (77.64%) General / -69.2,-40.9 | - | 0.04Å | - | - | - |
| A 73 | | GLU | 29.74 | - | Favored (71.99%) General / -57.5,-36.9 | Favored (74.6%) *tt0* chi angles: 184.5,186.4,347.1 | 0.03Å | - | - | - |
| A 74 | | ALA | 24.48 | - | Favored (21.25%) General / -51.6,-30.5 | - | 0.02Å | - | - | - |
| A 75 | | TYR | 24.73 | - | Favored (94.93%) General / -64.9,-40.4 | Favored (9.3%) *m-80* chi angles: 272.3,299.8 | 0.02Å | - | - | - |
| A 76 | | THR | 24.31 | - | Favored (10%) General / -80.8,-50.3 | Favored (39.6%) *m* chi angles: 305.2 | 0.10Å | - | - | - |
| A 77 | | LEU | 27.21 | - | Favored (83.45%) General / -58.9,-40.1 | Favored (47.7%) *tp* chi angles: 173.9,65.7 | 0.03Å | - | - | - |
| A 78 | | PHE | 26.23 | - | Favored (5.32%) General / -100.7,28.2 | Favored (85.8%) *m-80* chi angles: 297.5,286.3 | 0.04Å | - | - | - |
| A 79 | | ALA | 24.82 | - | Favored (6.74%) Pre-Pro / -50.5,-32.7 | - | 0.04Å | - | - | - |
| A 80 | | PRO | 27.03 | - | Favored (86.13%) Trans-Pro / -61.1,-28.9 | Favored (55.2%) *Cg_exo* chi angles: 328.1,39,329.8 | 0.02Å | - | - | - |
| **#** | **Alt** | **Res** | **High B** | **Clash > 0.4Å** | **Ramachandran** | **Rotamer** | **Cβ deviation** | **Bond lengths** | **Bond angles** | **Cis Peptides** |
| | | | Avg: 34.13 | Clashscore: 2.93 | Outliers: 0 of 340 | Poor rotamers: 2 of 294 | Outliers: 0 of 323 | Outliers: 0 of 348 | Outliers: 1 of 348 | Non-Trans: 1 of 345 |
| A 81 | | ILE | 25.74 | - | Favored (4.82%) Ile or Val / -99.1,-35.3 | Favored (4.2%) *tp* chi angles: 181.5,56.1 | 0.06Å | - | - | - |
| A 82 | | PHE | 22.64 | - | Favored (79.56%) General / -66.0,-46.0 | Favored (14.2%) *m-80* chi angles: 275.2,299.6 | 0.02Å | - | - | - |
| A 83 | | ASN | 23.76 | - | Favored (96.43%) Pre-Pro / | Favored (63.8%) *m-40* chi angles: 280.6,353.1 | 0.06Å | - | - | - |

| | | | | | | | | | |
|---|---|---|---|---|---|---|---|---|---|
| | | | | -57.4,-49.0 | | | | | |
| A 84 | PRO | 22.33 | - | Favored (47.54%) Trans-Pro / -66.1,-30.1 | Favored (29.9%) *Cg_endo* chi angles: 21.3,328.7,28.4 | 0.03Å | - | - | - |
| A 85 | ILE | 24.36 | - | Favored (65.53%) Ile or Val / -72.3,-40.6 | Favored (49.3%) *mt* chi angles: 295.3,180.1 | 0.06Å | - | - | - |
| A 86 | ILE | 22.59 | - | Favored (85.58%) Ile or Val / -60.3,-49.1 | Favored (59.8%) *mt* chi angles: 286.9,166.2 | 0.07Å | - | - | - |
| A 87 | GLU | 27.94 | - | Favored (51.77%) General / -52.7,-53.1 | Favored (12.2%) *tm-30* chi angles: 190.8,287,318.5 | 0.04Å | - | - | - |
| A 88 | ASP | 25.84 | - | Favored (26.61%) General / -59.0,-56.1 | Favored (16.2%) *t0* chi angles: 195.4,316.1 | 0.04Å | - | - | - |
| A 89 | TYR | 28.28 | - | Favored (99.43%) General / -61.7,-42.8 | Favored (79.9%) *t80* chi angles: 182.2,85.3 | 0.05Å | - | - | - |
| A 90 | HIS | 24.21 | - | Favored (14.3%) General / -90.0,13.7 | Favored (89.1%) *m-70* chi angles: 294.7,274.2 | 0.08Å | - | - | - |
| A 91 | GLU | 34.06 | - | Favored (2.82%) General / 58.5,59.8 | Favored (64.6%) *mt-10* chi angles: 281,182.1,3.3 | 0.06Å | - | - | - |
| A 92 | GLY | 27.32 | - | Favored (10.38%) Glycine / 109.8,16.8 | - | - | - | - | - |
| A 93 | PHE | 22.42 | - | Favored (8.57%) General / -88.5,90.9 | Favored (38.3%) *t80* chi angles: 193.4,85.7 | 0.04Å | - | - | - |
| A 94 | LYS | 35.48 | - | Favored (80.57%) Pre-Pro / -70.8,160.9 | Favored (6.1%) *mmtm* chi angles: 319.9,299.4,175.8,320.6 | 0.03Å | - | - | - |
| A 95 | PRO | 26.18 | - | Favored (55.83%) Trans-Pro / -56.5,-25.1 | Favored (82.1%) *Cg_exo* chi angles: 334.5,30,338 | 0.03Å | - | - | - |
| A 96 | THR | 33.41 | - | Favored (49.72%) General / -94.2,-4.0 | Favored (14.4%) *p* chi angles: 47.6 | 0.03Å | - | - | - |
| A 97 | ASP | 24.78 | - | Favored (23.56%) General / -79.0,165.3 | Favored (35%) *m-30* chi angles: 276.7,338.2 | 0.01Å | - | - | - |

| | | | | | | | | | | |
|---|---|---|---|---|---|---|---|---|---|---|
| A 98 | | LYS | 30.46 | - | Favored (29.74%) General / -141.1,134.5 | Favored (75.9%) *tttt* chi angles: 170.7,178.5,181.9,176.8 | 0.03Å | - | - | - |
| A 99 | | HIS | 21.01 | - | Favored (43.26%) Pre-Pro / -83.6,142.8 | Favored (82.6%) *t70* chi angles: 178.4,79.8 | 0.06Å | - | - | - |
| A 100 | | PRO | 21.45 | - | Favored (46.72%) Trans-Pro / -66.8,160.4 | Favored (35%) *Cg_endo* chi angles: 22.3,323.2,36.7 | 0.04Å | - | - | - |
| **#** | **Alt** | **Res** | **High B** | **Clash > 0.4Å** | **Ramachandran** | **Rotamer** | **Cβ deviation** | **Bond lengths** | **Bond angles** | **Cis Peptides** |
| | | | Avg: 34.13 | Clashscore: 2.93 | Outliers: 0 of 340 | Poor rotamers: 2 of 294 | Outliers: 0 of 323 | Outliers: 0 of 348 | Outliers: 1 of 348 | Non-Trans: 1 of 345 |
| A 101 | | PRO | 23.51 | - | Favored (41.16%) Trans-Pro / -63.3,159.1 | Favored (3%) *Cg_exo* chi angles: 354.7,17.7,337.3 | 0.05Å | - | - | - |
| A 102 | | THR | 24.09 | - | Favored (55.61%) General / -58.0,133.5 | Favored (13.7%) *m* chi angles: 311.1 | 0.05Å | - | - | - |
| A 103 | | ASP | 27.25 | - | Favored (3.03%) General / -155.3,110.6 | Favored (42%) *t0* chi angles: 186.9,12.5 | 0.03Å | - | - | - |
| A 104 | | PHE | 30.83 | - | Favored (56.07%) General / -83.0,-13.4 | Favored (71.9%) *m-80* chi angles: 300.7,289.8 | 0.03Å | - | - | - |
| A 105 | | GLY | 31.92 | - | Favored (53.74%) Glycine / 77.9,-167.7 | - | - | - | - | - |
| A 106 | | ASP | 36.32 | - | Favored (11.16%) General / -87.6,98.0 | Favored (41.6%) *t0* chi angles: 190.7,2.8 | 0.05Å | - | - | - |
| A 107 | | ILE | 33.02 | - | Favored (35.67%) Ile or Val / -60.7,-25.7 | Favored (3.1%) *tp* chi angles: 185.2,51.8 | 0.08Å | - | - | - |
| A 108 | | ASN | 38.05 | - | Favored (59%) General / -77.3,-9.8 | Favored (10.2%) *m-40* chi angles: 275.5,262.8 | 0.04Å | - | - | - |
| A 109 | | THR | 33.28 | - | Favored (58.02%) General / -84.3,-3.1 | Favored (16.9%) *p* chi angles: 73.9 | 0.03Å | - | - | - |
| A 110 | | ILE | 29.73 | - | Favored (15.89%) Ile or Val / -83.9,140.6 | Favored (45.6%) *mm* chi angles: 298,299.4 | 0.05Å | - | - | - |

| # | Alt | Res | High B | Clash > 0.4Å | Ramachandran | Rotamer | Cβ deviation | Bond lengths | Bond angles | Cis Peptides |
|---|---|---|---|---|---|---|---|---|---|---|
| A 111 | | VAL | 31.07 | - | Favored (21.98%) Ile or Val / -125.9,166.4 | Favored (3.4%) *m* chi angles: 309.6 | 0.04Å | - | - | - |
| A 112 | | ASN | 31.67 | - | Favored (24.09%) General / -73.2,123.7 | Favored (38.3%) *t0* chi angles: 188.9,310.8 | 0.04Å | - | - | - |
| A 113 | | VAL | 27.75 | - | Favored (14.23%) Ile or Val / -77.6,-14.2 | Favored (6.4%) *m* chi angles: 288.4 | 0.05Å | - | - | - |
| A 114 | | ASP | 28.88 | - | Favored (5.11%) Pre-Pro / -156.0,73.8 | Favored (25.2%) *t0* chi angles: 179.2,27.4 | 0.03Å | - | - | - |
| A 115 | | PRO | 35.7 | - | Favored (43.02%) Trans-Pro / -58.9,-18.6 | Favored (7.8%) *Cg_exo* chi angles: 348.5,26.3,329.9 | 0.03Å | - | - | - |
| A 116 | | SER | 34.05 | - | Favored (68.93%) General / -68.8,-30.4 | Favored (6.8%) *p* chi angles: 84 | 0.01Å | - | - | - |
| A 117 | | GLY | 30.05 | - | Favored (61.65%) Glycine / 74.2,2.0 | - | - | - | - | - |
| A 118 | | LYS | 32.39 | - | Favored (41.11%) General / -80.3,-23.0 | Favored (36.5%) *mtmt* chi angles: 302.3,199.4,285.1,180.1 | 0.04Å | - | - | - |
| A 119 | | TYR | 27.77 | - | Allowed (1.76%) General / -124.1,-52.3 | Favored (66.5%) *m-80* chi angles: 303.8,287.9 | 0.04Å | - | - | - |
| A 120 | | VAL | 25.09 | - | Favored (32.77%) Ile or Val / -86.2,118.5 | Favored (94.7%) *t* chi angles: 174.8 | 0.03Å | - | - | - |
| | | | Avg: 34.13 | Clashscore: 2.93 | Outliers: 0 of 340 | Poor rotamers: 2 of 294 | Outliers: 0 of 323 | Outliers: 0 of 348 | Outliers: 1 of 348 | Non-Trans: 1 of 345 |
| A 121 | | VAL | 27.64 | - | Favored (26.97%) Ile or Val / -75.7,-49.3 | Favored (84.2%) *t* chi angles: 173.4 | 0.07Å | - | - | - |
| A 122 | | SER | 24.65 | - | Favored (35.96%) General / -151.2,154.0 | Favored (7.6%) *p* chi angles: 49.4 | 0.05Å | - | - | - |
| A 123 | | THR | 24.23 | - | Favored (47.48%) | Favored (88.4%) *m* chi angles: 301.3 | 0.04Å | - | - | - |

| | | | | | | | | | |
|---|---|---|---|---|---|---|---|---|---|
| | | | General / -122.7,146.4 | | | | | | |
| A 124 | HIS | 26.21 | - | Favored (25.83%) General / -146.6,140.1 | Allowed (1.3%) *m-70* chi angles: 323.6,260.5 | 0.07Å | - | - | - |
| A 125 | VAL | 24.51 | - | Favored (33.97%) Ile or Val / -131.0,116.5 | Favored (32.7%) *t* chi angles: 185.5 | 0.06Å | - | - | - |
| A 126 | ARG | 30 | - | Favored (46.52%) General / -109.4,137.9 | Favored (26.4%) *ttm170* chi angles: 173.7,197.9,296.1,172.2 | 0.06Å | - | - | - |
| A 127 | CYS | 25.49 | - | Favored (15.38%) General / -135.7,170.8 | Favored (11.4%) *p* chi angles: 74.5 | 0.04Å | - | - | - |
| A 128 | GLY | 20.71 | - | Favored (21.29%) Glycine / -135.1,153.8 | - | - | - | - | - |
| A 129 | ARG | 30 | - | Favored (23.31%) General / -147.4,137.8 | Favored (86.5%) *mmt-90* chi angles: 294.2,286.8,189.4,267.3 | 0.06Å | - | - | - |
| A 130 | SER | 21.3 | - | Favored (30.16%) General / -99.0,142.7 | Favored (63.3%) *m* chi angles: 297.8 | 0.02Å | - | - | - |
| A 131 | LEU | 27.18 | - | Favored (35.26%) General / -90.0,132.7 | Favored (33.3%) *mt* chi angles: 306.4,171.8 | 0.01Å | - | - | - |
| A 132 | LYS | 34.18 | 0.67Å NZ with S 213 HOH O | Favored (57.93%) General / -62.3,136.4 | Allowed (1.5%) *tttt* chi angles: 186.7,142.7,147.1,215.2 | 0.01Å | - | - | - |
| A 133 | GLY | 28.12 | - | Favored (64.46%) Glycine / 96.5,-14.3 | - | - | - | - | - |
| A 134 | TYR | 34.53 | - | Favored (22.58%) Pre-Pro / -102.7,141.5 | Favored (77.4%) *m-80* chi angles: 288.3,274 | 0.04Å | - | - | - |
| A 135 | PRO | 29.35 | - | Favored (10.76%) Trans-Pro / -65.2,170.3 | Allowed (1%) *Cg_exo* chi angles: 359.5,13.5,339.4 | 0.02Å | - | - | - |
| A 136 | PHE | 24.47 | - | Favored (7.27%) General / -89.1,175.7 | Favored (10.1%) *m-10* chi angles: 297.2,353.7 | 0.06Å | - | - | - |

| # | Alt | Res | High B | Clash > 0.4Å | Ramachandran | Rotamer | Cβ deviation | Bond lengths | Bond angles | Cis Peptides |
|---|---|---|---|---|---|---|---|---|---|---|
| A 137 | | ASN | 25.47 | 0.60Å HA with A 140 LEU HD12 | Favored (7.58%) Pre-Pro / -55.1,-30.6 | Favored (9.5%) *m-40* chi angles: 268.6,275.4 | 0.08Å | - | - | - |
| A 138 | | PRO | 26.17 | - | Favored (68.73%) Trans-Pro / -61.4,-20.5 | Favored (85.5%) *Cg_exo* chi angles: 333.9,35.6,329.4 | 0.03Å | - | - | - |
| A 139 | | CYS | 27.99 | - | Favored (5%) General / -131.4,12.7 | Favored (93.1%) *m* chi angles: 291.8 | 0.01Å | - | - | - |
| A 140 | | LEU | 26.81 | 0.60Å HD12 with A 137 ASN HA | Favored (41.72%) General / -76.1,142.2 | Favored (17.8%) *mt* chi angles: 286.1,157.5 | 0.05Å | - | - | - |
| **#** | **Alt** | **Res** | **High B** | **Clash > 0.4Å** | **Ramachandran** | **Rotamer** | **Cβ deviation** | **Bond lengths** | **Bond angles** | **Cis Peptides** |
| | | | Avg: 34.13 | Clashscore: 2.93 | Outliers: 0 of 340 | Poor rotamers: 2 of 294 | Outliers: 0 of 323 | Outliers: 0 of 348 | Outliers: 1 of 348 | Non-Trans: 1 of 345 |
| A 141 | | THR | 32.2 | - | Favored (13.43%) General / -91.2,165.5 | Favored (60.2%) *p* chi angles: 64 | 0.07Å | - | - | - |
| A 142 | | GLU | 33.44 | - | Favored (72.36%) General / -54.4,-44.6 | Favored (4.5%) *tt0* chi angles: 192.9,147.8,30.1 | 0.03Å | - | - | - |
| A 143 | | ALA | 28.9 | - | Favored (99.06%) General / -63.3,-42.4 | - | 0.01Å | - | - | - |
| A 144 | | ASN | 27.56 | - | Favored (8.74%) General / -52.7,-57.9 | Favored (7.3%) *m110* chi angles: 290.6,153.3 | 0.06Å | - | - | - |
| A 145 | | TYR | 25.75 | - | Favored (36.23%) General / -48.8,-50.1 | Favored (26.3%) *m-80* chi angles: 278.3,289.8 | 0.02Å | - | - | - |
| A 146 | | LYS | 43.7 | - | Favored (75%) General / -58.7,-50.1 | Favored (69.7%) *mttt* chi angles: 277.7,188,180.4,172.7 | 0.02Å | - | - | - |
| A 147 | | GLU | 37.46 | 0.64Å OE1 with S 77 HOH O | Favored (97.17%) General / -64.0,-42.4 | Favored (14.7%) *tt0* chi angles: 168.6,198.6,358.7 | 0.03Å | - | - | - |
| A 148 | | MET | 26.65 | - | Favored (97.5%) General / -61.0,-44.4 | Favored (41.8%) *ttm* chi angles: 184.7,189,298.5 | 0.02Å | - | - | - |
| A 149 | | GLU | 29.3 | - | Favored (88.28%) General / | Favored (59.3%) *tt0* chi angles: | 0.02Å | - | - | - |

| # | Alt | Res | High B | Clash > 0.4Å | Ramachandran | Rotamer | Cβ deviation | Bond lengths | Bond angles | Cis Peptides |
|---|---|---|---|---|---|---|---|---|---|---|
| | | | | | -61.8,-38.5 | 179.4,183,26.1 | | | | |
| A 150 | | ASP | 34.89 | - | Favored (99.05%) General / -61.6,-42.5 | Favored (36.7%) *t0* chi angles: 193.6,7.4 | 0.05Å | - | - | - |
| A 151 | | LYS | 33.94 | - | Favored (68.59%) General / -71.5,-42.1 | Favored (12.7%) *mttm* chi angles: 300.2,176.8,180.5,326.4 | 0.03Å | - | - | - |
| A 152 | | VAL | 29.35 | - | Favored (82.4%) Ile or Val / -66.7,-47.7 | Allowed (1.8%) *t* chi angles: 158.1 | 0.10Å | - | - | - |
| A 153 | | SER | 31.43 | - | Favored (81.89%) General / -61.7,-36.8 | Favored (65.4%) *p* chi angles: 58.3 | 0.03Å | - | - | - |
| A 154 | | ALA | 31.59 | - | Favored (77.72%) General / -56.0,-47.5 | - | 0.04Å | - | - | - |
| A 155 | | ILE | 31.37 | - | Favored (96.35%) Ile or Val / -61.6,-46.5 | Favored (70.1%) *mt* chi angles: 292.3,160.7 | 0.05Å | - | - | - |
| A 156 | | PHE | 30.05 | - | Favored (67.68%) General / -56.7,-34.7 | Favored (22.6%) *m-10* chi angles: 294.2,344.5 | 0.02Å | - | - | - |
| A 157 | | GLY | 34.44 | - | Favored (60.69%) Glycine / -59.6,-25.1 | - | - | - | - | - |
| A 158 | | THR | 34.53 | - | Favored (56.27%) General / -85.7,-10.1 | Favored (24.8%) *p* chi angles: 71 | 0.02Å | - | - | - |
| A 159 | | PHE | 32.37 | 0.41Å O with A 164 LYS HD3 | Favored (46.44%) General / -58.4,130.4 | Favored (7.5%) *m-10* chi angles: 282.1,350.2 | 0.04Å | - | - | - |
| A 160 | | GLU | 43.26 | - | Favored (5.14%) General / -126.4,179.8 | Allowed (0.6%) *mp0* chi angles: 324.9,108.8,302.1 | 0.03Å | - | - | - |
| **#** | **Alt** | **Res** | **High B** | **Clash > 0.4Å** | **Ramachandran** | **Rotamer** | **Cβ deviation** | **Bond lengths** | **Bond angles** | **Cis Peptides** |
| | | | Avg: 34.13 | Clashscore: 2.93 | Outliers: 0 of 340 | Poor rotamers: 2 of 294 | Outliers: 0 of 323 | Outliers: 0 of 348 | Outliers: 1 of 348 | Non-Trans: 1 of 345 |
| A 161 | | GLY | 32.73 | - | Favored (36.66%) Glycine / 71.7,-144.3 | - | - | - | - | - |

| | | | | | | | | | | |
|---|---|---|---|---|---|---|---|---|---|---|
| A 162 | GLU | 32.22 | 0.72Å OE1 with S 237 HOH O | Favored (64.55%) General / -66.8,-16.9 | Favored (12.5%) *pt0* chi angles: 72.3,172.8,160.9 | 0.01Å | - | - | - | |
| A 163 | LEU | 29.75 | - | Favored (53.99%) General / -85.6,-11.4 | Favored (14.8%) *mt* chi angles: 290.6,189.4 | 0.04Å | - | - | - | |
| A 164 | LYS | 30.76 | 0.41Å HD3 with A 159 PHE O | Favored (44.33%) General / -66.1,130.8 | Favored (2.2%) *tptp* chi angles: 185.5,36.2,189.4,52.1 | 0.03Å | - | - | - | |
| A 165 | GLY | 27.82 | - | Favored (3.89%) Glycine / -164.2,-144.0 | - | - | - | - | - | |
| A 166 | LYS | 28.59 | - | Favored (36.9%) General / -139.6,139.8 | Favored (38.6%) *tttm* chi angles: 193.4,168.8,191.4,297.2 | 0.03Å | - | - | - | |
| A 167 | TYR | 28.31 | - | Favored (37.69%) General / -95.0,134.5 | Favored (70.2%) *t80* chi angles: 180.3,68.2 | 0.01Å | - | - | - | |
| A 168 | TYR | 27.85 | - | Favored (52.26%) Pre-Pro / -116.2,104.2 | Favored (34.6%) *m-80* chi angles: 305.4,80.7 | 0.03Å | - | - | - | |
| A 169 | PRO | 27.17 | - | Favored (63.96%) Trans-Pro / -64.8,139.7 | Allowed (1.7%) *Cg_exo* chi angles: 358.3,9.5,346.8 | 0.04Å | - | - | - | |
| A 170 | LEU | 26.2 | - | Favored (66.64%) General / -70.1,-29.6 | Favored (2.5%) *mp* chi angles: 259.7,54.3 | 0.05Å | - | - | - | |
| A 171 | THR | 25.77 | - | Favored (15.12%) General / -67.2,121.2 | Favored (95.3%) *m* chi angles: 300.8 | 0.03Å | - | - | - | |
| A 172 | GLY | 25.06 | - | Favored (79.48%) Glycine / 74.0,17.5 | - | - | - | - | - | |
| A 173 | MET | 23.43 | - | Favored (41.6%) General / -72.7,130.9 | Favored (59.9%) *ttm* chi angles: 183.5,175.3,287.7 | 0.05Å | - | - | - | |
| A 174 | ASP | 24.47 | - | Favored (19.02%) General / -87.0,159.4 | Favored (2.8%) *p0* chi angles: 66.8,71.9 | 0.04Å | - | - | - | |
| A 175 | LYS | 28.89 | - | Favored (69.94%) General / -63.6,-28.4 | Favored (4.7%) *tttp* chi angles: 212.7,176.6,169.7,89.4 | 0.03Å | - | - | - | |

| # | Alt | Res | High B | Clash > 0.4Å | Ramachandran | Rotamer | Cβ deviation | Bond lengths | Bond angles | Cis Peptides |
|---|---|---|---|---|---|---|---|---|---|---|
| A 176 | | ALA | 24.84 | - | Favored (63.51%) General / -72.2,-44.1 | - | 0.03Å | - | - | - |
| A 177 | | THR | 24.8 | - | Favored (77.62%) General / -56.3,-47.9 | Favored (99.5%) *m* chi angles: 300.5 | 0.07Å | - | - | - |
| A 178 | | GLN | 28.55 | - | Favored (74.14%) General / -54.8,-47.4 | Favored (80.1%) *mt0* chi angles: 283,174.8,347.9 | 0.02Å | - | - | - |
| A 179 | | GLN | 31.95 | - | Favored (91.28%) General / -63.6,-38.5 | Favored (85.9%) *tp40* chi angles: 186.6,61,43.5 | 0.01Å | - | - | - |
| A 180 | | GLN | 35.19 | - | Favored (80.89%) General / -57.2,-42.4 | Favored (36.5%) *tt0* chi angles: 172.8,186.4,336.2 | 0.07Å | - | - | - |
| **#** | **Alt** | **Res** | **High B** | **Clash > 0.4Å** | **Ramachandran** | **Rotamer** | **Cβ deviation** | **Bond lengths** | **Bond angles** | **Cis Peptides** |
| | | | Avg: 34.13 | Clashscore: 2.93 | Outliers: 0 of 340 | Poor rotamers: 2 of 294 | Outliers: 0 of 323 | Outliers: 0 of 348 | Outliers: 1 of 348 | Non-Trans: 1 of 345 |
| A 181 | | LEU | 25.24 | - | Favored (54.44%) General / -77.4,-34.9 | Favored (96.3%) *mt* chi angles: 291.9,172.4 | 0.06Å | - | - | - |
| A 182 | | ILE | 29.21 | - | Favored (85.72%) Ile or Val / -57.8,-44.2 | Favored (70.5%) *mt* chi angles: 292.8,160.7 | 0.07Å | - | - | - |
| A 183 | | ASP | 33.87 | - | Favored (69.42%) General / -60.5,-30.0 | Favored (83.2%) *m-30* chi angles: 289.9,333.6 | 0.02Å | - | - | - |
| A 184 | | ASP | 30.13 | - | Favored (59.19%) General / -86.6,-5.3 | Favored (73.1%) *m-30* chi angles: 296.4,316.2 | 0.02Å | - | - | - |
| A 185 | | HIS | 30.54 | - | Favored (18.9%) General / 60.5,29.3 | Favored (65.7%) *m-70* chi angles: 302.1,265.3 | 0.04Å | - | - | - |
| A 186 | | PHE | 26.33 | - | Favored (29.31%) General / -102.8,-1.7 | Favored (20.9%) *m-10* chi angles: 291.3,345.6 | 0.04Å | - | - | - |
| A 187 | | LEU | 24.96 | - | Favored (31.48%) General / -94.5,139.2 | Favored (10.8%) *tp* chi angles: 189.5,76.8 | 0.03Å | - | - | - |
| A 188 | | PHE | 23.45 | - | Favored (26.75%) General / | Favored (55.7%) *p90* chi angles: 65.3,270 | 0.06Å | - | - | - |

| | | | | | | | | | | |
|---|---|---|---|---|---|---|---|---|---|---|
| | | | | | -118.3,157.6 | | | | | |
| A 189 | | LYS | 37.97 | - | Favored (4.74%) General / -122.3,178.9 | Favored (4.2%) *ptpp* chi angles: 66,171.2,89,56 | 0.01Å | - | - | - |
| A 190 | | GLU | 34.21 | - | Favored (47.13%) General / -60.6,146.0 | Favored (12.3%) *tt0* chi angles: 207.1,180.7,344.6 | 0.01Å | - | - | - |
| A 191 | | GLY | 28.29 | - | Favored (46.17%) Glycine / -66.0,159.8 | - | - | - | - | - |
| A 192 | | ASP | 25.23 | - | Favored (2.21%) General / -78.2,-171.0 | Favored (52%) *p0* chi angles: 62.9,14.7 | 0.06Å | - | - | - |
| A 193 | | ARG | 30 | - | Favored (66.66%) General / -67.0,-26.1 | Favored (20.7%) *ptt90* chi angles: 67.1,193.3,159.3,91.3 | 0.03Å | - | - | - |
| A 194 | | PHE | 26.67 | - | Favored (79.18%) General / -66.7,-45.3 | Favored (93%) *m-80* chi angles: 296.7,282.4 | 0.01Å | - | - | - |
| A 195 | | LEU | 26.12 | - | Favored (73.32%) General / -70.8,-37.8 | Favored (34.9%) *mt* chi angles: 292.8,185 | 0.03Å | - | - | - |
| A 196 | | GLN | 26.98 | - | Favored (78.94%) General / -60.7,-49.1 | Favored (69.3%) *mm-40* chi angles: 297.8,286.9,330.1 | 0.03Å | - | - | - |
| A 197 | | ALA | 26.25 | - | Favored (63.92%) General / -64.6,-17.4 | - | 0.07Å | - | - | - |
| A 198 | | ALA | 23.52 | - | Favored (56.61%) General / -86.8,-0.5 | - | 0.05Å | - | - | - |
| A 199 | | ASN | 28.07 | - | Favored (17.66%) General / 62.2,31.5 | Favored (49.4%) *m-40* chi angles: 276.2,341.3 | 0.05Å | - | - | - |
| A 200 | | ALA | 24.5 | - | Favored (47.43%) General / -88.8,-10.7 | - | 0.05Å | - | - | - |
| **#** | **Alt** | **Res** | **High B** | **Clash > 0.4Å** | **Ramachandran** | **Rotamer** | **Cβ deviation** | **Bond lengths** | **Bond angles** | **Cis Peptides** |
| | | | Avg: 34.13 | Clashscore: 2.93 | Outliers: 0 of 340 | Poor rotamers: 2 of 294 | Outliers: 0 of 323 | Outliers: 0 of 348 | Outliers: 1 of 348 | Non-Trans: 1 of 345 |

| | | | | | | | | | | |
|---|---|---|---|---|---|---|---|---|---|---|
| A 201 | CYS | 29.26 | - | Favored (13.98%) General / -116.9,20.2 | Favored (34.5%) *m* chi angles: 306 | 0.04Å | - | - | - |
| A 202 | ARG | 30 | - | Favored (56.05%) General / -61.9,143.7 | Favored (36.9%) *mtt180* chi angles: 288.8,159.2,154.9,170.2 | 0.09Å | - | - | - |
| A 203 | TYR | 33.41 | - | Favored (17.66%) General / 62.0,29.2 | Favored (3.7%) *m-80* chi angles: 322,291.8 | 0.05Å | - | - | - |
| A 204 | TRP | 27.82 | - | Favored (96.01%) Pre-Pro / -62.5,136.8 | Favored (28.7%) *t-100* chi angles: 196.9,269 | 0.04Å | - | - | - |
| A 205 | PRO | 26.04 | - | Favored (15.35%) Cis-Pro / -101.5,25.5 | Favored (49.5%) *Cg_endo* chi angles: 33.1,335.6,5.9 | 0.03Å | - | - | Cis PRO omega= 2.36 |
| A 206 | THR | 25.1 | - | Favored (39.21%) General / -61.7,129.1 | Favored (96.5%) *m* chi angles: 300.8 | 0.06Å | - | - | - |
| A 207 | GLY | 25.57 | - | Favored (83.49%) Glycine / 80.3,-0.8 | - | - | - | - | - |
| A 208 | ARG | 30 | - | Favored (33.64%) General / -100.5,141.2 | Favored (5.5%) *mpt180* chi angles: 282.5,70.9,163,203 | 0.06Å | - | - | - |
| A 209 | GLY | 23.13 | - | Favored (42.38%) Glycine / -170.8,169.4 | - | - | - | - | - |
| A 210 | ILE | 23.97 | - | Favored (67.91%) Ile or Val / -128.0,133.1 | Favored (8%) *mt* chi angles: 297.7,148.3 | 0.03Å | - | - | - |
| A 211 | TYR | 26.64 | - | Favored (49.78%) General / -122.3,143.2 | Favored (63.2%) *t80* chi angles: 181.9,272.1 | 0.01Å | - | - | - |
| A 212 | HIS | 26.91 | - | Favored (26.7%) General / -153.3,148.4 | Favored (8%) *p90* chi angles: 77.1,72.5 | 0.08Å | - | - | - |
| A 213 | ASN | 26.3 | - | Favored (21.82%) General / -82.8,162.1 | Favored (40.2%) *p0* chi angles: 69.8,341.2 | 0.07Å | - | - | - |
| A 214 | ASP | 30.75 | - | Favored (73.74%) General / -58.5,-36.4 | Favored (81%) *m-30* chi angles: 295,332.6 | 0.03Å | - | - | - |

| | | | | | | | | | | |
|---|---|---|---|---|---|---|---|---|---|---|
| A 215 | | ALA | 25.9 | - | Favored (56.41%) General / -70.7,-9.1 | - | 0.02Å | - | - | - |
| A 216 | | LYS | 30.76 | - | Favored (29.74%) General / 53.8,39.0 | Favored (93.4%) *mttt* chi angles: 297.1,176.8,182.2,172 | 0.05Å | - | - | - |
| A 217 | | THR | 24.04 | - | Favored (17.23%) General / -114.4,11.4 | Favored (33.7%) *p* chi angles: 53.8 | 0.04Å | - | - | - |
| A 218 | | PHE | 25.34 | - | Favored (12.75%) General / -151.5,130.7 | Favored (82%) *t80* chi angles: 176.8,71.9 | 0.03Å | - | - | - |
| A 219 | | LEU | 23.52 | - | Favored (29.48%) General / -143.7,139.3 | Favored (2.5%) *tp* chi angles: 169.5,83.6 | 0.02Å | - | - | - |
| A 220 | | VAL | 22.35 | 0.41Å HA with A 229 ARG O | Favored (67.23%) Ile or Val / -129.5,131.4 | Favored (58.3%) *t* chi angles: 180.2 | 0.02Å | - | - | - |
| **#** | **Alt** | **Res** | **High B** | **Clash > 0.4Å** | **Ramachandran** | **Rotamer** | **Cβ deviation** | **Bond lengths** | **Bond angles** | **Cis Peptides** |
| | | | Avg: 34.13 | Clashscore: 2.93 | Outliers: 0 of 340 | Poor rotamers: 2 of 294 | Outliers: 0 of 323 | Outliers: 0 of 348 | Outliers: 1 of 348 | Non-Trans: 1 of 345 |
| A 221 | | TRP | 23.44 | - | Favored (52.44%) General / -104.6,127.8 | Favored (46.8%) *m-10* chi angles: 287.5,356.1 | 0.02Å | - | - | - |
| A 222 | | VAL | 20.89 | - | Favored (43.22%) Ile or Val / -102.5,114.9 | Favored (36.6%) *t* chi angles: 184.2 | 0.03Å | - | - | - |
| A 223 | | ASN | 21.25 | - | Favored (7.97%) General / 65.6,34.2 | Favored (62%) *m-40* chi angles: 283.6,313.5 | 0.11Å | - | - | - |
| A 224 | | GLU | 25.14 | - | Favored (30.47%) General / -78.7,-41.8 | Favored (9.4%) *tm-30* chi angles: 177.2,267,335.6 | 0.03Å | - | - | - |
| A 225 | | GLU | 27.26 | 0.41Å HG3 with A 270 PHE HD2 | Allowed (0.24%) General / -146.3,-53.1 | Favored (6.9%) *tt0* chi angles: 163.4,168.9,296.5 | 0.04Å | - | - | - |
| A 226 | | ASP | 21.9 | - | Favored (11.67%) General / -97.2,166.2 | Favored (13.5%) *m-30* chi angles: 281.1,306.1 | 0.01Å | - | - | - |
| A 227 | | HIS | 21.52 | - | Favored (62.28%) General / | Favored (53.8%) *m170* chi angles: 297.1,159.7 | 0.02Å | - | - | - |

| | | | | | | | | | | |
|---|---|---|---|---|---|---|---|---|---|---|
| | | | | | -51.3,-48.7 | | | | | |
| A 228 | | LEU | 23.53 | - | Favored (30.61%) General / -132.7,125.6 | Favored (95.7%) *mt* chi angles: 296.9,176 | 0.08Å | - | - | - |
| A 229 | | ARG | 30 | 0.41Å O with A 220 VAL HA | Favored (45.28%) General / -111.3,121.5 | Favored (3.7%) *ttm-80* chi angles: 173.4,199.5,293.3,232.4 | 0.02Å | - | - | - |
| A 230 | | ILE | 24.45 | - | Favored (59.06%) Ile or Val / -119.1,118.9 | Favored (17.6%) *mt* chi angles: 311.2,165.3 | 0.06Å | - | - | - |
| A 231 | | ILE | 27.58 | - | Favored (63.08%) Ile or Val / -124.1,135.6 | Allowed (1.8%) *mt* chi angles: 312.4,194.6 | 0.04Å | - | - | - |
| A 232 | | SER | 24.17 | - | Favored (54.46%) General / -118.5,135.3 | Favored (38.2%) *t* chi angles: 177.3 | 0.01Å | - | - | - |
| A 233 | | MET | 31.71 | - | Favored (26.48%) General / -162.6,164.8 | Allowed (0.3%) *pmt* chi angles: 68.7,292,176 | 0.03Å | - | - | - |
| A 234 | | GLN | 29.75 | 0.59Å HG2 with A 242 ILE HD11 | Favored (2.16%) General / -177.4,165.1 | Favored (18.7%) *pt0* chi angles: 62.6,188.1,51.5 | 0.06Å | - | - | - |
| A 235 | | LYS | 32.92 | - | Favored (49.96%) General / -67.8,149.3 | Favored (4.4%) *mmtt* chi angles: 300.1,265.7,215.6,188.5 | 0.04Å | - | - | - |
| A 236 | | GLY | 24.57 | - | Favored (27.68%) Glycine / 89.5,-158.6 | - | - | - | - | - |
| A 237 | | GLY | 27.21 | - | Favored (4.21%) Glycine / -129.3,27.5 | - | - | - | - | - |
| A 238 | | ASP | 33.45 | - | Favored (7.01%) General / -80.3,71.0 | Favored (53%) *t0* chi angles: 187.5,0.9 | 0.02Å | - | - | - |
| A 239 | | LEU | 27.27 | - | Favored (66.8%) General / -57.9,-31.6 | Favored (41.8%) *tp* chi angles: 185.4,62.7 | 0.04Å | - | - | - |
| A 240 | | LYS | 35.53 | - | Favored (17.83%) General / -62.8,-56.4 | Favored (54.7%) *tttt* chi angles: 181.7,166.3,162.8,186.9 | 0.01Å | - | - | - |
| **#** | **Alt** | **Res** | **High B** | **Clash > 0.4Å** | **Ramachandran** | **Rotamer** | **Cβ deviation** | **Bond lengths** | **Bond angles** | **Cis Peptides** |

| | | | | | | | | | |
|---|---|---|---|---|---|---|---|---|---|
| | | Avg: 34.13 | Clashscore: 2.93 | Outliers: 0 of 340 | Poor rotamers: 2 of 294 | Outliers: 0 of 323 | Outliers: 0 of 348 | Outliers: 1 of 348 | Non-Trans: 1 of 345 |
| A 241 | THR | 29.42 | - | Favored (75.1%) General / -57.9,-38.1 | Favored (44.6%) *m* chi angles: 304.6 | 0.05Å | - | - | - |
| A 242 | ILE | 24.55 | 0.59Å HD11 with A 234 GLN HG2 | Favored (70.6%) Ile or Val / -67.3,-49.6 | Favored (71%) *mt* chi angles: 289.8,163.1 | 0.07Å | - | - | - |
| A 243 | PHE | 24.46 | - | Favored (90.13%) General / -65.4,-38.5 | Favored (7.7%) *t80* chi angles: 186.3,34.8 | 0.06Å | - | - | - |
| A 244 | GLN | 32.81 | - | Favored (75.65%) General / -67.7,-33.8 | Favored (51.5%) *tp40* chi angles: 181.9,56.6,70.2 | 0.01Å | - | - | - |
| A 245 | ARG | 30 | - | Favored (92.9%) General / -62.5,-45.5 | Favored (15.4%) *ttp-110* chi angles: 186.9,159.3,49.1,235.9 | 0.03Å | - | - | - |
| A 246 | LEU | 27.25 | - | Favored (85.88%) General / -58.9,-47.1 | Favored (45.5%) *tp* chi angles: 184.4,63.4 | 0.03Å | - | - | - |
| A 247 | VAL | 30 | - | Favored (85.7%) Ile or Val / -63.1,-48.9 | Favored (70.3%) *t* chi angles: 172.1 | 0.05Å | - | - | - |
| A 248 | ASN | 32.67 | - | Favored (61.43%) General / -51.1,-44.9 | Favored (92.8%) *m-40* chi angles: 290.7,346.2 | 0.04Å | - | - | - |
| A 249 | ALA | 27.17 | - | Favored (63.41%) General / -66.4,-50.4 | - | 0.01Å | - | - | - |
| A 250 | VAL | 26.35 | - | Favored (92.26%) Ile or Val / -59.0,-44.8 | Favored (89.3%) *t* chi angles: 174.1 | 0.08Å | - | - | - |
| A 251 | ASN | 32.2 | - | Favored (94.1%) General / -60.4,-41.6 | Favored (82.5%) *m-40* chi angles: 283.1,345.1 | 0.02Å | - | - | - |
| A 252 | THR | 31.5 | - | Favored (90.56%) General / -60.9,-46.5 | Favored (99.3%) *m* chi angles: 300.3 | 0.08Å | - | - | - |
| A 253 | ILE | 25.98 | - | Favored (87.47%) Ile or Val / -61.2,-48.8 | Favored (31.2%) *mt* chi angles: 290,155.5 | 0.08Å | - | - | - |

| # | Alt | Res | High B | Clash > 0.4Å | Ramachandran | Rotamer | Cβ deviation | Bond lengths | Bond angles | Cis Peptides |
|---|---|---|---|---|---|---|---|---|---|---|
| A 254 | | GLU | 28.7 | - | Favored (70.48%) General / -58.1,-34.7 | Favored (57.1%) *tt0* chi angles: 186.4,180.4,27.9 | 0.03Å | - | - | - |
| A 255 | | SER | 32 | - | Favored (61.92%) General / -58.4,-25.4 | Favored (8.3%) *p* chi angles: 83.2 | 0.02Å | - | - | - |
| A 256 | | LYS | 36.72 | 0.40Å HA with A 256 LYS HD3 | Favored (9.18%) General / -106.3,-30.4 | Favored (6.3%) *mmtp* chi angles: 295.8,311.5,154.3,67.3 | 0.03Å | - | - | - |
| A 257 | | LEU | 30.57 | - | Favored (15.88%) Pre-Pro / -131.8,116.4 | Allowed (1.1%) *mt* chi angles: 273.3,200.2 | 0.01Å | - | - | - |
| A 258 | | PRO | 29.7 | - | Favored (49.32%) Trans-Pro / -72.5,149.0 | Allowed (1.1%) *Cg_exo* chi angles: 359.2,15.4,336.5 | 0.03Å | - | - | - |
| A 259 | | PHE | 25.7 | - | Favored (15.06%) General / -94.1,159.7 | Favored (23.2%) *m-10* chi angles: 301.2,321 | 0.04Å | - | - | - |
| A 260 | | SER | 25.64 | - | Favored (31.13%) General / -89.7,120.6 | Favored (6.6%) *t* chi angles: 163.6 | 0.02Å | - | - | - |
| **#** | **Alt** | **Res** | **High B** | **Clash > 0.4Å** | **Ramachandran** | **Rotamer** | **Cβ deviation** | **Bond lengths** | **Bond angles** | **Cis Peptides** |
| | | | Avg: 34.13 | Clashscore: 2.93 | Outliers: 0 of 340 | Poor rotamers: 2 of 294 | Outliers: 0 of 323 | Outliers: 0 of 348 | Outliers: 1 of 348 | Non-Trans: 1 of 345 |
| A 261 | | ARG | 30 | - | Favored (34.46%) General / -140.9,140.6 | Favored (35.3%) *ttt-90* chi angles: 202.4,185.9,184.5,280.8 | 0.02Å | - | - | - |
| A 262 | | ASP | 27.58 | - | Favored (49.43%) General / -114.1,139.3 | Favored (27.4%) *t70* chi angles: 179,78.5 | 0.02Å | - | - | - |
| A 263 | | ASP | 33.48 | - | Favored (77.67%) General / -63.7,-34.4 | Favored (85.3%) *m-30* chi angles: 283.5,345 | 0.02Å | - | - | - |
| A 264 | | ARG | 30 | - | Favored (22.39%) General / -81.4,-41.1 | Favored (4.2%) *ttp80* chi angles: 190.5,173.2,102.4,70.5 | 0.04Å | - | - | - |
| A 265 | | LEU | 23.83 | - | Favored (21.51%) General / -101.9,-8.5 | Favored (78.7%) *mt* chi angles: 300.1,180.5 | 0.04Å | - | - | - |

| | | | | | | | | | | |
|---|---|---|---|---|---|---|---|---|---|---|
| A 266 | GLY | 21.77 | - | Favored (39.17%) Glycine / 73.6,-147.7 | - | - | - | - | - |
| A 267 | PHE | 23.94 | - | Favored (28.66%) General / -66.9,126.3 | Favored (51.2%) *m-80* chi angles: 287.1,292.4 | 0.03Å | - | - | - |
| A 268 | LEU | 24.63 | - | Favored (33.67%) General / -88.2,134.9 | Favored (70.1%) *mt* chi angles: 300.2,182.8 | 0.04Å | - | - | - |
| A 269 | THR | 23.15 | - | Favored (40.5%) General / -133.9,159.5 | Favored (17.5%) *p* chi angles: 49.3 | 0.04Å | - | - | - |
| A 270 | PHE | 22.61 | 0.41Å HD2 with A 225 GLU HG3 | Favored (96.39%) General / -64.5,-41.4 | Favored (25%) *t80* chi angles: 163.9,67.4 | 0.12Å | - | - | - |
| A 271 | CYS | 25.73 | 0.63Å SG with B 1 ARG NE | Favored (20.54%) Pre-Pro / -99.3,136.2 | Favored (46.4%) *t* chi angles: 185.1 | 0.06Å | - | - | - |
| A 272 | PRO | 22.46 | - | Favored (44.35%) Trans-Pro / -62.9,-15.0 | Favored (10%) *Cg_endo* chi angles: 15.2,335.6,23.1 | 0.02Å | - | - | - |
| A 273 | THR | 23.59 | - | Favored (61.79%) General / -72.3,-18.5 | Favored (57.9%) *p* chi angles: 64.5 | 0.05Å | - | - | - |
| A 274 | ASN | 24.83 | - | Favored (19.43%) General / -103.0,19.5 | Favored (35.3%) *m110* chi angles: 294.9,127.9 | 0.03Å | - | - | - |
| A 275 | LEU | 23.62 | - | Favored (3.31%) General / -76.8,-175.1 | Favored (65.3%) *mt* chi angles: 287.1,173.1 | 0.06Å | - | - | - |
| A 276 | GLY | 22.37 | - | Favored (3.52%) Glycine / 71.4,-111.1 | - | - | - | - | - |
| A 277 | THR | 22.25 | - | Favored (60.75%) General / -74.5,-27.8 | Favored (36%) *p* chi angles: 68.5 | 0.05Å | - | - | - |
| A 278 | THR | 23.74 | - | Allowed (1.5%) General / 65.5,0.8 | Favored (2.8%) *p* chi angles: 80.7 | 0.07Å | - | - | - |
| A 279 | ILE | 24.06 | - | Favored (33.35%) Ile or Val / -76.8,132.2 | Allowed (1.4%) *mm* chi angles: 295.8,272.2 | 0.04Å | - | - | - |

| # | Alt | Res | High B | Clash > 0.4Å | Ramachandran | Rotamer | Cβ deviation | Bond lengths | Bond angles | Cis Peptides |
|---|---|---|---|---|---|---|---|---|---|---|
| A 280 | | ARG | 30 | 0.41Å NH2 with A 324 ASP OD2 | Favored (5.93%) General / -128.7,101.0 | Favored (46.1%) *ttt-90* chi angles: 177.6,185.8,194.8,284.3 | 0.03Å | - | - | - |
| **#** | **Alt** | **Res** | **High B** | **Clash > 0.4Å** | **Ramachandran** | **Rotamer** | **Cβ deviation** | **Bond lengths** | **Bond angles** | **Cis Peptides** |
| | | | Avg: 34.13 | Clashscore: 2.93 | Outliers: 0 of 340 | Poor rotamers: 2 of 294 | Outliers: 0 of 323 | Outliers: 0 of 348 | Outliers: 1 of 348 | Non-Trans: 1 of 345 |
| A 281 | | ALA | 22.44 | - | Favored (48.35%) General / -101.6,127.5 | - | 0.03Å | - | - | - |
| A 282 | | SER | 24.79 | - | Favored (43.03%) General / -149.4,160.9 | Favored (75.5%) *p* chi angles: 71.2 | 0.03Å | - | - | - |
| A 283 | | VAL | 27.26 | - | Favored (28.51%) Ile or Val / -124.1,147.4 | Favored (17.4%) *m* chi angles: 293.2 | 0.01Å | - | - | - |
| A 284 | | HIS | 30 | - | Favored (30.32%) General / -92.2,117.9 | Favored (54.5%) *m170* chi angles: 297,171.3 | 0.06Å | - | - | - |
| A 285 | | ILE | 29.68 | - | Favored (25.52%) Ile or Val / -143.2,142.6 | Favored (6.2%) *tt* chi angles: 190.9,179.2 | 0.04Å | - | - | - |
| A 286 | | ALA | 31.69 | - | Favored (49.97%) General / -111.1,123.3 | - | 0.02Å | - | - | - |
| A 287 | | LEU | 35.22 | - | Favored (66.14%) Pre-Pro / -124.9,76.9 | Favored (55.8%) *mt* chi angles: 302.9,171.9 | 0.05Å | - | - | - |
| A 288 | | PRO | 37.7 | - | Favored (26.3%) Trans-Pro / -56.0,-47.1 | Favored (27.3%) *Cg_exo* chi angles: 340.4,30.5,331.4 | 0.01Å | - | - | - |
| A 289 | | LYS | 41.58 | - | Favored (28.64%) General / -63.9,-54.8 | Favored (2.2%) *mttm* chi angles: 300.6,212.4,215.9,317.3 | 0.04Å | - | - | - |
| A 290 | | LEU | 41.93 | - | Favored (69.97%) General / -63.4,-28.4 | Favored (11.4%) *mt* chi angles: 302.9,194.1 | 0.03Å | - | - | - |
| A 291 | | ALA | 45.19 | 0.42Å C with A 293 ASP H | Favored (58.14%) General / -84.9,-9.4 | - | 0.01Å | - | - | - |
| A 292 | | LYS | 47.44 | - | Allowed (0.11%) | Allowed (0.9%) *tttp* | 0.02Å | - | - | - |

| | | | | | | | | | | |
|---|---|---|---|---|---|---|---|---|---|---|
| | | | | | General / -73.3,18.5 | chi angles: 214.1,201.2,151.2,50.1 | | | | |
| A 293 | | ASP | 51.65 | 0.42Å H with A 291 ALA C | Allowed (0.27%) General / -161.3,65.0 | Favored (42.4%) *t0* chi angles: 190.9,359.6 | 0.03Å | - | - | - |
| A 294 | | LYS | 50.31 | - | Favored (29.09%) General / -52.8,-29.7 | Allowed (0.7%) *tptt* chi angles: 165.5,105.1,140.9,152.5 | 0.04Å | - | - | - |
| A 295 | | LYS | 73.29 | - | Favored (76.94%) General / -66.0,-33.9 | Allowed (0.3%) *mptt* chi angles: 258.9,31.5,186.1,145.3 | 0.03Å | - | - | - |
| A 296 | | GLN | 52.52 | - | Favored (27.39%) General / -83.1,-31.3 | Favored (23.4%) *tp40* chi angles: 206.7,63.3,42.4 | 0.02Å | - | - | - |
| A 297 | | LEU | 45.05 | - | Favored (74.92%) General / -59.2,-50.2 | Favored (17.8%) *tp* chi angles: 172.4,72.2 | 0.01Å | - | - | - |
| A 298 | | GLU | 44.64 | - | Favored (85.02%) General / -61.8,-37.6 | OUTLIER (0.3%) chi angles: 277.4,249.3,147.1 | 0.01Å | - | - | - |
| A 299 | | ALA | 45.13 | 0.46Å O with A 303 LYS HG3 | Favored (95.23%) General / -63.4,-39.7 | - | 0.02Å | - | - | - |
| A 300 | | ILE | 44.14 | - | Favored (32.81%) Ile or Val / -67.4,-53.4 | Favored (49.4%) *mt* chi angles: 290.9,158.2 | 0.04Å | - | - | - |
| **#** | **Alt** | **Res** | **High B** | **Clash > 0.4Å** | **Ramachandran** | **Rotamer** | **Cβ deviation** | **Bond lengths** | **Bond angles** | **Cis Peptides** |
| | | | Avg: 34.13 | Clashscore: 2.93 | Outliers: 0 of 340 | Poor rotamers: 2 of 294 | Outliers: 0 of 323 | Outliers: 0 of 348 | Outliers: 1 of 348 | Non-Trans: 1 of 345 |
| A 301 | | ALA | 37.48 | - | Favored (56.55%) General / -50.5,-46.0 | - | 0.04Å | - | - | - |
| A 302 | | ALA | 36.31 | - | Favored (64.15%) General / -56.7,-31.2 | - | 0.03Å | - | - | - |
| A 303 | | LYS | 47.65 | 0.46Å HG3 with A 299 ALA O | Favored (65.59%) General / -72.4,-31.6 | Favored (3.9%) *mttm* chi angles: 316.4,149.9,186.9,281.8 | 0.03Å | - | - | - |
| A 304 | | PHE | 33.45 | - | Favored (16.21%) General / -90.6,13.4 | Favored (62.9%) *m-80* chi angles: 298.9,294.8 | 0.03Å | - | - | - |

| | | | | | | | | | |
|---|---|---|---|---|---|---|---|---|---|
| A 305 | ASN | 29.19 | - | Favored (5.85%) General / 65.5,37.8 | Favored (77.8%) *m-40* chi angles: 282.1,335.4 | 0.05Å | - | - | - |
| A 306 | LEU | 29.42 | - | Favored (25.98%) General / -114.0,155.4 | Favored (30.7%) *mt* chi angles: 298.9,188 | 0.01Å | - | - | - |
| A 307 | GLN | 35.8 | - | Favored (53.67%) General / -122.7,131.2 | OUTLIER (0.2%) chi angles: 98.8,153.3,63.3 | 0.04Å | - | OUTLIER(S) worst is CA-CB-CG: 4.1 σ | - |
| A 308 | VAL | 35.01 | - | Favored (32.51%) Ile or Val / -99.6,136.0 | Favored (14.5%) *t* chi angles: 188.3 | 0.02Å | - | - | - |
| A 309 | ALA | 34.51 | - | Favored (3.17%) General / -143.7,-169.8 | - | 0.03Å | - | - | - |
| A 310 | GLY | 42.64 | 0.73Å O with S 334 HOH O | - | - | - | - | - | - |
| A 321 | GLY | 35.67 | - | - | - | - | - | - | - |
| A 322 | VAL | 34.53 | - | Favored (35.82%) Ile or Val / -86.8,120.2 | Favored (63.2%) *t* chi angles: 171.2 | 0.03Å | - | - | - |
| A 323 | TYR | 41.45 | - | Favored (17.9%) General / -107.8,157.7 | Favored (68.1%) *m-80* chi angles: 286.8,278 | 0.02Å | - | - | - |
| A 324 | ASP | 30.02 | 0.41Å OD2 with A 280 ARG NH2 | Favored (25.13%) General / -98.4,113.2 | Favored (21.6%) *t70* chi angles: 189,82.7 | 0.06Å | - | - | - |
| A 325 | ILE | 27.22 | - | Favored (68.18%) Ile or Val / -111.0,127.3 | Favored (6.6%) *mt* chi angles: 306.5,190.6 | 0.04Å | - | - | - |
| A 326 | SER | 27.34 | - | Favored (38.86%) General / -139.4,160.9 | Favored (72.4%) *p* chi angles: 59.3 | 0.04Å | - | - | - |
| A 327 | ASN | 23.44 | - | Favored (35.26%) General / -65.7,128.3 | Favored (10.2%) *p0* chi angles: 64.6,287.6 | 0.01Å | - | - | - |
| A 328 | LYS | 33.24 | - | Favored (44.58%) General / -78.8,-28.1 | Allowed (0.8%) *tmtt* chi angles: 222.8,285.7,169.5,187 | 0.01Å | - | - | - |
| A 329 | ARG | 30 | - | Favored (33.65%) General / | Favored (74.9%) *mtp180* chi angles: | 0.03Å | - | - | - |

| # | Alt | Res | High B | Clash > 0.4Å | Ramachandran | Rotamer | Cβ deviation | Bond lengths | Bond angles | Cis Peptides |
|---|---|---|---|---|---|---|---|---|---|---|
| | | | | | -87.1,133.2 | 300.3,173.4,65.4,201.8 | | | | |
| A 330 | | ARG | 30 | - | Favored (3.94%) General / -116.7,-36.4 | Favored (44.1%) *mmt-90* chi angles: 307.9,273.6,188.4,271.1 | 0.07Å | - | - | - |
| **#** | **Alt** | **Res** | **High B** | **Clash > 0.4Å** | **Ramachandran** | **Rotamer** | **Cβ deviation** | **Bond lengths** | **Bond angles** | **Cis Peptides** |
| | | | Avg: 34.13 | Clashscore: 2.93 | Outliers: 0 of 340 | Poor rotamers: 2 of 294 | Outliers: 0 of 323 | Outliers: 0 of 348 | Outliers: 1 of 348 | Non-Trans: 1 of 345 |
| A 331 | | MET | 24.79 | - | Favored (33.05%) General / -113.3,150.1 | Favored (81%) *mtp* chi angles: 300.6,184.6,76.1 | 0.03Å | - | - | - |
| A 332 | | GLY | 24.01 | - | Favored (73.68%) Glycine / 90.8,1.2 | - | - | - | - | - |
| A 333 | | LEU | 23.79 | - | Favored (46.11%) General / -140.7,156.8 | Favored (2.1%) *pt* chi angles: 76.1,175.1 | 0.01Å | - | - | - |
| A 334 | | THR | 22.95 | - | Favored (17.39%) General / -81.5,169.3 | Favored (62.5%) *p* chi angles: 63.6 | 0.06Å | - | - | - |
| A 335 | | GLU | 23.85 | - | Favored (86.47%) General / -58.5,-46.5 | Favored (59.9%) *mp0* chi angles: 290.6,79.7,358.4 | 0.01Å | - | - | - |
| A 336 | | TYR | 27.1 | - | Favored (14.84%) General / -51.6,-56.2 | Favored (86.9%) *t80* chi angles: 180.4,82 | 0.02Å | - | - | - |
| A 337 | | GLN | 27.66 | - | Favored (75.11%) General / -58.5,-37.2 | Favored (85%) *mt0* chi angles: 286.8,165.7,334.7 | 0.04Å | - | - | - |
| A 338 | | ALA | 22.17 | - | Favored (71.1%) General / -64.2,-49.7 | - | 0.03Å | - | - | - |
| A 339 | | VAL | 23.16 | - | Favored (91.11%) Ile or Val / -63.2,-41.0 | Favored (70%) *t* chi angles: 172.1 | 0.05Å | - | - | - |
| A 340 | | LYS | 32.17 | - | Favored (70.52%) General / -65.7,-29.9 | Favored (12.3%) *tptp* chi angles: 175.2,65.7,152.8,46.5 | 0.03Å | - | - | - |
| A 341 | | GLU | 30.3 | - | Favored (70.69%) General / -69.1,-45.3 | Allowed (0.7%) *tt0* chi angles: 230.9,164.9,60.7 | 0.04Å | - | - | - |

| | | | | | | | | | | |
|---|---|---|---|---|---|---|---|---|---|---|
| A 342 | | MET | 24.33 | - | Favored (73.11%) General / -56.4,-49.9 | Favored (20.2%) *tmm* chi angles: 187.9,271.8,282.8 | 0.03Å | - | - | - |
| A 343 | | GLN | 28.93 | - | Favored (74.29%) General / -57.8,-50.1 | Favored (9.9%) *tt0* chi angles: 168.1,195.5,283.8 | 0.04Å | - | - | - |
| A 344 | | ASP | 27.75 | - | Favored (87.65%) General / -66.9,-39.7 | Favored (87.3%) *m-30* chi angles: 291.7,350.6 | 0.04Å | - | - | - |
| A 345 | | GLY | 25.78 | - | Favored (97.97%) Glycine / -62.7,-44.1 | - | - | - | - | - |
| A 346 | | ILE | 24.96 | - | Favored (86.6%) Ile or Val / -67.8,-43.2 | Favored (24.9%) *mm* chi angles: 295.1,305.4 | 0.07Å | - | - | - |
| A 347 | | LEU | 28.61 | - | Favored (70.2%) General / -54.2,-42.9 | Favored (73.5%) *mt* chi angles: 287.4,170.4 | 0.02Å | - | - | - |
| A 348 | | GLU | 30.82 | - | Favored (75.99%) General / -69.2,-42.4 | Favored (79.6%) *mm-30* chi angles: 291.1,296.8,129.1 | 0.02Å | - | - | - |
| A 349 | | MET | 32.34 | - | Favored (77.49%) General / -63.9,-34.2 | Favored (73.7%) *mtp* chi angles: 299.1,186.2,66.1 | 0.04Å | - | - | - |
| A 350 | | ILE | 26.48 | - | Favored (81.85%) Ile or Val / -68.9,-41.0 | Favored (29%) *mt* chi angles: 283.4,169.3 | 0.06Å | - | - | - |
| **#** | **Alt** | **Res** | **High B** | **Clash > 0.4Å** | **Ramachandran** | **Rotamer** | **Cβ deviation** | **Bond lengths** | **Bond angles** | **Cis Peptides** |
| | | | Avg: 34.13 | Clashscore: 2.93 | Outliers: 0 of 340 | Poor rotamers: 2 of 294 | Outliers: 0 of 323 | Outliers: 0 of 348 | Outliers: 1 of 348 | Non-Trans: 1 of 345 |
| A 351 | | LYS | 27.76 | - | Favored (73.53%) General / -54.8,-44.1 | Favored (86.5%) *mttt* chi angles: 288.2,183.3,187.6,171.9 | 0.03Å | - | - | - |
| A 352 | | MET | 34.59 | - | Favored (84.92%) General / -65.4,-45.2 | Favored (85%) *mtp* chi angles: 295.9,176.1,62.3 | 0.02Å | - | - | - |
| A 353 | | GLU | 38.05 | - | Favored (98.67%) General / -62.4,-41.4 | Favored (43.3%) *tp30* chi angles: 177.1,74.3,203.4 | 0.04Å | - | - | - |

| | | | | | | | | | | |
|---|---|---|---|---|---|---|---|---|---|---|
| A 354 | | GLU | 34.72 | - | Favored (63.27%) General / -70.1,-24.9 | Allowed (0.8%) *tp30* chi angles: 203,88.6,94.7 | 0.02Å | - | - | - |
| A 355 | | ALA | 36.54 | - | Favored (27.33%) General / -95.9,-11.0 | - | 0.01Å | - | - | - |
| A 356 | | ALA | 39.12 | - | Favored (89.43%) Pre-Pro / -73.1,156.4 | - | 0.01Å | - | - | - |
| A 357 | | PRO | 42.77 | - | - | Favored (40.7%) *Cg_endo* chi angles: 23.5,327,28.5 | 0.02Å | - | - | - |
| B 1 | | ARG | 29.32 | 0.63Å NE with A 271 CYS SG | - | Favored (2.5%) *ptp-110* chi angles: 87.2,189.6,51.1,230.9 | 0.05Å | - | - | - |
| D 3 | | NA | 38.65 | - | - | - | - | - | - | - |
| D 5 | | NA | 48.85 | - | - | - | - | - | - | - |
| S 1 | | HOH | 23.41 | - | - | - | - | - | - | - |
| S 2 | | HOH | 23.54 | - | - | - | - | - | - | - |
| S 3 | | HOH | 27.26 | - | - | - | - | - | - | - |
| S 4 | | HOH | 26.02 | - | - | - | - | - | - | - |
| S 5 | | HOH | 25.17 | - | - | - | - | - | - | - |
| S 6 | | HOH | 23.45 | - | - | - | - | - | - | - |
| S 7 | | HOH | 19.68 | - | - | - | - | - | - | - |
| S 8 | | HOH | 22.08 | - | - | - | - | - | - | - |
| S 9 | | HOH | 25.07 | - | - | - | - | - | - | - |
| S 10 | | HOH | 20.82 | - | - | - | - | - | - | - |
| **#** | **Alt** | **Res** | **High B** | **Clash > 0.4Å** | **Ramachandran** | **Rotamer** | **Cβ deviation** | **Bond lengths** | **Bond angles** | **Cis Peptides** |
| | | | Avg: 34.13 | Clashscore: 2.93 | Outliers: 0 of 340 | Poor rotamers: 2 of 294 | Outliers: 0 of 323 | Outliers: 0 of 348 | Outliers: 1 of 348 | Non-Trans: 1 of 345 |
| S 11 | | HOH | 22.54 | - | - | - | - | - | - | - |
| S 12 | | HOH | 30.7 | - | - | - | - | - | - | - |
| S 13 | | HOH | 27.36 | - | - | - | - | - | - | - |
| S 14 | | HOH | 23.39 | - | - | - | - | - | - | - |
| S 15 | | HOH | 27.81 | - | - | - | - | - | - | - |

| | | | | | | | | | | |
|---|---|---|---|---|---|---|---|---|---|---|
| S 16 | | HOH | 30.4 | - | - | - | - | - | - | - |
| S 17 | | HOH | 28.57 | - | - | - | - | - | - | - |
| S 18 | | HOH | 25.55 | - | - | - | - | - | - | - |
| S 19 | | HOH | 25.28 | - | - | - | - | - | - | - |
| S 20 | | HOH | 28.12 | - | - | - | - | - | - | - |
| S 21 | | HOH | 23.54 | - | - | - | - | - | - | - |
| S 22 | | HOH | 25.01 | - | - | - | - | - | - | - |
| S 23 | | HOH | 30.2 | - | - | - | - | - | - | - |
| S 24 | | HOH | 33.57 | - | - | - | - | - | - | - |
| S 25 | | HOH | 28.38 | - | - | - | - | - | - | - |
| S 26 | | HOH | 26.31 | - | - | - | - | - | - | - |
| S 27 | | HOH | 21.41 | - | - | - | - | - | - | - |
| S 28 | | HOH | 25.99 | - | - | - | - | - | - | - |
| S 29 | | HOH | 30.37 | - | - | - | - | - | - | - |
| S 30 | | HOH | 31.76 | - | - | - | - | - | - | - |
| **#** | **Alt** | **Res** | **High B** | **Clash > 0.4Å** | **Ramachandran** | **Rotamer** | **Cβ deviation** | **Bond lengths** | **Bond angles** | **Cis Peptides** |
| | | | Avg: 34.13 | Clashscore: 2.93 | Outliers: 0 of 340 | Poor rotamers: 2 of 294 | Outliers: 0 of 323 | Outliers: 0 of 348 | Outliers: 1 of 348 | Non-Trans: 1 of 345 |
| S 31 | | HOH | 26.43 | - | - | - | - | - | - | - |
| S 32 | | HOH | 29.23 | - | - | - | - | - | - | - |
| S 33 | | HOH | 25.06 | - | - | - | - | - | - | - |
| S 34 | | HOH | 24.84 | - | - | - | - | - | - | - |
| S 35 | | HOH | 17.91 | - | - | - | - | - | - | - |
| S 36 | | HOH | 30.8 | - | - | - | - | - | - | - |

| | | | | | | | | | | |
|---|---|---|---|---|---|---|---|---|---|---|
| S 37 | | HOH | 27.29 | - | - | - | - | - | - | - |
| S 38 | | HOH | 31.24 | - | - | - | - | - | - | - |
| S 39 | | HOH | 32.03 | - | - | - | - | - | - | - |
| S 40 | | HOH | 30.51 | - | - | - | - | - | - | - |
| S 41 | | HOH | 29.88 | - | - | - | - | - | - | - |
| S 42 | | HOH | 28.83 | - | - | - | - | - | - | - |
| S 43 | | HOH | 24.48 | - | - | - | - | - | - | - |
| S 44 | | HOH | 25.96 | - | - | - | - | - | - | - |
| S 45 | | HOH | 25.14 | - | - | - | - | - | - | - |
| S 46 | | HOH | 26.79 | - | - | - | - | - | - | - |
| S 47 | | HOH | 29.46 | - | - | - | - | - | - | - |
| S 48 | | HOH | 27.52 | - | - | - | - | - | - | - |
| S 49 | | HOH | 24.04 | - | - | - | - | - | - | - |
| S 50 | | HOH | 23.26 | - | - | - | - | - | - | - |
| # | Alt | Res | High B | Clash > 0.4Å | Ramachandran | Rotamer | Cβ deviation | Bond lengths | Bond angles | Cis Peptides |
| | | | Avg: 34.13 | Clashscore: 2.93 | Outliers: 0 of 340 | Poor rotamers: 2 of 294 | Outliers: 0 of 323 | Outliers: 0 of 348 | Outliers: 1 of 348 | Non-Trans: 1 of 345 |
| S 51 | | HOH | 28.19 | - | - | - | - | - | - | - |
| S 52 | | HOH | 27 | - | - | - | - | - | - | - |
| S 53 | | HOH | 27.17 | - | - | - | - | - | - | - |
| S 54 | | HOH | 28.85 | - | - | - | - | - | - | - |
| S 55 | | HOH | 36.58 | - | - | - | - | - | - | - |
| S 56 | | HOH | 27.48 | - | - | - | - | - | - | - |
| S 57 | | HOH | 23.42 | - | - | - | - | - | - | - |

| # | Alt | Res | High B | Clash > 0.4Å | Ramachandran | Rotamer | Cβ deviation | Bond lengths | Bond angles | Cis Peptides |
|---|---|---|---|---|---|---|---|---|---|---|
| S 58 | | HOH | 26 | - | - | - | - | - | - | - |
| S 59 | | HOH | 32.04 | - | - | - | - | - | - | - |
| S 60 | | HOH | 21.63 | - | - | - | - | - | - | - |
| S 61 | | HOH | 25.84 | - | - | - | - | - | - | - |
| S 62 | | HOH | 29.82 | - | - | - | - | - | - | - |
| S 63 | | HOH | 32.54 | - | - | - | - | - | - | - |
| S 64 | | HOH | 25.69 | - | - | - | - | - | - | - |
| S 65 | | HOH | 26.91 | - | - | - | - | - | - | - |
| S 66 | | HOH | 29.26 | - | - | - | - | - | - | - |
| S 67 | | HOH | 27.33 | - | - | - | - | - | - | - |
| S 68 | | HOH | 33.39 | - | - | - | - | - | - | - |
| S 69 | | HOH | 27.24 | - | - | - | - | - | - | - |
| S 70 | | HOH | 33.31 | - | - | - | - | - | - | - |
| **#** | **Alt** | **Res** | **High B** | **Clash > 0.4Å** | **Ramachandran** | **Rotamer** | **Cβ deviation** | **Bond lengths** | **Bond angles** | **Cis Peptides** |
| | | | Avg: 34.13 | Clashscore: 2.93 | Outliers: 0 of 340 | Poor rotamers: 2 of 294 | Outliers: 0 of 323 | Outliers: 0 of 348 | Outliers: 1 of 348 | Non-Trans: 1 of 345 |
| S 71 | | HOH | 28.41 | - | - | - | - | - | - | - |
| S 72 | | HOH | 28.55 | - | - | - | - | - | - | - |
| S 73 | | HOH | 34.31 | - | - | - | - | - | - | - |
| S 74 | | HOH | 27.01 | - | - | - | - | - | - | - |
| S 75 | | HOH | 29.95 | - | - | - | - | - | - | - |
| S 76 | | HOH | 37.28 | - | - | - | - | - | - | - |
| S 77 | | HOH | 30.25 | 0.64Å O with A 147 GLU OE1 | - | - | - | - | - | - |
| S 78 | | HOH | 29.02 | - | - | - | - | - | - | - |

| | | | | | | | | | | |
|---|---|---|---|---|---|---|---|---|---|---|
| S 79 | | HOH | 32.72 | - | - | - | - | - | - | - |
| S 80 | | HOH | 29.66 | - | - | - | - | - | - | - |
| S 81 | | HOH | 31.1 | - | - | - | - | - | - | - |
| S 82 | | HOH | 32.14 | - | - | - | - | - | - | - |
| S 83 | | HOH | 32.03 | - | - | - | - | - | - | - |
| S 84 | | HOH | 27.41 | - | - | - | - | - | - | - |
| S 85 | | HOH | 27.77 | - | - | - | - | - | - | - |
| S 86 | | HOH | 23.9 | - | - | - | - | - | - | - |
| S 87 | | HOH | 30.08 | - | - | - | - | - | - | - |
| S 88 | | HOH | 30.39 | - | - | - | - | - | - | - |
| S 89 | | HOH | 32.92 | - | - | - | - | - | - | - |
| S 90 | | HOH | 30.04 | - | - | - | - | - | - | - |
| **#** | **Alt** | **Res** | **High B** | **Clash > 0.4Å** | **Ramachandran** | **Rotamer** | **Cβ deviation** | **Bond lengths** | **Bond angles** | **Cis Peptides** |
| | | | Avg: 34.13 | Clashscore: 2.93 | Outliers: 0 of 340 | Poor rotamers: 2 of 294 | Outliers: 0 of 323 | Outliers: 0 of 348 | Outliers: 1 of 348 | Non-Trans: 1 of 345 |
| S 91 | | HOH | 32.99 | - | - | - | - | - | - | - |
| S 92 | | HOH | 35.07 | - | - | - | - | - | - | - |
| S 93 | | HOH | 33.02 | - | - | - | - | - | - | - |
| S 94 | | HOH | 24.03 | - | - | - | - | - | - | - |
| S 95 | | HOH | 29.91 | - | - | - | - | - | - | - |
| S 96 | | HOH | 26.01 | - | - | - | - | - | - | - |
| S 97 | | HOH | 36.55 | - | - | - | - | - | - | - |
| S 98 | | HOH | 30.99 | - | - | - | - | - | - | - |
| S 99 | | HOH | 28.07 | - | - | - | - | - | - | - |

| # | Alt | Res | High B | Clash > 0.4Å | Ramachandran | Rotamer | Cβ deviation | Bond lengths | Bond angles | Cis Peptides |
|---|---|---|---|---|---|---|---|---|---|---|
| S 100 | | HOH | 26.96 | - | - | - | - | - | - | - |
| S 101 | | HOH | 33.91 | - | - | - | - | - | - | - |
| S 102 | | HOH | 35.07 | - | - | - | - | - | - | - |
| S 103 | | HOH | 36.17 | - | - | - | - | - | - | - |
| S 104 | | HOH | 29.11 | - | - | - | - | - | - | - |
| S 105 | | HOH | 32.7 | - | - | - | - | - | - | - |
| S 106 | | HOH | 22.9 | - | - | - | - | - | - | - |
| S 107 | | HOH | 31.01 | - | - | - | - | - | - | - |
| S 108 | | HOH | 38.95 | - | - | - | - | - | - | - |
| S 109 | | HOH | 38.32 | - | - | - | - | - | - | - |
| S 110 | | HOH | 38.34 | - | - | - | - | - | - | - |
| **#** | **Alt** | **Res** | **High B** | **Clash > 0.4Å** | **Ramachandran** | **Rotamer** | **Cβ deviation** | **Bond lengths** | **Bond angles** | **Cis Peptides** |
| | | | Avg: 34.13 | Clashscore: 2.93 | Outliers: 0 of 340 | Poor rotamers: 2 of 294 | Outliers: 0 of 323 | Outliers: 0 of 348 | Outliers: 1 of 348 | Non-Trans: 1 of 345 |
| S 111 | | HOH | 31.17 | - | - | - | - | - | - | - |
| S 112 | | HOH | 24.21 | - | - | - | - | - | - | - |
| S 113 | | HOH | 35.38 | - | - | - | - | - | - | - |
| S 114 | | HOH | 31.08 | - | - | - | - | - | - | - |
| S 115 | | HOH | 27.97 | - | - | - | - | - | - | - |
| S 116 | | HOH | 28.31 | - | - | - | - | - | - | - |
| S 117 | | HOH | 25.92 | - | - | - | - | - | - | - |
| S 118 | | HOH | 35.67 | - | - | - | - | - | - | - |
| S 119 | | HOH | 27.95 | - | - | - | - | - | - | - |
| S 120 | | HOH | 38.91 | - | - | - | - | - | - | - |

| # | Alt | Res | High B | Clash > 0.4Å | Ramachandran | Rotamer | Cβ deviation | Bond lengths | Bond angles | Cis Peptides |
|---|---|---|---|---|---|---|---|---|---|---|
| S 121 | | HOH | 37.34 | - | - | - | - | - | - | - |
| S 122 | | HOH | 32.65 | - | - | - | - | - | - | - |
| S 123 | | HOH | 36.27 | - | - | - | - | - | - | - |
| S 124 | | HOH | 47.54 | - | - | - | - | - | - | - |
| S 125 | | HOH | 35.54 | - | - | - | - | - | - | - |
| S 126 | | HOH | 31.37 | - | - | - | - | - | - | - |
| S 127 | | HOH | 32.59 | - | - | - | - | - | - | - |
| S 128 | | HOH | 25.42 | - | - | - | - | - | - | - |
| S 129 | | HOH | 40.11 | - | - | - | - | - | - | - |
| S 130 | | HOH | 27.11 | - | - | - | - | - | - | - |
| **#** | **Alt** | **Res** | **High B** | **Clash > 0.4Å** | **Ramachandran** | **Rotamer** | **Cβ deviation** | **Bond lengths** | **Bond angles** | **Cis Peptides** |
| | | | Avg: 34.13 | Clashscore: 2.93 | Outliers: 0 of 340 | Poor rotamers: 2 of 294 | Outliers: 0 of 323 | Outliers: 0 of 348 | Outliers: 1 of 348 | Non-Trans: 1 of 345 |
| S 131 | | HOH | 33.95 | - | - | - | - | - | - | - |
| S 132 | | HOH | 28.57 | - | - | - | - | - | - | - |
| S 133 | | HOH | 47.03 | - | - | - | - | - | - | - |
| S 134 | | HOH | 31.47 | - | - | - | - | - | - | - |
| S 135 | | HOH | 32.21 | - | - | - | - | - | - | - |
| S 136 | | HOH | 29.37 | - | - | - | - | - | - | - |
| S 137 | | HOH | 31.43 | - | - | - | - | - | - | - |
| S 138 | | HOH | 35.44 | - | - | - | - | - | - | - |
| S 139 | | HOH | 24.94 | - | - | - | - | - | - | - |
| S 140 | | HOH | 34.87 | - | - | - | - | - | - | - |
| S 141 | | HOH | 34.4 | - | - | - | - | - | - | - |

| # | Alt | Res | High B | Clash > 0.4Å | Ramachandran | Rotamer | Cβ deviation | Bond lengths | Bond angles | Cis Peptides |
|---|---|---|---|---|---|---|---|---|---|---|
| S 142 | | HOH | 33.6 | - | - | - | - | - | - | - |
| S 143 | | HOH | 23.72 | - | - | - | - | - | - | - |
| S 144 | | HOH | 39.85 | - | - | - | - | - | - | - |
| S 145 | | HOH | 37.96 | - | - | - | - | - | - | - |
| S 146 | | HOH | 31.37 | - | - | - | - | - | - | - |
| S 147 | | HOH | 37.17 | - | - | - | - | - | - | - |
| S 148 | | HOH | 26.28 | - | - | - | - | - | - | - |
| S 149 | | HOH | 37.92 | - | - | - | - | - | - | - |
| S 150 | | HOH | 33.88 | - | - | - | - | - | - | - |
| # | Alt | Res | High B | Clash > 0.4Å | Ramachandran | Rotamer | Cβ deviation | Bond lengths | Bond angles | Cis Peptides |
| | | | Avg: 34.13 | Clashscore: 2.93 | Outliers: 0 of 340 | Poor rotamers: 2 of 294 | Outliers: 0 of 323 | Outliers: 0 of 348 | Outliers: 1 of 348 | Non-Trans: 1 of 345 |
| S 151 | | HOH | 27.31 | - | - | - | - | - | - | - |
| S 152 | | HOH | 34.95 | - | - | - | - | - | - | - |
| S 153 | | HOH | 48.92 | - | - | - | - | - | - | - |
| S 154 | | HOH | 28.55 | - | - | - | - | - | - | - |
| S 155 | | HOH | 35.85 | - | - | - | - | - | - | - |
| S 156 | | HOH | 37.35 | - | - | - | - | - | - | - |
| S 157 | | HOH | 22.01 | - | - | - | - | - | - | - |
| S 158 | | HOH | 38.28 | - | - | - | - | - | - | - |
| S 159 | | HOH | 29.06 | - | - | - | - | - | - | - |
| S 160 | | HOH | 31.8 | - | - | - | - | - | - | - |
| S 161 | | HOH | 31.74 | - | - | - | - | - | - | - |
| S 162 | | HOH | 34.27 | - | - | - | - | - | - | - |

| # | Alt | Res | High B | Clash > 0.4Å | Ramachandran | Rotamer | Cβ deviation | Bond lengths | Bond angles | Cis Peptides |
|---|---|---|---|---|---|---|---|---|---|---|
| S 163 | | HOH | 36.35 | - | - | - | - | - | - | - |
| S 164 | | HOH | 30.88 | - | - | - | - | - | - | - |
| S 165 | | HOH | 33.09 | - | - | - | - | - | - | - |
| S 166 | | HOH | 30.64 | - | - | - | - | - | - | - |
| S 167 | | HOH | 51.47 | - | - | - | - | - | - | - |
| S 168 | | HOH | 35.98 | - | - | - | - | - | - | - |
| S 169 | | HOH | 39.07 | - | - | - | - | - | - | - |
| S 170 | | HOH | 52.01 | - | - | - | - | - | - | - |
| **#** | **Alt** | **Res** | **High B** | **Clash > 0.4Å** | **Ramachandran** | **Rotamer** | **Cβ deviation** | **Bond lengths** | **Bond angles** | **Cis Peptides** |
| | | | Avg: 34.13 | Clashscore: 2.93 | Outliers: 0 of 340 | Poor rotamers: 2 of 294 | Outliers: 0 of 323 | Outliers: 0 of 348 | Outliers: 1 of 348 | Non-Trans: 1 of 345 |
| S 171 | | HOH | 26.97 | - | - | - | - | - | - | - |
| S 172 | | HOH | 40.49 | - | - | - | - | - | - | - |
| S 173 | | HOH | 38.27 | - | - | - | - | - | - | - |
| S 174 | | HOH | 38.47 | - | - | - | - | - | - | - |
| S 175 | | HOH | 49.04 | - | - | - | - | - | - | - |
| S 176 | | HOH | 45.91 | - | - | - | - | - | - | - |
| S 177 | | HOH | 33.34 | - | - | - | - | - | - | - |
| S 178 | | HOH | 30.88 | - | - | - | - | - | - | - |
| S 179 | | HOH | 33.5 | - | - | - | - | - | - | - |
| S 180 | | HOH | 35.1 | - | - | - | - | - | - | - |
| S 181 | | HOH | 31.59 | - | - | - | - | - | - | - |
| S 182 | | HOH | 43.12 | - | - | - | - | - | - | - |
| S 183 | | HOH | 45.14 | - | - | - | - | - | - | - |

| # | Alt | Res | High B | Clash > 0.4Å | Ramachandran | Rotamer | Cβ deviation | Bond lengths | Bond angles | Cis Peptides |
|---|---|---|---|---|---|---|---|---|---|---|
| S 184 | | HOH | 35.07 | - | - | - | - | - | - | - |
| S 185 | | HOH | 40.72 | - | - | - | - | - | - | - |
| S 186 | | HOH | 41.63 | - | - | - | - | - | - | - |
| S 187 | | HOH | 42.6 | - | - | - | - | - | - | - |
| S 188 | | HOH | 49.56 | - | - | - | - | - | - | - |
| S 189 | | HOH | 46.35 | - | - | - | - | - | - | - |
| S 190 | | HOH | 30.12 | - | - | - | - | - | - | - |
| **#** | **Alt** | **Res** | **High B** | **Clash > 0.4Å** | **Ramachandran** | **Rotamer** | **Cβ deviation** | **Bond lengths** | **Bond angles** | **Cis Peptides** |
| | | | Avg: 34.13 | Clashscore: 2.93 | Outliers: 0 of 340 | Poor rotamers: 2 of 294 | Outliers: 0 of 323 | Outliers: 0 of 348 | Outliers: 1 of 348 | Non-Trans: 1 of 345 |
| S 191 | | HOH | 34.72 | - | - | - | - | - | - | - |
| S 192 | | HOH | 37.77 | - | - | - | - | - | - | - |
| S 193 | | HOH | 41.16 | - | - | - | - | - | - | - |
| S 194 | | HOH | 39.97 | - | - | - | - | - | - | - |
| S 195 | | HOH | 41.94 | - | - | - | - | - | - | - |
| S 196 | | HOH | 39.03 | - | - | - | - | - | - | - |
| S 197 | | HOH | 51.01 | - | - | - | - | - | - | - |
| S 198 | | HOH | 49.96 | - | - | - | - | - | - | - |
| S 199 | | HOH | 33.98 | - | - | - | - | - | - | - |
| S 200 | | HOH | 38.64 | - | - | - | - | - | - | - |
| S 201 | | HOH | 37.79 | - | - | - | - | - | - | - |
| S 202 | | HOH | 51.04 | - | - | - | - | - | - | - |
| S 203 | | HOH | 45.05 | - | - | - | - | - | - | - |
| S 204 | | HOH | 47.8 | - | - | - | - | - | - | - |

| # | Alt | Res | High B | Clash > 0.4Å | Ramachandran | Rotamer | Cβ deviation | Bond lengths | Bond angles | Cis Peptides |
|---|---|---|---|---|---|---|---|---|---|---|
| S 205 | | HOH | 33.44 | - | - | - | - | - | - | - |
| S 206 | | HOH | 33.71 | - | - | - | - | - | - | - |
| S 207 | | HOH | 49.62 | - | - | - | - | - | - | - |
| S 208 | | HOH | 43.7 | - | - | - | - | - | - | - |
| S 209 | | HOH | 43.78 | - | - | - | - | - | - | - |
| S 210 | | HOH | 49.24 | - | - | - | - | - | - | - |
| # | Alt | Res | High B | Clash > 0.4Å | Ramachandran | Rotamer | Cβ deviation | Bond lengths | Bond angles | Cis Peptides |
| | | | Avg: 34.13 | Clashscore: 2.93 | Outliers: 0 of 340 | Poor rotamers: 2 of 294 | Outliers: 0 of 323 | Outliers: 0 of 348 | Outliers: 1 of 348 | Non-Trans: 1 of 345 |
| S 211 | | HOH | 50.3 | - | - | - | - | - | - | - |
| S 212 | | HOH | 44.22 | - | - | - | - | - | - | - |
| S 213 | | HOH | 46.01 | 0.67Å O with A 132 LYS NZ | - | - | - | - | - | - |
| S 214 | | HOH | 37.38 | - | - | - | - | - | - | - |
| S 215 | | HOH | 36.44 | - | - | - | - | - | - | - |
| S 216 | | HOH | 43.41 | - | - | - | - | - | - | - |
| S 217 | | HOH | 39.5 | - | - | - | - | - | - | - |
| S 218 | | HOH | 55.22 | - | - | - | - | - | - | - |
| S 219 | | HOH | 27.22 | - | - | - | - | - | - | - |
| S 220 | | HOH | 30.68 | - | - | - | - | - | - | - |
| S 221 | | HOH | 49.24 | - | - | - | - | - | - | - |
| S 222 | | HOH | 53.87 | - | - | - | - | - | - | - |
| S 223 | | HOH | 43.36 | - | - | - | - | - | - | - |
| S 224 | | HOH | 51.51 | - | - | - | - | - | - | - |
| S 225 | | HOH | 41.35 | - | - | - | - | - | - | - |

| # | Alt | Res | High B | Clash > 0.4Å | Ramachandran | Rotamer | Cβ deviation | Bond lengths | Bond angles | Cis Peptides |
|---|---|---|---|---|---|---|---|---|---|---|
| S 226 | | HOH | 30.88 | - | - | - | - | - | - | - |
| S 227 | | HOH | 45.5 | - | - | - | - | - | - | - |
| S 228 | | HOH | 49.58 | - | - | - | - | - | - | - |
| S 229 | | HOH | 37.84 | - | - | - | - | - | - | - |
| S 230 | | HOH | 37.74 | - | - | - | - | - | - | - |
| **#** | **Alt** | **Res** | **High B** | **Clash > 0.4Å** | **Ramachandran** | **Rotamer** | **Cβ deviation** | **Bond lengths** | **Bond angles** | **Cis Peptides** |
| | | | Avg: 34.13 | Clashscore: 2.93 | Outliers: 0 of 340 | Poor rotamers: 2 of 294 | Outliers: 0 of 323 | Outliers: 0 of 348 | Outliers: 1 of 348 | Non-Trans: 1 of 345 |
| S 231 | | HOH | 46.62 | - | - | - | - | - | - | - |
| S 232 | | HOH | 46.52 | - | - | - | - | - | - | - |
| S 233 | | HOH | 41.89 | - | - | - | - | - | - | - |
| S 234 | | HOH | 45.05 | - | - | - | - | - | - | - |
| S 235 | | HOH | 42.62 | - | - | - | - | - | - | - |
| S 236 | | HOH | 48.18 | - | - | - | - | - | - | - |
| S 237 | | HOH | 38.77 | 0.72Å O with A 162 GLU OE1 | - | - | - | - | - | - |
| S 238 | | HOH | 53.71 | - | - | - | - | - | - | - |
| S 239 | | HOH | 44.46 | - | - | - | - | - | - | - |
| S 240 | | HOH | 48.85 | - | - | - | - | - | - | - |
| S 241 | | HOH | 40.03 | - | - | - | - | - | - | - |
| S 242 | | HOH | 38.87 | - | - | - | - | - | - | - |
| S 243 | | HOH | 40.68 | - | - | - | - | - | - | - |
| S 244 | | HOH | 58.94 | - | - | - | - | - | - | - |
| S 245 | | HOH | 46.87 | - | - | - | - | - | - | - |
| S 246 | | HOH | 49.76 | - | - | - | - | - | - | - |

| # | Alt | Res | High B | Clash > 0.4Å | Ramachandran | Rotamer | Cβ deviation | Bond lengths | Bond angles | Cis Peptides |
|---|---|---|---|---|---|---|---|---|---|---|
| S 247 | | HOH | 48.41 | - | - | - | - | - | - | - |
| S 248 | | HOH | 42.06 | - | - | - | - | - | - | - |
| S 249 | | HOH | 31.2 | - | - | - | - | - | - | - |
| S 250 | | HOH | 39.91 | - | - | - | - | - | - | - |
| # | Alt | Res | High B | Clash > 0.4Å | Ramachandran | Rotamer | Cβ deviation | Bond lengths | Bond angles | Cis Peptides |
| | | | Avg: 34.13 | Clashscore: 2.93 | Outliers: 0 of 340 | Poor rotamers: 2 of 294 | Outliers: 0 of 323 | Outliers: 0 of 348 | Outliers: 1 of 348 | Non-Trans: 1 of 345 |
| S 251 | | HOH | 47.73 | 0.47Å O with A 1 ALA N | - | - | - | - | - | - |
| S 252 | | HOH | 52.67 | - | - | - | - | - | - | - |
| S 253 | | HOH | 43.01 | - | - | - | - | - | - | - |
| S 254 | | HOH | 49.13 | - | - | - | - | - | - | - |
| S 255 | | HOH | 53.62 | - | - | - | - | - | - | - |
| S 256 | | HOH | 33.34 | - | - | - | - | - | - | - |
| S 257 | | HOH | 45.97 | - | - | - | - | - | - | - |
| S 258 | | HOH | 48.12 | - | - | - | - | - | - | - |
| S 259 | | HOH | 41.11 | - | - | - | - | - | - | - |
| S 260 | | HOH | 42.95 | - | - | - | - | - | - | - |
| S 261 | | HOH | 46.83 | - | - | - | - | - | - | - |
| S 262 | | HOH | 32.81 | - | - | - | - | - | - | - |
| S 263 | | HOH | 43.66 | - | - | - | - | - | - | - |
| S 264 | | HOH | 46.28 | - | - | - | - | - | - | - |
| S 265 | | HOH | 24.79 | - | - | - | - | - | - | - |
| S 266 | | HOH | 44.96 | - | - | - | - | - | - | - |
| S 267 | | HOH | 51.72 | - | - | - | - | - | - | - |

| # | Alt | Res | High B | Clash > 0.4Å | Ramachandran | Rotamer | Cβ deviation | Bond lengths | Bond angles | Cis Peptides |
|---|---|---|---|---|---|---|---|---|---|---|
| S 268 | | HOH | 47.41 | - | - | - | - | - | - | - |
| S 269 | | HOH | 44.83 | - | - | - | - | - | - | - |
| S 270 | | HOH | 33.25 | - | - | - | - | - | - | - |
| # | Alt | Res | High B | Clash > 0.4Å | Ramachandran | Rotamer | Cβ deviation | Bond lengths | Bond angles | Cis Peptides |
| | | | Avg: 34.13 | Clashscore: 2.93 | Outliers: 0 of 340 | Poor rotamers: 2 of 294 | Outliers: 0 of 323 | Outliers: 0 of 348 | Outliers: 1 of 348 | Non-Trans: 1 of 345 |
| S 271 | | HOH | 35.34 | - | - | - | - | - | - | - |
| S 272 | | HOH | 52.47 | - | - | - | - | - | - | - |
| S 273 | | HOH | 42.62 | - | - | - | - | - | - | - |
| S 274 | | HOH | 46.72 | - | - | - | - | - | - | - |
| S 275 | | HOH | 45.46 | - | - | - | - | - | - | - |
| S 276 | | HOH | 39.97 | - | - | - | - | - | - | - |
| S 277 | | HOH | 60.45 | - | - | - | - | - | - | - |
| S 278 | | HOH | 54.7 | - | - | - | - | - | - | - |
| S 279 | | HOH | 48.2 | - | - | - | - | - | - | - |
| S 280 | | HOH | 55.15 | - | - | - | - | - | - | - |
| S 281 | | HOH | 49.64 | - | - | - | - | - | - | - |
| S 282 | | HOH | 49.24 | - | - | - | - | - | - | - |
| S 283 | | HOH | 58.09 | - | - | - | - | - | - | - |
| S 284 | | HOH | 50.01 | - | - | - | - | - | - | - |
| S 285 | | HOH | 39.72 | - | - | - | - | - | - | - |
| S 286 | | HOH | 38.76 | - | - | - | - | - | - | - |
| S 287 | | HOH | 35.23 | - | - | - | - | - | - | - |
| S 288 | | HOH | 38.55 | - | - | - | - | - | - | - |

| # | Alt | Res | High B | Clash > 0.4Å | Ramachandran | Rotamer | Cβ deviation | Bond lengths | Bond angles | Cis Peptides |
|---|---|---|---|---|---|---|---|---|---|---|
| S 289 | | HOH | 50 | - | - | - | - | - | - | - |
| S 290 | | HOH | 45.97 | - | - | - | - | - | - | - |
| # | Alt | Res | High B | Clash > 0.4Å | Ramachandran | Rotamer | Cβ deviation | Bond lengths | Bond angles | Cis Peptides |
| | | | Avg: 34.13 | Clashscore: 2.93 | Outliers: 0 of 340 | Poor rotamers: 2 of 294 | Outliers: 0 of 323 | Outliers: 0 of 348 | Outliers: 1 of 348 | Non-Trans: 1 of 345 |
| S 291 | | HOH | 48.69 | - | - | - | - | - | - | - |
| S 292 | | HOH | 52.11 | - | - | - | - | - | - | - |
| S 293 | | HOH | 44.85 | - | - | - | - | - | - | - |
| S 294 | | HOH | 45.8 | - | - | - | - | - | - | - |
| S 295 | | HOH | 41.12 | - | - | - | - | - | - | - |
| S 296 | | HOH | 35.27 | - | - | - | - | - | - | - |
| S 297 | | HOH | 46.71 | - | - | - | - | - | - | - |
| S 298 | | HOH | 36.49 | - | - | - | - | - | - | - |
| S 299 | | HOH | 44.13 | - | - | - | - | - | - | - |
| S 300 | | HOH | 39.32 | - | - | - | - | - | - | - |
| S 301 | | HOH | 57.19 | - | - | - | - | - | - | - |
| S 302 | | HOH | 52.23 | - | - | - | - | - | - | - |
| S 303 | | HOH | 43.31 | - | - | - | - | - | - | - |
| S 304 | | HOH | 54.17 | - | - | - | - | - | - | - |
| S 305 | | HOH | 44.2 | - | - | - | - | - | - | - |
| S 306 | | HOH | 48.59 | - | - | - | - | - | - | - |
| S 307 | | HOH | 53.15 | - | - | - | - | - | - | - |
| S 308 | | HOH | 36.2 | - | - | - | - | - | - | - |
| S 309 | | HOH | 41.84 | - | - | - | - | - | - | - |

| # | Alt | Res | High B | Clash > 0.4Å | Ramachandran | Rotamer | Cβ deviation | Bond lengths | Bond angles | Cis Peptides |
|---|---|---|---|---|---|---|---|---|---|---|
| S 310 | | HOH | 51.07 | - | - | - | - | - | - | - |
| # | Alt | Res | High B | Clash > 0.4Å | Ramachandran | Rotamer | Cβ deviation | Bond lengths | Bond angles | Cis Peptides |
| | | | Avg: 34.13 | Clashscore: 2.93 | Outliers: 0 of 340 | Poor rotamers: 2 of 294 | Outliers: 0 of 323 | Outliers: 0 of 348 | Outliers: 1 of 348 | Non-Trans: 1 of 345 |
| S 311 | | HOH | 54.41 | - | - | - | - | - | - | - |
| S 312 | | HOH | 59.45 | - | - | - | - | - | - | - |
| S 313 | | HOH | 74.24 | - | - | - | - | - | - | - |
| S 314 | | HOH | 54.57 | - | - | - | - | - | - | - |
| S 315 | | HOH | 50.96 | - | - | - | - | - | - | - |
| S 316 | | HOH | 43.59 | - | - | - | - | - | - | - |
| S 317 | | HOH | 47.83 | - | - | - | - | - | - | - |
| S 318 | | HOH | 64.92 | - | - | - | - | - | - | - |
| S 319 | | HOH | 52.51 | - | - | - | - | - | - | - |
| S 320 | | HOH | 33.38 | - | - | - | - | - | - | - |
| S 321 | | HOH | 42.54 | - | - | - | - | - | - | - |
| S 322 | | HOH | 49.21 | - | - | - | - | - | - | - |
| S 324 | | HOH | 53.69 | - | - | - | - | - | - | - |
| S 325 | | HOH | 57.61 | - | - | - | - | - | - | - |
| S 326 | | HOH | 47.38 | - | - | - | - | - | - | - |
| S 327 | | HOH | 61.96 | - | - | - | - | - | - | - |
| S 328 | | HOH | 53.77 | - | - | - | - | - | - | - |
| S 329 | | HOH | 54.33 | - | - | - | - | - | - | - |
| S 330 | | HOH | 58.56 | - | - | - | - | - | - | - |
| S 331 | | HOH | 66.5 | - | - | - | - | - | - | - |

| # | Alt | Res | High B | Clash > 0.4Å | Ramachandran | Rotamer | Cβ deviation | Bond lengths | Bond angles | Cis Peptides |
|---|---|---|---|---|---|---|---|---|---|---|
| | | | Avg: 34.13 | Clashscore: 2.93 | Outliers: 0 of 340 | Poor rotamers: 2 of 294 | Outliers: 0 of 323 | Outliers: 0 of 348 | Outliers: 1 of 348 | Non-Trans: 1 of 345 |
| S 332 | | HOH | 56.58 | - | - | - | - | - | - | - |
| S 333 | | HOH | 58.82 | - | - | - | - | - | - | - |
| S 334 | | HOH | 51.01 | 0.73Å O with A 310 GLY O | - | - | - | - | - | - |
| S 335 | | HOH | 70.34 | - | - | - | - | - | - | - |
| S 336 | | HOH | 54.01 | - | - | - | - | - | - | - |
| S 337 | | HOH | 56.09 | - | - | - | - | - | - | - |
| S 338 | | HOH | 49.21 | - | - | - | - | - | - | - |
| S 339 | | HOH | 61.24 | - | - | - | - | - | - | - |
| S 340 | | HOH | 59.04 | - | - | - | - | - | - | - |
| S 341 | | HOH | 68.85 | - | - | - | - | - | - | - |

About MolProbity | Website for the Richardson Lab | Using ecloud x-H | Internal reference 4.4
