# Supplementary material for: Biochemical and structural characterization of a novel arginine kinase from the spider Polybetes pythagoricus
Source: PeerJ. 2017 Sep 11;5:e3787. doi: 10.7717/peerj.3787 (PMC5598448; doi:10.7717/peerj.3787)
Supplement: Supplemental Information 4 [file peerj-05-3787-s004.pdf]

Mo

Viewing spiderApoAK_UPDATED-5U8EFH-multi.table

When finished, you should close this window . *Hint: Use File | Save As... to save a copy of this page.*

All-Atom Contacts	Clashscore, all atoms:	5		98th percentile* (N=479, 2.180Å ± 0.25Å)
	Clashscore is the number of serious steric overlaps (> 0.4 Å) per 1000 atoms.			
Protein Geometry	Poor rotamers	0	0.00%	Goal: <0.3%
	Favored rotamers	258	89.90%	Goal: >98%
	Ramachandran outliers	1	0.29%	Goal: <0.05%
	Ramachandran favored	331	97.35%	Goal: >98%
	MolProbity score^	1.39		99th percentile* (N=10453, 2.180Å ± 0.25Å)
	Cβ deviations >0.25Å	0	0.00%	Goal: 0
	Bad bonds:	0 / 2767	0.00%	Goal: 0%
	Bad angles:	0 / 3740	0.00%	Goal: <0.1%
Peptide Omegas	Cis Prolines:	1 / 15	6.67%	Expected: ≤1 per chain, or ≤5%

In the two column results, the left column gives the raw count, right column gives the percentage.

* 100th percentile is the best among structures of comparable resolution; 0th percentile is the worst. For clashscore the comparative set of structures was selected in 2004, for MolProbity score in 2006.

^ MolProbity score combines the clashscore, rotamer, and Ramachandran evaluations into a single score, normalized to be on the same scale as X-ray resolution.

#	Alt	Res	High B	Clash > 0.4Å	Ramachandran	Rotamer	Cβ deviation	Bond lengths	Bond angles	Cis Peptides
			Avg: 29.18	Clashscore: 5	Outliers: 1 of 340	Poor rotamers: 0 of 287	Outliers: 0 of 322	Outliers: 0 of 347	Outliers: 0 of 347	Non-Trans: 1 of 345
A 1		ALA	40.25	-	-	-	0.03Å	-	-	-
A 2		VAL	34.77	-	Favored (51.87%) Ile or Val / -98.7,124.4	Favored (47.7%) *t* chi angles: 169.1	0.03Å	-	-	-
A 3		ASP	37.23	-	Favored (36.8%) General / -73.3,157.5	Favored (17.2%) *p0* chi angles: 58.4,36.1	0.06Å	-	-	-
A 4		GLN	33.95	-	Favored (78.15%) General / -61.6,-35.5	Favored (73.7%) *tp40* chi angles: 183,66.5,38.5	0.02Å	-	-	-

A 5	ALA	28.26	-	Favored (81.75%) General / -59.4,-39.0	-	0.02Å	-	-	-
A 6	THR	27.42	0.76Å HG21 with A 49 THR HG21	Favored (76.84%) General / -69.6,-40.2	Favored (33.7%) *m* chi angles: 306.2	0.04Å	-	-	-
A 7	LEU	34.01	-	Favored (90.36%) General / -61.9,-39.0	Favored (95.5%) *mt* chi angles: 294.7,173.5	0.01Å	-	-	-
A 8	ASP	39.26	-	Favored (99.19%) General / -63.2,-42.4	Favored (72.9%) *m-30* chi angles: 295.1,350.2	0.02Å	-	-	-
A 9	LYS	38.44	-	Favored (89.65%) General / -66.0,-38.9	Favored (3.7%) *mttp* chi angles: 298.4,192.8,212.7,90.9	0.01Å	-	-	-
A 10	LEU	22.64	-	Favored (96.09%) General / -63.6,-43.7	Favored (23%) *mt* chi angles: 281.9,161.4	0.03Å	-	-	-
A 11	GLU	30.15	0.85Å OE1 with S 241 HOH O	Favored (97.99%) General / -63.7,-42.3	Favored (44.6%) *mm-30* chi angles: 283.3,286.2,351.4	0.01Å	-	-	-
A 12	ALA	32.11	-	Favored (69.68%) General / -69.6,-31.5	-	0.01Å	-	-	-
A 13	GLY	29.16	-	Favored (87.25%) Glycine / -67.2,-44.1	-	-	-	-	-
A 14	PHE	36.26	-	Favored (75.74%) General / -57.6,-49.6	Favored (83.2%) *t80* chi angles: 177.6,72	0.03Å	-	-	-
A 15	LYS	47.11	-	Favored (91.21%) General / -64.8,-38.5	Allowed (0.5%) *tttm* chi angles: 161.7,197.6,125.7,289.4	0.03Å	-	-	-
A 16	LYS	34.29	-	Favored (83.71%) General / -61.2,-37.6	Favored (41.3%) *tttt* chi angles: 168.3,163,165.2,186.2	0.02Å	-	-	-
A 17	LEU	36.4	-	Favored (86.65%) General / -67.2,-40.6	Favored (51.7%) *mt* chi angles: 284.5,167.8	0.02Å	-	-	-

A 18		GLN	46.73	-	Favored (81.78%) General / -60.6,-48.4	Favored (13.2%) *mm110* chi angles: 308.6,319.1,122.3	0.01Å	-	-	-
A 19		ASP	44.91	-	Favored (64.44%) General / -68.1,-17.6	Favored (49.5%) *m-30* chi angles: 299,349.3	0.02Å	-	-	-
A 20		ALA	39.88	-	Favored (6.18%) General / -81.4,90.2	-	0.02Å	-	-	-
**#**	**Alt**	**Res**	**High B**	**Clash > 0.4Å**	**Ramachandran**	**Rotamer**	**Cβ deviation**	**Bond lengths**	**Bond angles**	**Cis Peptides**
			Avg: 29.18	Clashscore: 5	Outliers: 1 of 340	Poor rotamers: 0 of 287	Outliers: 0 of 322	Outliers: 0 of 347	Outliers: 0 of 347	Non-Trans: 1 of 345
A 21		THR	47.52	-	Favored (67.99%) General / -66.5,-27.7	Allowed (0.4%) *m* chi angles: 325.3	0.03Å	-	-	-
A 22		ASP	51.88	-	-	Allowed (0.4%) *p0* chi angles: 38.8,56.3	0.05Å	-	-	-
A 23		CSO	41.41	0.55Å OD with A 61 LEU O	-	-	0.06Å	-	-	-
A 24		LYS	52.53	-	-	Allowed (1.5%) *mmtp* chi angles: 321.1,279.3,201.5,103.9	0.03Å	-	-	-
A 25		SER	22.87	-	Favored (41.18%) General / -72.8,153.2	Favored (64.1%) *p* chi angles: 72.7	0.01Å	-	-	-
A 26		LEU	22.74	-	Favored (76.84%) General / -60.9,-35.5	Favored (70.6%) *mt* chi angles: 299.8,182.7	0.03Å	-	-	-
A 27		LEU	22.7	-	Favored (54.64%) General / -55.2,-53.5	Favored (48.1%) *tp* chi angles: 180,66.4	0.01Å	-	-	-
A 28		LYS	38.45	-	Favored (77.85%) General / -58.1,-39.1	Allowed (0.6%) *tmtm* chi angles: 203,244.7,176,303.2	0.02Å	-	-	-
A 29		LYS	36.68	0.43Å NZ with S 259 HOH O	Favored (73.06%) General / -63.5,-31.8	Favored (74.7%) *tttt* chi angles: 191,183.8,182.3,172.7	0.01Å	-	-	-
A 30		TYR	22.74	-	Favored (9.24%) General /	Favored (67.7%) *m-80* chi angles: 295.5,292.9	0.02Å	-	-	-

					-119.1,-15.2					
A 31		LEU	26.48	-	Favored (8.09%) General / -81.3,70.3	Favored (19%) *tp* chi angles: 190.1,69.1	0.04Å	-	-	-
A 32		ASN	32.42	-	Favored (7.72%) General / -81.1,179.8	Favored (33.3%) *p0* chi angles: 61.7,45.9	0.05Å	-	-	-
A 33		ARG	35.82	-	Favored (82.17%) General / -66.3,-36.1	Favored (4.1%) *tpt170* chi angles: 187.1,75.8,214.7,181.9	0.04Å	-	-	-
A 34		GLU	58.5	-	Favored (71.26%) General / -63.5,-50.0	Favored (3.3%) *mt-10* chi angles: 265,179.4,291.5	0.02Å	-	-	-
A 35		VAL	29.1	-	Favored (99.41%) Ile or Val / -61.8,-44.9	Favored (75.8%) *t* chi angles: 172.7	0.06Å	-	-	-
A 36		PHE	29.02	-	Favored (70.11%) General / -53.6,-45.4	Favored (80.8%) *t80* chi angles: 182.3,74	0.02Å	-	-	-
A 37		ASP	34	-	Favored (57.57%) General / -76.3,-30.3	Favored (50.9%) *m-30* chi angles: 286.6,319.7	0.04Å	-	-	-
A 38		GLN	35.45	-	Favored (69.93%) General / -59.3,-32.4	Favored (19%) *mt0* chi angles: 271.1,181.2,280.6	0.05Å	-	-	-
A 39		CYS	24.02	-	Favored (11.26%) General / -106.3,-27.0	Favored (61.5%) *m* chi angles: 300.7	0.01Å	-	-	-
A 40		LYS	31.47	-	Favored (68.98%) General / -63.6,-27.1	Favored (12.5%) *pttm* chi angles: 78.2,182.5,191.4,298.2	0.04Å	-	-	-
**#**	**Alt**	**Res**	**High B**	**Clash > 0.4Å**	**Ramachandran**	**Rotamer**	**Cβ deviation**	**Bond lengths**	**Bond angles**	**Cis Peptides**
			Avg: 29.18	Clashscore: 5	Outliers: 1 of 340	Poor rotamers: 0 of 287	Outliers: 0 of 322	Outliers: 0 of 347	Outliers: 0 of 347	Non-Trans: 1 of 345
A 41		SER	33.67	-	Favored (36.08%) General / -103.0,12.4	Favored (88.3%) *p* chi angles: 69.4	0.04Å	-	-	-
A 42		LEU	27.21	-	Favored (16.8%) General /	Favored (46.5%) *mt* chi angles: 305.4,173.7	0.01Å	-	-	-

				-91.8,157.6						
A 43	LYS	40.26	-	Favored (15.32%) General / -151.0,133.2	Allowed (0.8%) *tttt* chi angles: 203.3,209.8,233.2,192.4	0.04Å	-	-	-
A 44	THR	22.28	-	Favored (22.04%) General / -79.5,166.4	Favored (40.9%) *p* chi angles: 67.6	0.04Å	-	-	-
A 45	ALA	23.89	-	Favored (79.59%) General / -56.2,-44.3	-	0.01Å	-	-	-
A 46	LEU	23.16	-	Favored (26.85%) General / -72.1,-4.1	Favored (90.4%) *mt* chi angles: 292.5,168.4	0.02Å	-	-	-
A 47	GLY	23.13	-	Favored (43.98%) Glycine / 103.7,6.3	-	-	-	-	-
A 48	ALA	20.07	-	Favored (36%) General / -76.9,149.8	-	0.02Å	-	-	-
A 49	THR	21.8	0.76Å HG21 with A 6 THR HG21	Favored (20.1%) General / -120.3,162.1	Allowed (0.4%) *m* chi angles: 278.8	0.15Å	-	-	-
A 50	LEU	18.96	-	Favored (70.04%) General / -56.1,-37.8	Favored (59.3%) *tp* chi angles: 175.6,58.9	0.01Å	-	-	-
A 51	LEU	17.87	0.73Å H with A 49 THR HG22	Favored (73.96%) General / -59.3,-35.6	Favored (46%) *tp* chi angles: 183.9,64	0.01Å	-	-	-
A 52	ASP	18.44	-	Favored (58.69%) General / -66.8,-12.4	Favored (60%) *m-30* chi angles: 293.3,308.6	0.00Å	-	-	-
A 53	CYS	13.78	-	Favored (7.31%) General / -94.5,-45.7	Favored (60.9%) *m* chi angles: 301.8	0.02Å	-	-	-
A 54	ILE	20.55	-	Favored (9.92%) Ile or Val / -105.6,-6.1	Favored (8.4%) *pt* chi angles: 75.5,170.5	0.05Å	-	-	-
A 55	GLN	21.44	-	Favored (76.4%) General / -59.0,-37.2	Favored (6.2%) *pt0* chi angles: 53,195.7,344.4	0.02Å	-	-	-
A 56	SER	19.12	-	Favored (71.88%) General /	Favored (46.5%) *t* chi angles: 179.9	0.02Å	-	-	-

					-71.0,-35.1					
A 57		GLY	21.84	-	Favored (75.86%) Glycine / -73.6,-23.9	-	-	-	-	-
A 58		VAL	27.41	-	Favored (55.61%) Ile or Val / -72.0,-47.4	Favored (8.8%) *t* chi angles: 161.3	0.07Å	-	-	-
A 59		GLU	41.31	-	Favored (77.94%) General / -69.0,-36.5	Favored (55.3%) *mm-30* chi angles: 293.2,311.9,301.2	0.02Å	-	-	-
A 60		ASN	24.27	-	Favored (2.29%) General / -111.3,86.3	Favored (57.4%) *m-40* chi angles: 298.1,277.7	0.03Å	-	-	-

**#**	**Alt**	**Res**	**High B**	**Clash > 0.4Å**	**Ramachandran**	**Rotamer**	**Cβ deviation**	**Bond lengths**	**Bond angles**	**Cis Peptides**
			Avg: 29.18	Clashscore: 5	Outliers: 1 of 340	Poor rotamers: 0 of 287	Outliers: 0 of 322	Outliers: 0 of 347	Outliers: 0 of 347	Non-Trans: 1 of 345
A 61		LEU	24.92	0.55Å O with A 23 CSO OD	Favored (66.16%) General / -63.3,-21.3	Favored (78.7%) *mt* chi angles: 300.4,180.3	0.01Å	-	-	-
A 62		ASP	41.46	-	Favored (19.2%) General / -81.0,4.5	Favored (13.7%) *p0* chi angles: 51,348.4	0.03Å	-	-	-
A 63		SER	24.4	-	Favored (39.59%) General / -53.9,137.2	Favored (54.9%) *m* chi angles: 300.1	0.06Å	-	-	-
A 64		GLY	22.77	-	Favored (41.68%) Glycine / -69.7,-48.6	-	-	-	-	-
A 65		VAL	20.86	-	Allowed (0.76%) Ile or Val / -99.0,-69.4	Favored (33.5%) *t* chi angles: 185.1	0.05Å	-	-	-
A 66		GLY	17.72	-	Favored (55.13%) Glycine / 89.5,17.4	-	-	-	-	-
A 67		ILE	15.56	-	Favored (30.6%) Ile or Val / -136.4,145.4	Favored (12.6%) *tt* chi angles: 199,171	0.04Å	-	-	-
A 68		TYR	24.83	-	Favored (31.97%) General / -134.9,162.6	Favored (96.2%) *m-80* chi angles: 297.8,279.9	0.05Å	-	-	-

#	Alt	Res	High B	Clash > 0.4Å	Ramachandran	Rotamer	Cβ deviation	Bond lengths	Bond angles	Cis Peptides
A 69		ALA	15.75	-	Favored (42.37%) Pre-Pro / -102.1,123.8	-	0.02Å	-	-	-
A 70		PRO	18.76	-	Favored (5.25%) Trans-Pro / -84.0,-18.5	Favored (54%) *Cg_endo* chi angles: 25.7,324.1,31	0.02Å	-	-	-
A 71		ASP	18.85	-	Favored (8.12%) General / -160.0,-179.8	Favored (26.3%) *p0* chi angles: 61.2,29.7	0.06Å	-	-	-
A 72		ALA	18.05	-	Favored (82.53%) General / -67.7,-42.2	-	0.03Å	-	-	-
A 73		GLU	23.69	-	Favored (67.95%) General / -65.8,-27.0	Favored (40.4%) *tt0* chi angles: 181.5,194,337.4	0.03Å	-	-	-
A 74		ALA	14.04	-	Favored (63.42%) General / -55.3,-33.5	-	0.02Å	-	-	-
A 75		TYR	16.19	-	Favored (93.03%) General / -60.7,-40.6	Favored (11.9%) *m-80* chi angles: 273.5,298.7	0.03Å	-	-	-
A 76		THR	19.86	-	Favored (12.09%) General / -82.1,-47.8	Favored (85.4%) *m* chi angles: 301.6	0.04Å	-	-	-
A 77		LEU	17.94	-	Favored (98.5%) General / -61.4,-43.6	Favored (25.1%) *tp* chi angles: 188.9,59.1	0.02Å	-	-	-
A 78		PHE	19.17	-	Favored (6.55%) General / -98.8,25.5	Favored (83.9%) *m-80* chi angles: 299.6,284.8	0.04Å	-	-	-
A 79		ALA	15.76	-	Favored (5.57%) Pre-Pro / -46.8,-35.6	-	0.03Å	-	-	-
A 80		PRO	17.54	-	Favored (76.6%) Trans-Pro / -58.2,-26.4	Favored (49.1%) *Cg_exo* chi angles: 327.7,39.2,329.6	0.03Å	-	-	-
**#**	**Alt**	**Res**	**High B**	**Clash > 0.4Å**	**Ramachandran**	**Rotamer**	**Cβ deviation**	**Bond lengths**	**Bond angles**	**Cis Peptides**
			Avg: 29.18	Clashscore: 5	Outliers: 1 of 340	Poor rotamers: 0 of 287	Outliers: 0 of 322	Outliers: 0 of 347	Outliers: 0 of 347	Non-Trans: 1 of 345

A 81	ILE	16.22	-	Favored (6.86%) Ile or Val / -99.7,-38.6	Favored (6%) *tp* chi angles: 183.2,57.7	0.11Å	-	-	-
A 82	PHE	15.49	-	Favored (97.85%) General / -63.7,-42.5	Favored (25.8%) *m-80* chi angles: 278.3,291.4	0.01Å	-	-	-
A 83	ASN	15.39	-	Favored (87.84%) Pre-Pro / -58.6,-50.5	Favored (62.5%) *m-40* chi angles: 282.2,357.9	0.07Å	-	-	-
A 84	PRO	15.78	-	Favored (26.85%) Trans-Pro / -67.3,-34.5	Favored (51.2%) *Cg_endo* chi angles: 25.3,321.8,35.6	0.02Å	-	-	-
A 85	ILE	16.8	-	Favored (96.75%) Ile or Val / -63.1,-46.2	Favored (76.6%) *mt* chi angles: 291.9,174.5	0.07Å	-	-	-
A 86	ILE	17.63	-	Favored (93.4%) Ile or Val / -60.0,-43.8	Favored (37.1%) *mt* chi angles: 284.2,168.6	0.07Å	-	-	-
A 87	GLU	24.78	-	Favored (91.45%) General / -61.2,-46.2	Favored (21.4%) *tm-30* chi angles: 185.5,282.5,331.9	0.00Å	-	-	-
A 88	ASP	23.23	-	Favored (36.72%) General / -63.7,-54.4	Favored (30.1%) *t0* chi angles: 188.4,320.9	0.04Å	-	-	-
A 89	TYR	23.36	-	Favored (89.46%) General / -59.0,-45.9	Favored (88.7%) *t80* chi angles: 179.9,80.1	0.04Å	-	-	-
A 90	HIS	24.51	-	Favored (6.88%) General / -88.1,15.9	Favored (95.7%) *m-70* chi angles: 296.3,281	0.04Å	-	-	-
A 91	GLU	42.37	-	Allowed (0.2%) General / 48.5,73.8	Favored (68.9%) *mt-10* chi angles: 283.7,169.3,16.9	0.04Å	-	-	-
A 92	GLY	25.3	-	Favored (44.89%) Glycine / 104.2,3.4	-	-	-	-	-
A 93	PHE	22.73	-	Favored (8.03%) General / -84.7,91.8	Favored (41.5%) *t80* chi angles: 192.7,84.1	0.04Å	-	-	-
A 94	LYS	42.73	0.49Å HE2 with S 134 HOH O	Favored (58.07%) Pre-Pro /	Allowed (1.5%) *mttm* chi angles:	0.03Å	-	-	-

					-77.4,164.5	325.2,222.9,174.2,278.8				
A 95		PRO	20.05	-	Favored (81.46%) Trans-Pro / -55.7,-31.8	Favored (99%) *Cg_exo* chi angles: 332.3,35.2,332	0.02Å	-	-	-
A 96		THR	25.16	-	Favored (58.16%) General / -89.8,-2.1	Favored (47.1%) *p* chi angles: 55.7	0.04Å	-	-	-
A 97		ASP	21.06	0.57Å OD1 with S 243 HOH O	Favored (32.04%) General / -74.6,159.5	Favored (66.4%) *m-30* chi angles: 279.9,345	0.01Å	-	-	-
A 98		LYS	33.13	-	Favored (37.72%) General / -136.8,135.2	Favored (70.9%) *tttt* chi angles: 183.1,179.9,164.9,181.7	0.04Å	-	-	-
A 99		HIS	19.07	-	Favored (45.26%) Pre-Pro / -82.2,139.2	Favored (83%) *t70* chi angles: 182.5,78.6	0.03Å	-	-	-
A 100		PRO	19.52	-	Favored (34.41%) Trans-Pro / -68.5,164.1	Favored (36.5%) *Cg_endo* chi angles: 22.6,323.9,34.8	0.04Å	-	-	-
**#**	**Alt**	**Res**	**High B**	**Clash > 0.4Å**	**Ramachandran**	**Rotamer**	**Cβ deviation**	**Bond lengths**	**Bond angles**	**Cis Peptides**
			Avg: 29.18	Clashscore: 5	Outliers: 1 of 340	Poor rotamers: 0 of 287	Outliers: 0 of 322	Outliers: 0 of 347	Outliers: 0 of 347	Non-Trans: 1 of 345
A 101		PRO	23.66	-	Favored (76.08%) Trans-Pro / -65.1,153.9	Favored (8.8%) *Cg_exo* chi angles: 347.8,25.3,332.4	0.03Å	-	-	-
A 102		THR	22.28	-	Favored (35.63%) General / -54.0,129.8	Favored (88.8%) *m* chi angles: 298.4	0.06Å	-	-	-
A 103		ASP	27.09	-	Favored (3.03%) General / -152.7,106.0	Favored (20.6%) *t0* chi angles: 177.7,30.1	0.01Å	-	-	-
A 104		PHE	25.19	-	Favored (56.83%) General / -77.5,-7.8	Favored (71.3%) *m-80* chi angles: 298.7,291.6	0.01Å	-	-	-
A 105		GLY	23.67	-	Favored (50.52%) Glycine / 71.9,-169.6	-	-	-	-	-
A 106		ASP	37.86	-	Favored (13.43%) General / -90.2,100.6	Favored (61.9%) *t0* chi angles: 182.5,2.7	0.04Å	-	-	-

A 107	ILE	40.65	0.43Å HG12 with A 244 GLN HB2	Favored (19.12%) Ile or Val / -56.2,-25.1	Favored (4.4%) *tp* chi angles: 188.3,52.8	0.08Å	-	-	-
A 108	ASN	48.44	-	Favored (59.22%) General / -72.5,-10.0	Favored (78.9%) *m-40* chi angles: 283.4,330.1	0.01Å	-	-	-
A 109	THR	27.18	-	Favored (57.56%) General / -89.9,-4.3	Favored (71.4%) *p* chi angles: 61.9	0.03Å	-	-	-
A 110	ILE	23.84	-	Favored (19.08%) Ile or Val / -90.6,138.6	Favored (35.3%) *mm* chi angles: 306.7,296.6	0.05Å	-	-	-
A 111	VAL	22.51	-	Favored (19.59%) Ile or Val / -125.2,167.3	Favored (11.4%) *m* chi angles: 306.4	0.02Å	-	-	-
A 112	ASN	27.38	-	Favored (10.17%) General / -69.8,116.4	Favored (48.2%) *t0* chi angles: 188.3,318.3	0.07Å	-	-	-
A 113	VAL	23.06	-	Favored (17.99%) Ile or Val / -72.5,-16.1	Favored (13.6%) *m* chi angles: 291.4	0.03Å	-	-	-
A 114	ASP	24.46	-	Favored (5.84%) Pre-Pro / -152.4,80.3	Favored (38.2%) *t0* chi angles: 180,19.1	0.00Å	-	-	-
A 115	PRO	28.58	-	Favored (98.24%) Trans-Pro / -58.4,-33.9	Favored (85.2%) *Cg_exo* chi angles: 334,33.4,333.1	0.02Å	-	-	-
A 116	SER	33.17	-	Favored (59.89%) General / -78.1,-11.1	Favored (82.6%) *p* chi angles: 62.3	0.02Å	-	-	-
A 117	GLY	26.79	-	Favored (69.54%) Glycine / 64.3,25.0	-	-	-	-	-
A 118	LYS	29.1	-	Favored (46.66%) General / -78.4,-28.2	Favored (46%) *tttt* chi angles: 200.2,178.4,167.8,172.2	0.07Å	-	-	-
A 119	TYR	29.11	0.46Å CE1 with A 353 GLU HG2	Allowed (1.59%) General / -126.1,-48.6	Favored (80%) *m-80* chi angles: 300.1,286.3	0.03Å	-	-	-
A 120	VAL	21.88	-	Favored (30.16%)	Favored (73%) *t* chi angles: 172.5	0.05Å	-	-	-

#	Alt	Res	High B	Clash > 0.4Å	Ramachandran	Rotamer	Cβ deviation	Bond lengths	Bond angles	Cis Peptides
					Ile or Val / -83.2,118.0					
			Avg: 29.18	Clashscore: 5	Outliers: 1 of 340	Poor rotamers: 0 of 287	Outliers: 0 of 322	Outliers: 0 of 347	Outliers: 0 of 347	Non-Trans: 1 of 345
A 121		VAL	23.24	-	Favored (45.43%) Ile or Val / -74.9,-43.8	Favored (85.7%) *t* chi angles: 177.4	0.06Å	-	-	-
A 122		SER	22.83	-	Favored (26.15%) General / -161.1,157.5	Favored (62.7%) *p* chi angles: 58.1	0.03Å	-	-	-
A 123		THR	20.44	-	Favored (50.95%) General / -126.8,144.4	Favored (91.3%) *m* chi angles: 301.1	0.05Å	-	-	-
A 124		HIS	29.22	0.56Å ND1 with S 166 HOH O	Favored (41.03%) General / -135.8,136.8	Favored (22.9%) *m90* chi angles: 278.4,66.3	0.08Å	-	-	-
A 125		VAL	20.3	-	Favored (68.1%) Ile or Val / -128.4,128.2	Favored (13.7%) *t* chi angles: 188.6	0.05Å	-	-	-
A 126		ARG	46.29	-	Favored (50.86%) General / -128.9,142.7	Allowed (0.5%) *ttt180* chi angles: 154.9,182.5,126.7,197.6	0.02Å	-	-	-
A 127		CYS	17.64	-	Favored (9.66%) General / -138.3,175.3	Favored (27.9%) *p* chi angles: 67.5	0.07Å	-	-	-
A 128		GLY	14.89	-	Favored (21.53%) Glycine / -138.2,151.7	-	-	-	-	-
A 129		ARG	15.9	-	Favored (27.85%) General / -145.3,140.5	Favored (81.1%) *mmt-90* chi angles: 293.1,295.4,190.8,264.2	0.04Å	-	-	-
A 130		SER	17.38	-	Favored (37.66%) General / -105.5,141.1	Favored (58.9%) *m* chi angles: 299.3	0.03Å	-	-	-
A 131		LEU	18.66	-	Favored (32.06%) General / -84.6,125.0	Favored (13.5%) *mt* chi angles: 307.4,190.7	0.04Å	-	-	-

A 132		LYS	39.64	-	Favored (50.66%) General / -55.7,136.3	Favored (2.8%) *tttp* chi angles: 182.2,196.5,212.7,62.2	0.02Å	-	-	-
A 133		GLY	21.9	-	Favored (59.52%) Glycine / 97.8,-17.0	-	-	-	-	-
A 134		TYR	31.19	-	Favored (32.14%) Pre-Pro / -104.0,146.2	Favored (93.6%) *m-80* chi angles: 291.9,89.2	0.06Å	-	-	-
A 135		PRO	17.54	-	Favored (4.67%) Trans-Pro / -51.7,158.1	Favored (13.5%) *Cg_exo* chi angles: 345.2,21.2,341.4	0.05Å	-	-	-
A 136		PHE	17.83	-	Favored (8.59%) General / -82.9,177.5	Favored (5.6%) *m-10* chi angles: 296.2,359.6	0.04Å	-	-	-
A 137		ASN	21.19	0.41Å HA with A 140 LEU HD12	Favored (3.99%) Pre-Pro / -60.4,-28.1	Favored (2.1%) *m-40* chi angles: 263,271.2	0.07Å	-	-	-
A 138		PRO	17.9	-	Favored (47.54%) Trans-Pro / -56.0,-24.2	Favored (94.6%) *Cg_exo* chi angles: 331.4,35.8,331.4	0.04Å	-	-	-
A 139		CYS	19.46	-	Favored (4.68%) General / -131.2,8.1	Favored (62.9%) *m* chi angles: 286	0.02Å	-	-	-
A 140		LEU	20.42	0.41Å HD12 with A 137 ASN HA	Favored (58.22%) General / -66.0,139.1	Favored (15.8%) *mt* chi angles: 280.7,159.3	0.01Å	-	-	-
**#**	**Alt**	**Res**	**High B**	**Clash > 0.4Å**	**Ramachandran**	**Rotamer**	**Cβ deviation**	**Bond lengths**	**Bond angles**	**Cis Peptides**
			Avg: 29.18	Clashscore: 5	Outliers: 1 of 340	Poor rotamers: 0 of 287	Outliers: 0 of 322	Outliers: 0 of 347	Outliers: 0 of 347	Non-Trans: 1 of 345
A 141		THR	20.7	0.45Å H with A 144 ASN HD22	Favored (16.64%) General / -86.9,164.3	Favored (50.3%) *p* chi angles: 65.6	0.02Å	-	-	-
A 142		GLU	36.54	-	Favored (75.7%) General / -55.2,-45.8	Favored (65.4%) *tt0* chi angles: 179.1,177.3,24.8	0.01Å	-	-	-
A 143		ALA	19.72	-	Favored (95.76%) General / -61.3,-40.9	-	0.02Å	-	-	-

A 144	ASN	21.01	0.45Å HD22 with A 141 THR H	Favored (72.15%) General / -54.8,-48.9	Favored (84.2%) *m-40* chi angles: 283.3,338.4	0.02Å	-	-	-
A 145	TYR	19.41	-	Favored (86.59%) General / -57.8,-45.2	Favored (11.2%) *m-80* chi angles: 274.1,301.1	0.01Å	-	-	-
A 146	LYS	38.34	-	Favored (76.88%) General / -66.0,-46.7	Favored (85.4%) *mttt* chi angles: 299.3,179.6,176.3,168.8	0.02Å	-	-	-
A 147	GLU	39.51	-	Favored (94.72%) General / -65.0,-40.3	Favored (8.9%) *tt0* chi angles: 170.4,200,210.1	0.02Å	-	-	-
A 148	MET	22.43	-	Favored (91.41%) General / -62.0,-45.9	Favored (46.4%) *ttm* chi angles: 180.6,182.3,299.7	0.01Å	-	-	-
A 149	GLU	26.91	0.41Å O with A 153 SER HB3	Favored (85.65%) General / -58.5,-41.5	Favored (39.1%) *tt0* chi angles: 193.3,180.6,26.5	0.02Å	-	-	-
A 150	ASP	40.34	-	Favored (50.48%) General / -55.8,-54.0	Favored (14.1%) *t0* chi angles: 205.5,9.9	0.03Å	-	-	-
A 151	LYS	42.96	-	Favored (82.75%) General / -61.9,-47.9	Allowed (2%) *mttt* chi angles: 257.5,149,182.2,162.7	0.01Å	-	-	-
A 152	VAL	22.85	-	Favored (74.63%) Ile or Val / -69.9,-46.1	Favored (16.4%) *t* chi angles: 163.8	0.09Å	-	-	-
A 153	SER	35.77	0.41Å HB3 with A 149 GLU O	Favored (74.71%) General / -63.0,-33.1	Allowed (0.3%) *t* chi angles: 147.8	0.03Å	-	-	-
A 154	ALA	29.19	-	Favored (76.68%) General / -60.5,-49.7	-	0.03Å	-	-	-
A 155	ILE	37.41	-	Favored (80.38%) Ile or Val / -56.0,-46.2	Favored (4.2%) *mm* chi angles: 301.5,319	0.04Å	-	-	-
A 156	PHE	27.08	-	Favored (73.12%) General / -57.2,-38.0	Favored (14.7%) *m-10* chi angles: 286.5,350.8	0.02Å	-	-	-

#	Alt	Res	High B	Clash > 0.4Å	Ramachandran	Rotamer	Cβ deviation	Bond lengths	Bond angles	Cis Peptides
A 157		GLY	34.34	-	Favored (60.71%) Glycine / -66.4,-13.6	-	-	-	-	-
A 158		THR	35.24	-	Favored (42.73%) General / -96.7,-3.4	Favored (4.2%) *p* chi angles: 79.7	0.03Å	-	-	-
A 159		PHE	30.93	-	Favored (26.43%) General / -60.8,126.2	Favored (3.8%) *m-10* chi angles: 280.3,340.4	0.04Å	-	-	-
A 160		GLU	48.63	0.65Å O with S 222 HOH O	Favored (3.62%) General / -109.4,-178.7	Favored (29.4%) *mt-10* chi angles: 301.9,172.1,251.7	0.01Å	-	-	-
**#**	**Alt**	**Res**	**High B**	**Clash > 0.4Å**	**Ramachandran**	**Rotamer**	**Cβ deviation**	**Bond lengths**	**Bond angles**	**Cis Peptides**
			Avg: 29.18	Clashscore: 5	Outliers: 1 of 340	Poor rotamers: 0 of 287	Outliers: 0 of 322	Outliers: 0 of 347	Outliers: 0 of 347	Non-Trans: 1 of 345
A 161		GLY	33.18	-	Favored (48.03%) Glycine / 60.6,-140.8	-	-	-	-	-
A 162		GLU	34.75	-	Favored (65.92%) General / -63.3,-20.9	Favored (17.1%) *pt0* chi angles: 68.7,173.6,346	0.02Å	-	-	-
A 163		LEU	27.92	-	Favored (56.62%) General / -87.5,-8.3	Favored (50.8%) *mt* chi angles: 296.2,184.6	0.01Å	-	-	-
A 164		LYS	45.76	-	Favored (57.84%) General / -60.5,139.3	Allowed (1.6%) *tttm* chi angles: 202.6,197,165.7,251.6	0.02Å	-	-	-
A 165		GLY	22.88	0.42Å HA3 with A 213 ASN HA	Favored (3.98%) Glycine / -179.3,-140.3	-	-	-	-	-
A 166		LYS	41.5	-	Favored (23.98%) General / -145.8,136.1	Favored (14%) *tttt* chi angles: 185.5,166.5,155,154.3	0.02Å	-	-	-
A 167		TYR	19.14	-	Favored (33.64%) General / -88.4,135.1	Favored (68.5%) *t80* chi angles: 181.2,67.8	0.02Å	-	-	-
A 168		TYR	28.09	-	Favored (43.98%) Pre-Pro / -119.1,106.0	Favored (34.9%) *m-80* chi angles: 304.7,79.9	0.06Å	-	-	-

A 169		PRO	18.45	-	Favored (82.6%) Trans-Pro / -64.3,143.3	Favored (2.7%) *Cg_exo* chi angles: 356.8,17,336.5	0.04Å	-	-	-
A 170		LEU	17.67	-	Favored (63.06%) General / -72.0,-28.0	Favored (3.7%) *mp* chi angles: 262.3,54.4	0.02Å	-	-	-
A 171		THR	18.21	-	Favored (20.27%) General / -67.5,123.4	Favored (84.9%) *m* chi angles: 301.7	0.03Å	-	-	-
A 172		GLY	22.18	-	Favored (75.78%) Glycine / 71.9,14.0	-	-	-	-	-
A 173		MET	18.79	-	Favored (48.53%) General / -69.4,134.0	Favored (62.6%) *ttm* chi angles: 185.7,181.4,287.6	0.02Å	-	-	-
A 174		ASP	18.22	-	Favored (14.22%) General / -92.3,162.6	Favored (3.1%) *p0* chi angles: 65.8,63.2	0.03Å	-	-	-
A 175		LYS	24.23	-	Favored (72.93%) General / -65.0,-31.6	Allowed (0.6%) *tttp* chi angles: 207.2,129.5,160.2,93.3	0.02Å	-	-	-
A 176		ALA	18.4	-	Favored (71.84%) General / -69.9,-43.3	-	0.02Å	-	-	-
A 177		THR	19.48	-	Favored (91.21%) General / -58.9,-43.7	Favored (65.9%) *m* chi angles: 303.2	0.05Å	-	-	-
A 178		GLN	31.69	-	Favored (67.43%) General / -54.3,-50.4	Allowed (0.4%) *tt0* chi angles: 231.4,158.8,13.8	0.03Å	-	-	-
A 179		GLN	28.4	-	Favored (95.06%) General / -62.7,-44.9	Favored (66.6%) *tp40* chi angles: 194.7,55.4,56.5	0.02Å	-	-	-
A 180		GLN	36.78	-	Favored (74.25%) General / -62.9,-32.9	Favored (4.8%) *pt0* chi angles: 67.1,174.8,251.5	0.03Å	-	-	-
**#**	**Alt**	**Res**	**High B**	**Clash > 0.4Å**	**Ramachandran**	**Rotamer**	**Cβ deviation**	**Bond lengths**	**Bond angles**	**Cis Peptides**
			Avg: 29.18	Clashscore: 5	Outliers: 1 of 340	Poor rotamers: 0 of 287	Outliers: 0 of 322	Outliers: 0 of 347	Outliers: 0 of 347	Non-Trans: 1 of 345

A 181	LEU	19.19	-	Favored (60.14%) General / -74.2,-42.1	Favored (79.8%) *mt* chi angles: 288.8,170.6	0.01Å	-	-	-
A 182	ILE	23.19	-	Favored (71.84%) Ile or Val / -54.5,-47.0	Favored (68.8%) *mt* chi angles: 289.6,162.5	0.04Å	-	-	-
A 183	ASP	30.5	0.78Å OD2 with S 191 HOH O	Favored (66.77%) General / -56.5,-34.3	Favored (91.3%) *m-30* chi angles: 287.6,353.3	0.02Å	-	-	-
A 184	ASP	32.16	-	Favored (59.2%) General / -84.2,-5.1	Favored (68.1%) *m-30* chi angles: 290.4,321.6	0.02Å	-	-	-
A 185	HIS	35.6	-	Favored (12.66%) General / 62.7,37.7	Favored (55%) *m-70* chi angles: 307.4,268.9	0.06Å	-	-	-
A 186	PHE	21.18	-	Favored (15.48%) General / -107.2,-12.0	Favored (17.2%) *m-10* chi angles: 288.8,351	0.03Å	-	-	-
A 187	LEU	18.13	-	Favored (27.04%) General / -86.2,145.4	Favored (8.8%) *tp* chi angles: 193.4,77.1	0.03Å	-	-	-
A 188	PHE	18.26	-	Favored (34.42%) General / -120.5,155.0	Favored (55.7%) *p90* chi angles: 65,94.1	0.05Å	-	-	-
A 189	LYS	43.15	-	Favored (2.35%) General / -112.9,-173.9	Favored (4.5%) *pttm* chi angles: 51.2,205.6,177.9,309.2	0.04Å	-	-	-
A 190	GLU	36.48	-	Favored (41.79%) General / -58.1,143.9	Allowed (0.3%) *mt-10* chi angles: 241,203.4,304	0.02Å	-	-	-
A 191	GLY	22.13	-	Favored (53.18%) Glycine / -69.8,159.8	-	-	-	-	-
A 192	ASP	26.76	-	Favored (5.21%) General / -75.8,179.9	Favored (47.3%) *p0* chi angles: 61.4,17.6	0.03Å	-	-	-
A 193	ARG	24.3	-	Favored (63.24%) General / -56.4,-30.8	Favored (39.1%) *ptt90* chi angles: 59.7,187.5,166.6,87.8	0.02Å	-	-	-
A 194	PHE	27.91	-	Favored (82.78%)	Favored (93.3%) *m-80*	0.02Å	-	-	-

#	Alt	Res	High B	Clash > 0.4Å	Ramachandran	Rotamer	Cβ deviation	Bond lengths	Bond angles	Cis Peptides
					General / -66.5,-44.4	chi angles: 298.9,280				
A 195		LEU	24.28	-	Favored (80.82%) General / -68.7,-38.9	Favored (36.3%) *mt* chi angles: 293,184.9	0.01Å	-	-	-
A 196		GLN	26.01	-	Favored (81.47%) General / -59.8,-48.5	Favored (47.9%) *mm-40* chi angles: 299.8,280.5,335.4	0.01Å	-	-	-
A 197		ALA	19.87	-	Favored (64.34%) General / -68.7,-19.3	-	0.03Å	-	-	-
A 198		ALA	15.76	-	Favored (36.38%) General / -83.7,2.7	-	0.01Å	-	-	-
A 199		ASN	24.14	-	Favored (15.86%) General / 63.0,27.3	Favored (50.2%) *m-40* chi angles: 277.4,351.4	0.04Å	-	-	-
A 200		ALA	19.31	-	Favored (49.14%) General / -91.5,5.5	-	0.02Å	-	-	-
**#**	**Alt**	**Res**	**High B**	**Clash > 0.4Å**	**Ramachandran**	**Rotamer**	**Cβ deviation**	**Bond lengths**	**Bond angles**	**Cis Peptides**
			Avg: 29.18	Clashscore: 5	Outliers: 1 of 340	Poor rotamers: 0 of 287	Outliers: 0 of 322	Outliers: 0 of 347	Outliers: 0 of 347	Non-Trans: 1 of 345
A 201		CYS	24.02	-	Favored (9.42%) General / -123.6,20.5	Favored (55.5%) *m* chi angles: 302.4	0.04Å	-	-	-
A 202		ARG	23.37	-	Favored (43.46%) General / -72.0,151.2	Favored (24.3%) *mtm110* chi angles: 299.3,165.5,298.3,103.8	0.02Å	-	-	-
A 203		TYR	32.19	-	Favored (13.48%) General / 62.8,22.6	Favored (65.7%) *m-80* chi angles: 299.9,293.2	0.04Å	-	-	-
A 204		TRP	23.94	-	Favored (90.32%) Pre-Pro / -60.3,132.4	Favored (32.8%) *t-100* chi angles: 190.2,274.7	0.04Å	-	-	-
A 205		PRO	16.9	-	Favored (10.46%) Cis-Pro / -104.9,24.2	Favored (17.3%) *Cg_endo* chi angles: 37.3,328.9,12.6	0.04Å	-	-	Cis PRO omega= 4.29
A 206		THR	22.08	-	Favored (22.08%) General / -53.8,126.4	Favored (15.7%) *m* chi angles: 310	0.05Å	-	-	-

A 207	GLY	14.53	-	Favored (89.65%) Glycine / 80.5,2.6	-	-	-	-	-
A 208	ARG	16.94	-	Favored (34.66%) General / -102.7,141.9	Favored (2.2%) *mpt180* chi angles: 280.7,65.2,160.7,216.6	0.02Å	-	-	-
A 209	GLY	12.59	-	Favored (45.32%) Glycine / -173.5,172.6	-	-	-	-	-
A 210	ILE	18.01	-	Favored (48.93%) Ile or Val / -136.2,131.3	Favored (6.3%) *mt* chi angles: 292.8,147.8	0.04Å	-	-	-
A 211	TYR	25.11	-	Favored (50.45%) General / -120.3,141.0	Favored (60.4%) *t80* chi angles: 180,272.1	0.02Å	-	-	-
A 212	HIS	22.38	-	Favored (42.13%) General / -148.1,156.0	Favored (16.2%) *p90* chi angles: 73.3,74	0.09Å	-	-	-
A 213	ASN	22.6	0.42Å HA with A 165 GLY HA3	Favored (15.8%) General / -89.0,162.9	Favored (41.9%) *p0* chi angles: 65.4,343.2	0.02Å	-	-	-
A 214	ASP	38.46	-	Favored (62.36%) General / -52.3,-40.8	Favored (46.9%) *m-30* chi angles: 303.3,332.4	0.01Å	-	-	-
A 215	ALA	24.85	-	Favored (50.05%) General / -75.7,-6.3	-	0.02Å	-	-	-
A 216	LYS	29.27	-	Favored (29.23%) General / 56.3,41.3	Favored (91.7%) *mttt* chi angles: 301.3,185,180.8,175.6	0.06Å	-	-	-
A 217	THR	22.85	-	Favored (15.27%) General / -116.8,14.4	Favored (66.4%) *p* chi angles: 58.4	0.04Å	-	-	-
A 218	PHE	21.4	-	Favored (5.88%) General / -156.5,125.2	Favored (68.9%) *t80* chi angles: 174,68.9	0.02Å	-	-	-
A 219	LEU	16.76	-	Favored (41.26%) General / -136.6,138.5	Favored (12.7%) *tp* chi angles: 169.8,73.3	0.03Å	-	-	-
A 220	VAL	15.33	-	Favored (70.26%)	Favored (36.5%) *t* chi angles: 184.2	0.03Å	-	-	-

#	Alt	Res	High B	Clash > 0.4Å	Ramachandran	Rotamer	Cβ deviation	Bond lengths	Bond angles	Cis Peptides
					Ile or Val / -127.0,132.1					
			Avg: 29.18	Clashscore: 5	Outliers: 1 of 340	Poor rotamers: 0 of 287	Outliers: 0 of 322	Outliers: 0 of 347	Outliers: 0 of 347	Non-Trans: 1 of 345
A 221		TRP	18.28	-	Favored (49.97%) General / -102.7,126.8	Favored (42.9%) *m-10* chi angles: 286.3,354.2	0.02Å	-	-	-
A 222		VAL	14.61	-	Favored (45.61%) Ile or Val / -99.9,117.8	Favored (34.6%) *t* chi angles: 184.7	0.04Å	-	-	-
A 223		ASN	16.08	-	Favored (18.01%) General / 61.9,31.9	Favored (64.7%) *m-40* chi angles: 283.2,316.8	0.08Å	-	-	-
A 224		GLU	24.92	-	Favored (67.35%) General / -72.9,-36.4	Favored (12.4%) *tm-30* chi angles: 182.8,266.5,335.9	0.02Å	-	-	-
A 225		GLU	32.32	0.54Å HG3 with A 270 PHE CD2	Allowed (0.11%) General / -154.8,-58.3	Favored (2.9%) *tt0* chi angles: 156.7,163.4,341.4	0.02Å	-	-	-
A 226		ASP	16.26	-	Favored (17.59%) General / -87.0,162.5	Favored (16.3%) *m-30* chi angles: 281.9,309.1	0.04Å	-	-	-
A 227		HIS	13.93	-	Favored (42.34%) General / -49.6,-50.4	Favored (43.8%) *m170* chi angles: 296.8,152.6	0.01Å	-	-	-
A 228		LEU	13.8	-	Favored (44.98%) General / -129.3,129.6	Favored (78.5%) *mt* chi angles: 301.7,176.2	0.03Å	-	-	-
A 229		ARG	41.52	0.76Å NH1 with S 231 HOH O	Favored (26.55%) General / -113.2,114.3	Favored (6.5%) *ttm-80* chi angles: 184.6,198.5,291.5,241	0.01Å	-	-	-
A 230		ILE	19.74	-	Favored (68.16%) Ile or Val / -111.6,124.2	Favored (11.2%) *mt* chi angles: 308.5,157.1	0.06Å	-	-	-
A 231		ILE	25.98	-	Favored (50.8%) Ile or Val / -129.9,139.7	Allowed (0.7%) *mt* chi angles: 312.4,138.4	0.01Å	-	-	-

#	Alt	Res	High B	Clash > 0.4Å	Ramachandran	Rotamer	Cβ deviation	Bond lengths	Bond angles	Cis Peptides
A 232		SER	19.29	-	Favored (46.09%) General / -129.8,131.2	Favored (46.5%) *t* chi angles: 179.8	0.03Å	-	-	-
A 233		MET	20.09	-	Favored (41.32%) General / -154.5,162.1	Allowed (0.4%) *pmt* chi angles: 68.7,296.4,187.8	0.01Å	-	-	-
A 234		GLN	22.11	0.59Å HG2 with A 242 ILE HD11	Favored (7.32%) General / -171.1,163.9	Favored (18.6%) *pt0* chi angles: 60.2,187.9,51.9	0.05Å	-	-	-
A 235		LYS	43.89	-	Favored (38.62%) General / -60.0,147.6	Favored (4.9%) *mmtm* chi angles: 300.5,291.4,214.9,318.3	0.04Å	-	-	-
A 236		GLY	19.41	-	Favored (36.67%) Glycine / 84.2,-160.4	-	-	-	-	-
A 237		GLY	19.55	-	Favored (4.26%) Glycine / -130.2,25.9	-	-	-	-	-
A 238		ASP	26.83	-	Favored (3.97%) General / -77.4,75.5	Favored (49.9%) *t0* chi angles: 187.8,2.5	0.05Å	-	-	-
A 239		LEU	22.63	-	Favored (62.73%) General / -55.0,-33.8	Favored (32.5%) *tp* chi angles: 186.7,58	0.02Å	-	-	-
A 240		LYS	38.93	-	Favored (5.34%) General / -61.2,-59.4	Favored (33.2%) *tttt* chi angles: 170.5,166.3,194,162.8	0.00Å	-	-	-
#	Alt	Res	High B	Clash > 0.4Å	Ramachandran	Rotamer	Cβ deviation	Bond lengths	Bond angles	Cis Peptides
			Avg: 29.18	Clashscore: 5	Outliers: 1 of 340	Poor rotamers: 0 of 287	Outliers: 0 of 322	Outliers: 0 of 347	Outliers: 0 of 347	Non-Trans: 1 of 345
A 241		THR	26.7	-	Favored (74.76%) General / -57.1,-39.4	Favored (81.6%) *m* chi angles: 296.5	0.05Å	-	-	-
A 242		ILE	21.78	0.59Å HD11 with A 234 GLN HG2	Favored (75.1%) Ile or Val / -65.5,-49.9	Favored (7.6%) *mt* chi angles: 283.9,153	0.07Å	-	-	-
A 243		PHE	21.66	-	Favored (90.56%) General / -65.3,-43.6	Favored (10.3%) *t80* chi angles: 184.3,38.5	0.03Å	-	-	-

A 244	GLN	40.16	0.43Å HB2 with A 107 ILE HG12	Favored (93.31%) General / -60.3,-41.5	Favored (25.9%) *tp40* chi angles: 179.3,70.7,86.1	0.02Å	-	-	-
A 245	ARG	27.04	-	Favored (79.48%) General / -60.6,-36.8	Favored (12.9%) *ttm110* chi angles: 171.4,188.2,316,129.9	0.01Å	-	-	-
A 246	LEU	21.8	-	Favored (69.02%) General / -68.1,-47.1	Favored (31.7%) *tp* chi angles: 181.3,69.7	0.02Å	-	-	-
A 247	VAL	27.91	-	Favored (75.19%) Ile or Val / -66.4,-35.6	Allowed (1.8%) *p* chi angles: 53.4	0.11Å	-	-	-
A 248	ASN	35.81	-	Favored (89.46%) General / -58.5,-44.6	Favored (80%) *m-40* chi angles: 289.7,352.2	0.02Å	-	-	-
A 249	ALA	24.42	-	Favored (73.6%) General / -64.6,-48.8	-	0.03Å	-	-	-
A 250	VAL	22.8	-	Favored (82.84%) Ile or Val / -57.0,-47.7	Favored (60.1%) *t* chi angles: 170.8	0.06Å	-	-	-
A 251	ASN	25.21	-	Favored (91.76%) General / -61.1,-46.1	Favored (99%) *m-40* chi angles: 287.6,337.9	0.01Å	-	-	-
A 252	THR	27.64	-	Favored (61.92%) General / -53.0,-51.3	Favored (32%) *m* chi angles: 306.5	0.04Å	-	-	-
A 253	ILE	22.89	-	Favored (93.3%) Ile or Val / -60.9,-47.5	Favored (71%) *mt* chi angles: 299.1,164.4	0.05Å	-	-	-
A 254	GLU	27.27	0.50Å OE2 with S 21 HOH O	Favored (74.56%) General / -56.8,-39.6	Favored (38.7%) *tt0* chi angles: 180.7,168.2,221.9	0.02Å	-	-	-
A 255	SER	36.84	-	Favored (24.26%) General / -52.9,-28.6	Allowed (1.2%) *p* chi angles: 91.3	0.04Å	-	-	-
A 256	LYS	41.6	-	Favored (15.54%) General / -107.6,-10.9	Favored (11%) *mmtp* chi angles: 305.6,279.7,168.8,80.1	0.01Å	-	-	-

#	Alt	Res	High B	Clash > 0.4Å	Ramachandran	Rotamer	Cβ deviation	Bond lengths	Bond angles	Cis Peptides
A 257		LEU	35.32	-	Favored (2.3%) Pre-Pro / -154.9,104.6	Allowed (0.9%) *tp* chi angles: 221.3,42	0.03Å	-	-	-
A 258		PRO	27.87	-	Favored (59.9%) Trans-Pro / -70.5,148.4	Favored (5.2%) *Cg_exo* chi angles: 350.8,25.1,329.5	0.04Å	-	-	-
A 259		PHE	20.11	-	Favored (19.06%) General / -96.6,152.0	Favored (23.2%) *m-10* chi angles: 301.4,321.6	0.02Å	-	-	-
A 260		SER	25.25	-	Favored (20.57%) General / -81.2,115.3	Favored (9.6%) *t* chi angles: 166	0.06Å	-	-	-
#	Alt	Res	High B	Clash > 0.4Å	Ramachandran	Rotamer	Cβ deviation	Bond lengths	Bond angles	Cis Peptides
			Avg: 29.18	Clashscore: 5	Outliers: 1 of 340	Poor rotamers: 0 of 287	Outliers: 0 of 322	Outliers: 0 of 347	Outliers: 0 of 347	Non-Trans: 1 of 345
A 261		ARG	17.62	-	Favored (42.57%) General / -136.7,140.3	Favored (43%) *ttt-90* chi angles: 196.5,187.3,191.2,279.5	0.04Å	-	-	-
A 262		ASP	21.19	-	Favored (49.56%) General / -113.7,138.9	Favored (30.1%) *t70* chi angles: 180.2,76.7	0.03Å	-	-	-
A 263		ASP	33	-	Favored (82.88%) General / -57.2,-43.7	Favored (88%) *m-30* chi angles: 285.5,341.5	0.01Å	-	-	-
A 264		ARG	15.76	-	Favored (26.99%) General / -80.8,-39.2	Favored (4.9%) *ttp80* chi angles: 187.1,176,103.5,76.7	0.01Å	-	-	-
A 265		LEU	15.42	-	Favored (20.51%) General / -102.3,-9.3	Favored (40.1%) *mt* chi angles: 296.2,186.1	0.02Å	-	-	-
A 266		GLY	14.59	-	Favored (33.14%) Glycine / 79.0,-149.8	-	-	-	-	-
A 267		PHE	15.76	-	Favored (40.55%) General / -70.1,129.9	Favored (79.2%) *m-80* chi angles: 290.4,281.4	0.02Å	-	-	-
A 268		LEU	16.67	-	Favored (34.37%) General / -86.6,130.8	Favored (56.6%) *mt* chi angles: 297.4,184.5	0.03Å	-	-	-

#	Alt	Res	High B	Clash > 0.4Å	Ramachandran	Rotamer	Cβ deviation	Bond lengths	Bond angles	Cis Peptides
A 269		THR	14.29	-	Favored (28.06%) General / -127.0,161.6	Favored (41.3%) *p* chi angles: 54.8	0.04Å	-	-	-
A 270		PHE	23.12	0.54Å CD2 with A 225 GLU HG3	Favored (95.56%) General / -61.8,-45.0	Favored (40.4%) *t80* chi angles: 164.4,76.4	0.08Å	-	-	-
A 271		CYS	26.06	-	Favored (24.23%) Pre-Pro / -97.8,132.8	Favored (18.1%) *t* chi angles: 191.8	0.05Å	-	-	-
A 272		PRO	16.46	-	Favored (30.45%) Trans-Pro / -67.8,-10.9	Favored (10.3%) *Cg_endo* chi angles: 15.4,335.5,23.2	0.04Å	-	-	-
A 273		THR	18.28	-	Favored (62.03%) General / -70.2,-13.1	Favored (66.5%) *p* chi angles: 62.9	0.03Å	-	-	-
A 274		ASN	22.14	-	Favored (19.16%) General / -111.6,18.4	Favored (43.1%) *m110* chi angles: 298.3,125.2	0.01Å	-	-	-
A 275		LEU	17.87	-	Allowed (1.79%) General / -73.2,-175.3	Favored (29.6%) *mt* chi angles: 292.4,159.8	0.02Å	-	-	-
A 276		GLY	16.54	-	Favored (5.24%) Glycine / 77.7,-119.3	-	-	-	-	-
A 277		THR	12.84	-	Favored (66.02%) General / -68.7,-27.6	Favored (70.2%) *p* chi angles: 62.2	0.06Å	-	-	-
A 278		THR	15.79	-	Allowed (1.68%) General / 63.4,4.3	Allowed (1.9%) *p* chi angles: 81.9	0.06Å	-	-	-
A 279		ILE	21.48	-	Favored (38.08%) Ile or Val / -89.2,130.8	Allowed (1.1%) *mt* chi angles: 275.4,182.8	0.05Å	-	-	-
A 280		ARG	43.96	-	Favored (12.16%) General / -121.7,107.3	Favored (57.3%) *ttt-90* chi angles: 189.6,182.7,179.8,264	0.04Å	-	-	-
#	Alt	Res	High B	Clash > 0.4Å	Ramachandran	Rotamer	Cβ deviation	Bond lengths	Bond angles	Cis Peptides
			Avg: 29.18	Clashscore: 5	Outliers: 1 of 340	Poor rotamers: 0 of 287	Outliers: 0 of 322	Outliers: 0 of 347	Outliers: 0 of 347	Non-Trans: 1 of 345

A 281	ALA	23.3	-	Favored (48.24%) General / -102.0,129.4	-	0.03Å	-	-	-
A 282	SER	21.91	-	Favored (44.59%) General / -144.0,157.0	Favored (70.6%) *p* chi angles: 59	0.01Å	-	-	-
A 283	VAL	23.44	-	Favored (31.46%) Ile or Val / -126.7,148.3	Favored (27.2%) *m* chi angles: 299.8	0.01Å	-	-	-
A 284	HIS	58.48	-	Favored (26.79%) General / -95.9,115.0	Favored (3.4%) *m170* chi angles: 270.6,202.8	0.01Å	-	-	-
A 285	ILE	25.22	-	Favored (23.34%) Ile or Val / -141.5,146.4	Favored (17.5%) *tt* chi angles: 185.1,171.8	0.05Å	-	-	-
A 286	ALA	29.49	-	Favored (40.24%) General / -109.3,119.7	-	0.01Å	-	-	-
A 287	LEU	36.08	-	Favored (57.78%) Pre-Pro / -124.3,72.7	Allowed (0.6%) *mm* chi angles: 288.1,319	0.03Å	-	-	-
A 288	PRO	40.26	-	Favored (21.07%) Trans-Pro / -63.9,-42.0	Favored (33.3%) *Cg_endo* chi angles: 21.9,326.4,31.2	0.04Å	-	-	-
A 289	LYS	46.42	0.44Å HD3 with A 356 ALA HB3	Favored (85.29%) General / -61.6,-47.5	Favored (9.2%) *mttp* chi angles: 279,157.3,154.8,66.9	0.02Å	-	-	-
A 290	LEU	41.23	-	Favored (65.43%) General / -67.6,-21.6	Favored (39.8%) *mt* chi angles: 298,186.6	0.03Å	-	-	-
A 291	ALA	55.95	-	Favored (61.18%) General / -72.4,-23.9	-	0.01Å	-	-	-
A 292	ALA	66.57	-	Favored (58.77%) General / -88.1,-4.4	-	0.02Å	-	-	-
A 293	ALA	72.39	0.52Å O with A 295 ALA N	Favored (4.11%) General / -104.0,-47.7	-	0.02Å	-	-	-

#	Alt	Res	High B	Clash > 0.4Å	Ramachandran	Rotamer	Cβ deviation	Bond lengths	Bond angles	Cis Peptides
A 294		ALA	66.69	-	OUTLIER (0.02)% General / 56.9,-20.1	-	0.04Å	-	-	-
A 295		ALA	71.56	0.52Å N with A 293 ALA O	Favored (13.02%) General / -88.8,-40.6	-	0.00Å	-	-	-
A 296		ALA	67.89	0.44Å O with A 300 ILE HG12	Favored (61.57%) General / -73.5,-27.9	-	0.03Å	-	-	-
A 297		ALA	59.63	-	Allowed (0.93%) General / -64.4,-64.2	-	0.01Å	-	-	-
A 298		GLU	68.38	-	Favored (63.58%) General / -59.0,-25.4	Allowed (1.3%) *mp0* chi angles: 262.1,72.3,183	0.01Å	-	-	-
A 299		ALA	54.02	-	Favored (70.73%) General / -58.2,-34.7	-	0.01Å	-	-	-
A 300		ILE	61.64	0.44Å HG12 with A 296 ALA O	Favored (18.82%) Ile or Val / -83.2,-45.6	Favored (6.1%) *mm* chi angles: 316.6,307.7	0.05Å	-	-	-
**#**	**Alt**	**Res**	**High B**	**Clash > 0.4Å**	**Ramachandran**	**Rotamer**	**Cβ deviation**	**Bond lengths**	**Bond angles**	**Cis Peptides**
			Avg: 29.18	Clashscore: 5	Outliers: 1 of 340	Poor rotamers: 0 of 287	Outliers: 0 of 322	Outliers: 0 of 347	Outliers: 0 of 347	Non-Trans: 1 of 345
A 301		ALA	42.7	-	Favored (18.63%) General / -52.2,-55.8	-	0.02Å	-	-	-
A 302		ALA	48.39	-	Favored (78.84%) General / -62.1,-35.5	-	0.01Å	-	-	-
A 303		LYS	57.52	0.41Å HB2 with S 260 HOH O	Favored (68.01%) General / -72.4,-34.5	Allowed (0.4%) *tttm* chi angles: 151.7,152.3,202.3,253.3	0.00Å	-	-	-
A 304		PHE	39.93	-	Favored (38.67%) General / -100.9,11.9	Favored (58.5%) *m-80* chi angles: 284.9,278.7	0.04Å	-	-	-
A 305		ASN	32.46	-	Favored (15.57%) General / 61.7,38.0	Favored (68.5%) *m-40* chi angles: 305.1,302.6	0.03Å	-	-	-

A 306	LEU	32.9	0.58Å HD11 with A 345 GLY HA3	Favored (22.39%) General / -98.7,149.9	Favored (72.7%) *mt* chi angles: 302.6,180.9	0.01Å	-	-	-
A 307	GLN	54.84	-	Favored (55.5%) General / -109.5,130.5	Allowed (0.6%) *tt0* chi angles: 146.8,159.8,23.1	0.03Å	-	-	-
A 308	VAL	42.27	-	Favored (34.5%) Ile or Val / -107.9,111.1	Favored (85.6%) *t* chi angles: 173.6	0.03Å	-	-	-
A 309	ALA	46.93	-	-	-	0.02Å	-	-	-
A 320	GLY	59.54	-	-	-	-	-	-	-
A 321	GLY	61.05	-	Allowed (0.42%) Glycine / -141.6,-14.6	-	-	-	-	-
A 322	VAL	53.99	-	Favored (42.35%) Ile or Val / -102.1,114.7	Allowed (0.5%) *p* chi angles: 48.1	0.06Å	-	-	-
A 323	TYR	56.8	-	Favored (20.94%) General / -105.2,153.9	Favored (77.9%) *m-80* chi angles: 300.1,287.4	0.05Å	-	-	-
A 324	ASP	46.72	-	Favored (36.03%) General / -103.0,118.1	Favored (14.9%) *t70* chi angles: 174.8,264.8	0.02Å	-	-	-
A 325	ILE	27.91	-	Favored (75.09%) Ile or Val / -122.3,129.7	Favored (27.4%) *mt* chi angles: 303.4,184.3	0.04Å	-	-	-
A 326	SER	26.67	-	Favored (43.7%) General / -142.9,158.3	Favored (57.1%) *p* chi angles: 57.5	0.02Å	-	-	-
A 327	ASN	26.1	-	Favored (53.83%) General / -69.6,140.9	Favored (15.3%) *p0* chi angles: 62.3,292.6	0.02Å	-	-	-
A 328	LYS	43.34	-	Favored (22.16%) General / -85.3,-33.0	Favored (4.1%) *mtpt* chi angles: 290.2,163.5,94.8,144.4	0.04Å	-	-	-
A 329	ARG	25.65	-	Favored (31.84%) General / -88.2,138.0	Favored (44.3%) *mtp180* chi angles: 301.6,157.7,68.8,200.7	0.02Å	-	-	-

#	Alt	Res	High B	Clash > 0.4Å	Ramachandran	Rotamer	Cβ deviation	Bond lengths	Bond angles	Cis Peptides
A 330		ARG	29.18	-	Favored (4.41%) General / -119.6,-32.1	Favored (3.6%) *mmt-90* chi angles: 316.9,259.3,187.1,283.4	0.04Å	-	-	-
**#**	**Alt**	**Res**	**High B**	**Clash > 0.4Å**	**Ramachandran**	**Rotamer**	**Cβ deviation**	**Bond lengths**	**Bond angles**	**Cis Peptides**
			Avg: 29.18	Clashscore: 5	Outliers: 1 of 340	Poor rotamers: 0 of 287	Outliers: 0 of 322	Outliers: 0 of 347	Outliers: 0 of 347	Non-Trans: 1 of 345
A 331		MET	18.5	-	Favored (23.65%) General / -110.5,154.4	Favored (23.3%) *mtp* chi angles: 312,190.9,64.3	0.02Å	-	-	-
A 332		GLY	18	-	Favored (88.43%) Glycine / 84.3,-1.8	-	-	-	-	-
A 333		LEU	19.44	-	Favored (44.46%) General / -141.3,158.0	Allowed (0.8%) *pt* chi angles: 76.7,183.4	0.02Å	-	-	-
A 334		THR	16.91	-	Favored (14.75%) General / -78.5,171.7	Favored (30.3%) *p* chi angles: 69.3	0.02Å	-	-	-
A 335		GLU	18.8	-	Favored (96.04%) General / -60.3,-43.9	Favored (54.9%) *mp0* chi angles: 289.4,77.8,0.5	0.01Å	-	-	-
A 336		TYR	25.85	-	Favored (76.47%) General / -58.2,-49.6	Favored (92.4%) *t80* chi angles: 176.5,77.1	0.02Å	-	-	-
A 337		GLN	24.55	-	Favored (80.8%) General / -64.0,-35.5	Favored (77.1%) *mt0* chi angles: 301.4,181.1,306	0.01Å	-	-	-
A 338		ALA	19.92	-	Favored (79.71%) General / -62.6,-48.4	-	0.02Å	-	-	-
A 339		VAL	19.1	-	Favored (86.53%) Ile or Val / -67.7,-43.5	Favored (50.4%) *t* chi angles: 169.5	0.08Å	-	-	-
A 340		LYS	31.26	-	Favored (71.75%) General / -61.8,-31.6	Allowed (1.7%) *tptp* chi angles: 184.4,74.5,167.8,21.1	0.02Å	-	-	-
A 341		GLU	29.96	-	Favored (71.52%) General / -68.7,-45.5	Favored (21.9%) *tt0* chi angles: 194.6,171.1,54.9	0.04Å	-	-	-

#	Alt	Res	High B	Clash > 0.4Å	Ramachandran	Rotamer	Cβ deviation	Bond lengths	Bond angles	Cis Peptides
A 342		MET	33.72	-	Favored (64.63%) General / -58.8,-52.7	Favored (7.1%) *tmm* chi angles: 198.5,270.9,277.6	0.02Å	-	-	-
A 343		GLN	26.1	-	Favored (30.55%) General / -49.8,-52.7	Favored (21.9%) *tt0* chi angles: 163.6,178.2,298	0.01Å	-	-	-
A 344		ASP	30.84	-	Favored (99.39%) General / -62.2,-42.6	Favored (87.8%) *m-30* chi angles: 293.2,336	0.03Å	-	-	-
A 345		GLY	20.91	0.58Å HA3 with A 306 LEU HD11	Favored (93.54%) Glycine / -61.6,-46.8	-	-	-	-	-
A 346		ILE	21.13	-	Favored (90.94%) Ile or Val / -64.3,-47.1	Favored (3.5%) *mm* chi angles: 292.9,316.3	0.08Å	-	-	-
A 347		LEU	20.49	-	Favored (54.77%) General / -50.4,-44.4	Favored (26.6%) *mt* chi angles: 287,180.8	0.01Å	-	-	-
A 348		GLU	35.93	-	Favored (79.49%) General / -67.4,-44.2	Favored (2.8%) *tp30* chi angles: 210.3,49.6,45.1	0.03Å	-	-	-
A 349		MET	37.9	-	Favored (98.51%) General / -63.5,-42.1	Favored (68.5%) *mtt* chi angles: 294.2,178.6,186.5	0.02Å	-	-	-
A 350		ILE	22.8	-	Favored (88.02%) Ile or Val / -63.0,-40.2	Favored (25.9%) *mt* chi angles: 285.8,178.2	0.06Å	-	-	-
**#**	**Alt**	**Res**	**High B**	**Clash > 0.4Å**	**Ramachandran**	**Rotamer**	**Cβ deviation**	**Bond lengths**	**Bond angles**	**Cis Peptides**
			Avg: 29.18	Clashscore: 5	Outliers: 1 of 340	Poor rotamers: 0 of 287	Outliers: 0 of 322	Outliers: 0 of 347	Outliers: 0 of 347	Non-Trans: 1 of 345
A 351		LYS	29.63	-	Favored (78.47%) General / -55.9,-46.1	Favored (22.1%) *ttmt* chi angles: 187.6,185.2,307.9,183.8	0.04Å	-	-	-
A 352		MET	58.16	-	Favored (92.82%) General / -65.5,-42.0	Favored (22.5%) *mmt* chi angles: 295.7,307.1,166.4	0.00Å	-	-	-
A 353		GLU	35.05	0.46Å HG2 with A 119 TYR	Favored (88.93%) General /	Favored (43.5%) *tp30* chi angles:	0.03Å	-	-	-

				CE1	-59.1,-41.8	181.3,76.9,187.4				
A 354		GLU	47.38	-	Favored (69.34%) General / -68.6,-30.6	Favored (2.6%) *mm-30* chi angles: 285.8,267.1,316.9	0.02Å	-	-	-
A 355		ALA	49.79	-	Favored (23.74%) General / -90.0,-20.1	-	0.00Å	-	-	-
A 356		ALA	46.57	0.44Å HB3 with A 289 LYS HD3	Favored (32.7%) Pre-Pro / -57.0,151.7	-	0.03Å	-	-	-
A 357		PRO	53.26	-	-	Favored (83.5%) *Cg_exo* chi angles: 334.3,6.4,15.5	0.04Å	-	-	-
B 1		NA	41.35	-	-	-	-	-	-	-
S 1		HOH	8.5	-	-	-	-	-	-	-
S 2		HOH	8.22	-	-	-	-	-	-	-
S 3		HOH	12.77	-	-	-	-	-	-	-
S 4		HOH	11.56	-	-	-	-	-	-	-
S 5		HOH	22.16	-	-	-	-	-	-	-
S 6		HOH	17.16	-	-	-	-	-	-	-
S 7		HOH	13.22	-	-	-	-	-	-	-
S 8		HOH	14.72	-	-	-	-	-	-	-
S 9		HOH	13.6	-	-	-	-	-	-	-
S 10		HOH	11.71	-	-	-	-	-	-	-
S 11		HOH	12.98	-	-	-	-	-	-	-
S 12		HOH	24.37	-	-	-	-	-	-	-
**#**	**Alt**	**Res**	**High B**	**Clash > 0.4Å**	**Ramachandran**	**Rotamer**	**Cβ deviation**	**Bond lengths**	**Bond angles**	**Cis Peptides**
			Avg: 29.18	Clashscore: 5	Outliers: 1 of 340	Poor rotamers: 0 of 287	Outliers: 0 of 322	Outliers: 0 of 347	Outliers: 0 of 347	Non-Trans: 1 of 345
S 13		HOH	19.48	-	-	-	-	-	-	-
S 14		HOH	21.09	-	-	-	-	-	-	-
S 15		HOH	18.89	-	-	-	-	-	-	-
S 16		HOH	21.11	-	-	-	-	-	-	-

S 17		HOH	12.05	-	-	-	-	-	-	-
S 18		HOH	15.91	-	-	-	-	-	-	-
S 19		HOH	11.64	-	-	-	-	-	-	-
S 20		HOH	20.36	-	-	-	-	-	-	-
S 21		HOH	23.35	0.50Å O with A 254 GLU OE2	-	-	-	-	-	-
S 22		HOH	23.13	-	-	-	-	-	-	-
S 23		HOH	16.32	-	-	-	-	-	-	-
S 24		HOH	18.21	-	-	-	-	-	-	-
S 25		HOH	20.62	-	-	-	-	-	-	-
S 26		HOH	12.85	-	-	-	-	-	-	-
S 27		HOH	19.37	-	-	-	-	-	-	-
S 28		HOH	18.74	-	-	-	-	-	-	-
S 29		HOH	13.82	-	-	-	-	-	-	-
S 30		HOH	13.29	-	-	-	-	-	-	-
S 31		HOH	23.6	-	-	-	-	-	-	-
S 32		HOH	18.23	-	-	-	-	-	-	-
**#**	**Alt**	**Res**	**High B**	**Clash > 0.4Å**	**Ramachandran**	**Rotamer**	**Cβ deviation**	**Bond lengths**	**Bond angles**	**Cis Peptides**
			Avg: 29.18	Clashscore: 5	Outliers: 1 of 340	Poor rotamers: 0 of 287	Outliers: 0 of 322	Outliers: 0 of 347	Outliers: 0 of 347	Non-Trans: 1 of 345
S 33		HOH	36.42	-	-	-	-	-	-	-
S 34		HOH	25.88	-	-	-	-	-	-	-
S 35		HOH	23.74	-	-	-	-	-	-	-
S 36		HOH	16.54	-	-	-	-	-	-	-

S 37		HOH	17.71	-	-	-	-	-	-	-
S 38		HOH	34.88	-	-	-	-	-	-	-
S 39		HOH	22.08	-	-	-	-	-	-	-
S 40		HOH	13.71	-	-	-	-	-	-	-
S 41		HOH	19.25	-	-	-	-	-	-	-
S 42		HOH	26.67	-	-	-	-	-	-	-
S 43		HOH	25.1	-	-	-	-	-	-	-
S 44		HOH	22.71	-	-	-	-	-	-	-
S 45		HOH	20.81	-	-	-	-	-	-	-
S 46		HOH	26.3	-	-	-	-	-	-	-
S 47		HOH	31.7	-	-	-	-	-	-	-
S 48		HOH	29.57	-	-	-	-	-	-	-
S 49		HOH	21.21	-	-	-	-	-	-	-
S 50		HOH	17.53	-	-	-	-	-	-	-
S 51		HOH	29.17	-	-	-	-	-	-	-
S 52		HOH	21.55	-	-	-	-	-	-	-
**#**	**Alt**	**Res**	**High B**	**Clash > 0.4Å**	**Ramachandran**	**Rotamer**	**Cβ deviation**	**Bond lengths**	**Bond angles**	**Cis Peptides**
			Avg: 29.18	Clashscore: 5	Outliers: 1 of 340	Poor rotamers: 0 of 287	Outliers: 0 of 322	Outliers: 0 of 347	Outliers: 0 of 347	Non-Trans: 1 of 345
S 53		HOH	30.63	-	-	-	-	-	-	-
S 54		HOH	25.19	-	-	-	-	-	-	-
S 55		HOH	27.07	-	-	-	-	-	-	-
S 56		HOH	32.53	-	-	-	-	-	-	-

S 57		HOH	24.2	-	-	-	-	-	-	-
S 58		HOH	39.84	-	-	-	-	-	-	-
S 59		HOH	18.87	-	-	-	-	-	-	-
S 60		HOH	31.08	-	-	-	-	-	-	-
S 61		HOH	24.09	-	-	-	-	-	-	-
S 62		HOH	20.53	-	-	-	-	-	-	-
S 63		HOH	22.4	-	-	-	-	-	-	-
S 64		HOH	20.86	-	-	-	-	-	-	-
S 65		HOH	30.99	-	-	-	-	-	-	-
S 66		HOH	16.89	-	-	-	-	-	-	-
S 67		HOH	32.59	-	-	-	-	-	-	-
S 68		HOH	19.48	-	-	-	-	-	-	-
S 69		HOH	34.3	-	-	-	-	-	-	-
S 70		HOH	21.36	-	-	-	-	-	-	-
S 71		HOH	22.38	-	-	-	-	-	-	-
S 72		HOH	26.88	-	-	-	-	-	-	-
**#**	**Alt**	**Res**	**High B**	**Clash > 0.4Å**	**Ramachandran**	**Rotamer**	**Cβ deviation**	**Bond lengths**	**Bond angles**	**Cis Peptides**
			Avg: 29.18	Clashscore: 5	Outliers: 1 of 340	Poor rotamers: 0 of 287	Outliers: 0 of 322	Outliers: 0 of 347	Outliers: 0 of 347	Non-Trans: 1 of 345
S 73		HOH	19.81	-	-	-	-	-	-	-
S 74		HOH	26.75	-	-	-	-	-	-	-
S 75		HOH	30.57	-	-	-	-	-	-	-
S 76		HOH	20.86	-	-	-	-	-	-	-

#	Alt	Res	High B	Clash > 0.4Å	Ramachandran	Rotamer	Cβ deviation	Bond lengths	Bond angles	Cis Peptides
S 77		HOH	24.54	-	-	-	-	-	-	-
S 78		HOH	24.53	-	-	-	-	-	-	-
S 79		HOH	24.72	-	-	-	-	-	-	-
S 80		HOH	24.4	-	-	-	-	-	-	-
S 81		HOH	37.9	-	-	-	-	-	-	-
S 82		HOH	20.98	-	-	-	-	-	-	-
S 83		HOH	25.13	-	-	-	-	-	-	-
S 84		HOH	25.11	-	-	-	-	-	-	-
S 85		HOH	27.93	-	-	-	-	-	-	-
S 86		HOH	27.15	-	-	-	-	-	-	-
S 87		HOH	27.74	-	-	-	-	-	-	-
S 88		HOH	23.92	-	-	-	-	-	-	-
S 89		HOH	42.57	-	-	-	-	-	-	-
S 90		HOH	29	-	-	-	-	-	-	-
S 91		HOH	32.31	-	-	-	-	-	-	-
S 92		HOH	34.2	-	-	-	-	-	-	-
**#**	**Alt**	**Res**	**High B**	**Clash > 0.4Å**	**Ramachandran**	**Rotamer**	**Cβ deviation**	**Bond lengths**	**Bond angles**	**Cis Peptides**
			Avg: 29.18	Clashscore: 5	Outliers: 1 of 340	Poor rotamers: 0 of 287	Outliers: 0 of 322	Outliers: 0 of 347	Outliers: 0 of 347	Non-Trans: 1 of 345
S 93		HOH	25.65	-	-	-	-	-	-	-
S 94		HOH	29.25	-	-	-	-	-	-	-
S 95		HOH	22.56	-	-	-	-	-	-	-
S 96		HOH	15.03	-	-	-	-	-	-	-

#	Alt	Res	High B	Clash > 0.4Å	Ramachandran	Rotamer	Cβ deviation	Bond lengths	Bond angles	Cis Peptides
S 97		HOH	33.22	-	-	-	-	-	-	-
S 98		HOH	22.93	-	-	-	-	-	-	-
S 99		HOH	26.86	-	-	-	-	-	-	-
S 100		HOH	34.58	-	-	-	-	-	-	-
S 101		HOH	33.61	-	-	-	-	-	-	-
S 102		HOH	37.87	-	-	-	-	-	-	-
S 103		HOH	43.37	-	-	-	-	-	-	-
S 104		HOH	41.07	-	-	-	-	-	-	-
S 105		HOH	37.87	-	-	-	-	-	-	-
S 106		HOH	33.8	-	-	-	-	-	-	-
S 107		HOH	24.55	-	-	-	-	-	-	-
S 108		HOH	26.6	-	-	-	-	-	-	-
S 109		HOH	23.44	-	-	-	-	-	-	-
S 110		HOH	25.46	-	-	-	-	-	-	-
S 111		HOH	24.3	-	-	-	-	-	-	-
S 112		HOH	26.51	-	-	-	-	-	-	-
**#**	**Alt**	**Res**	**High B**	**Clash > 0.4Å**	**Ramachandran**	**Rotamer**	**Cβ deviation**	**Bond lengths**	**Bond angles**	**Cis Peptides**
			Avg: 29.18	Clashscore: 5	Outliers: 1 of 340	Poor rotamers: 0 of 287	Outliers: 0 of 322	Outliers: 0 of 347	Outliers: 0 of 347	Non-Trans: 1 of 345
S 113		HOH	24.51	-	-	-	-	-	-	-
S 114		HOH	40.77	-	-	-	-	-	-	-
S 115		HOH	28.66	-	-	-	-	-	-	-
S 116		HOH	20.3	-	-	-	-	-	-	-

#	Alt	Res	High B	Clash > 0.4Å	Ramachandran	Rotamer	Cβ deviation	Bond lengths	Bond angles	Cis Peptides
S 117		HOH	29.92	-	-	-	-	-	-	-
S 118		HOH	22.84	-	-	-	-	-	-	-
S 119		HOH	36.15	-	-	-	-	-	-	-
S 120		HOH	39.73	-	-	-	-	-	-	-
S 121		HOH	43.31	-	-	-	-	-	-	-
S 122		HOH	34.83	-	-	-	-	-	-	-
S 123		HOH	28.56	-	-	-	-	-	-	-
S 124		HOH	32.17	-	-	-	-	-	-	-
S 125		HOH	30.3	-	-	-	-	-	-	-
S 126		HOH	35.47	-	-	-	-	-	-	-
S 127		HOH	34.3	-	-	-	-	-	-	-
S 128		HOH	29.61	-	-	-	-	-	-	-
S 129		HOH	28.27	-	-	-	-	-	-	-
S 130		HOH	31.79	-	-	-	-	-	-	-
S 131		HOH	33.54	-	-	-	-	-	-	-
S 132		HOH	32.27	-	-	-	-	-	-	-
			Avg: 29.18	Clashscore: 5	Outliers: 1 of 340	Poor rotamers: 0 of 287	Outliers: 0 of 322	Outliers: 0 of 347	Outliers: 0 of 347	Non-Trans: 1 of 345
S 133		HOH	41.57	-	-	-	-	-	-	-
S 134		HOH	43.23	0.49Å O with A 94 LYS HE2	-	-	-	-	-	-
S 135		HOH	36.92	-	-	-	-	-	-	-
S 136		HOH	26.95	-	-	-	-	-	-	-

#	Alt	Res	High B	Clash > 0.4Å	Ramachandran	Rotamer	Cβ deviation	Bond lengths	Bond angles	Cis Peptides
S 137		HOH	23.64	-	-	-	-	-	-	-
S 138		HOH	35.37	-	-	-	-	-	-	-
S 139		HOH	36.14	-	-	-	-	-	-	-
S 140		HOH	17.91	-	-	-	-	-	-	-
S 141		HOH	25.1	-	-	-	-	-	-	-
S 142		HOH	38.76	-	-	-	-	-	-	-
S 143		HOH	40.88	-	-	-	-	-	-	-
S 144		HOH	27.3	-	-	-	-	-	-	-
S 145		HOH	32.24	-	-	-	-	-	-	-
S 146		HOH	16.08	-	-	-	-	-	-	-
S 147		HOH	13.29	-	-	-	-	-	-	-
S 148		HOH	19.06	-	-	-	-	-	-	-
S 149		HOH	20.1	-	-	-	-	-	-	-
S 150		HOH	19.33	-	-	-	-	-	-	-
S 151		HOH	28.93	-	-	-	-	-	-	-
S 152		HOH	34.79	-	-	-	-	-	-	-
**#**	**Alt**	**Res**	**High B**	**Clash > 0.4Å**	**Ramachandran**	**Rotamer**	**Cβ deviation**	**Bond lengths**	**Bond angles**	**Cis Peptides**
			Avg: 29.18	Clashscore: 5	Outliers: 1 of 340	Poor rotamers: 0 of 287	Outliers: 0 of 322	Outliers: 0 of 347	Outliers: 0 of 347	Non-Trans: 1 of 345
S 153		HOH	28.27	-	-	-	-	-	-	-
S 154		HOH	31.88	-	-	-	-	-	-	-
S 155		HOH	26.11	-	-	-	-	-	-	-
S 156		HOH	28.02	-	-	-	-	-	-	-

S 157		HOH	23.81	-	-	-	-	-	-	-
S 158		HOH	35.21	-	-	-	-	-	-	-
S 159		HOH	29.9	-	-	-	-	-	-	-
S 160		HOH	21.78	-	-	-	-	-	-	-
S 161		HOH	34.62	-	-	-	-	-	-	-
S 162		HOH	31.43	-	-	-	-	-	-	-
S 163		HOH	35.69	-	-	-	-	-	-	-
S 164		HOH	29.56	-	-	-	-	-	-	-
S 165		HOH	34.05	-	-	-	-	-	-	-
S 166		HOH	23.28	0.56Å O with A 124 HIS ND1	-	-	-	-	-	-
S 167		HOH	39.56	-	-	-	-	-	-	-
S 168		HOH	30.32	-	-	-	-	-	-	-
S 169		HOH	29.31	-	-	-	-	-	-	-
S 170		HOH	35.63	-	-	-	-	-	-	-
S 171		HOH	21.31	-	-	-	-	-	-	-
S 172		HOH	34.39	-	-	-	-	-	-	-
**#**	**Alt**	**Res**	**High B**	**Clash > 0.4Å**	**Ramachandran**	**Rotamer**	**Cβ deviation**	**Bond lengths**	**Bond angles**	**Cis Peptides**
			Avg: 29.18	Clashscore: 5	Outliers: 1 of 340	Poor rotamers: 0 of 287	Outliers: 0 of 322	Outliers: 0 of 347	Outliers: 0 of 347	Non-Trans: 1 of 345
S 173		HOH	40.07	-	-	-	-	-	-	-
S 174		HOH	30.24	-	-	-	-	-	-	-
S 175		HOH	32.7	-	-	-	-	-	-	-
S 176		HOH	35.4	-	-	-	-	-	-	-

#	Alt	Res	High B	Clash > 0.4Å	Ramachandran	Rotamer	Cβ deviation	Bond lengths	Bond angles	Cis Peptides
S 177		HOH	34.96	-	-	-	-	-	-	-
S 178		HOH	40.25	-	-	-	-	-	-	-
S 179		HOH	32.44	-	-	-	-	-	-	-
S 180		HOH	31.92	-	-	-	-	-	-	-
S 181		HOH	38.23	-	-	-	-	-	-	-
S 182		HOH	32.41	-	-	-	-	-	-	-
S 183		HOH	29.56	-	-	-	-	-	-	-
S 184		HOH	32.4	-	-	-	-	-	-	-
S 185		HOH	44.17	-	-	-	-	-	-	-
S 186		HOH	39.6	-	-	-	-	-	-	-
S 187		HOH	35.14	-	-	-	-	-	-	-
S 188		HOH	48.83	-	-	-	-	-	-	-
S 189		HOH	37.33	-	-	-	-	-	-	-
S 190		HOH	28.2	-	-	-	-	-	-	-
S 191		HOH	30.39	0.78Å O with A 183 ASP OD2	-	-	-	-	-	-
S 192		HOH	36.28	-	-	-	-	-	-	-
#	Alt	Res	High B	Clash > 0.4Å	Ramachandran	Rotamer	Cβ deviation	Bond lengths	Bond angles	Cis Peptides
			Avg: 29.18	Clashscore: 5	Outliers: 1 of 340	Poor rotamers: 0 of 287	Outliers: 0 of 322	Outliers: 0 of 347	Outliers: 0 of 347	Non-Trans: 1 of 345
S 193		HOH	29.36	-	-	-	-	-	-	-
S 194		HOH	43.25	-	-	-	-	-	-	-
S 195		HOH	36.14	-	-	-	-	-	-	-
S 196		HOH	26.42	-	-	-	-	-	-	-

#	Alt	Res	High B	Clash > 0.4Å	Ramachandran	Rotamer	Cβ deviation	Bond lengths	Bond angles	Cis Peptides
S 197		HOH	35.9	-	-	-	-	-	-	-
S 198		HOH	27.85	-	-	-	-	-	-	-
S 199		HOH	27.44	-	-	-	-	-	-	-
S 200		HOH	35.37	-	-	-	-	-	-	-
S 201		HOH	26.18	-	-	-	-	-	-	-
S 202		HOH	26.31	-	-	-	-	-	-	-
S 203		HOH	44.17	-	-	-	-	-	-	-
S 204		HOH	47.73	-	-	-	-	-	-	-
S 205		HOH	44.75	-	-	-	-	-	-	-
S 206		HOH	48.32	-	-	-	-	-	-	-
S 207		HOH	24.08	-	-	-	-	-	-	-
S 208		HOH	40.9	-	-	-	-	-	-	-
S 209		HOH	17.74	-	-	-	-	-	-	-
S 210		HOH	30.58	-	-	-	-	-	-	-
S 211		HOH	35.73	-	-	-	-	-	-	-
S 212		HOH	31.4	-	-	-	-	-	-	-
**#**	**Alt**	**Res**	**High B**	**Clash > 0.4Å**	**Ramachandran**	**Rotamer**	**Cβ deviation**	**Bond lengths**	**Bond angles**	**Cis Peptides**
			Avg: 29.18	Clashscore: 5	Outliers: 1 of 340	Poor rotamers: 0 of 287	Outliers: 0 of 322	Outliers: 0 of 347	Outliers: 0 of 347	Non-Trans: 1 of 345
S 213		HOH	25.82	-	-	-	-	-	-	-
S 214		HOH	38.71	-	-	-	-	-	-	-
S 215		HOH	42.61	-	-	-	-	-	-	-
S 216		HOH	48.63	-	-	-	-	-	-	-

S 217		HOH	42.02	-	-	-	-	-	-	-
S 218		HOH	33.13	-	-	-	-	-	-	-
S 219		HOH	20.71	-	-	-	-	-	-	-
S 220		HOH	26.11	-	-	-	-	-	-	-
S 221		HOH	32.32	-	-	-	-	-	-	-
S 222		HOH	34.5	0.65Å O with A 160 GLU O	-	-	-	-	-	-
S 223		HOH	39.5	-	-	-	-	-	-	-
S 224		HOH	44.67	-	-	-	-	-	-	-
S 225		HOH	38.45	-	-	-	-	-	-	-
S 226		HOH	40.91	-	-	-	-	-	-	-
S 227		HOH	40.59	-	-	-	-	-	-	-
S 228		HOH	45.19	-	-	-	-	-	-	-
S 229		HOH	32.02	-	-	-	-	-	-	-
S 230		HOH	23.79	-	-	-	-	-	-	-
S 231		HOH	35.68	0.76Å O with A 229 ARG NH1	-	-	-	-	-	-
S 232		HOH	36.51	-	-	-	-	-	-	-
**#**	**Alt**	**Res**	**High B**	**Clash > 0.4Å**	**Ramachandran**	**Rotamer**	**Cβ deviation**	**Bond lengths**	**Bond angles**	**Cis Peptides**
			Avg: 29.18	Clashscore: 5	Outliers: 1 of 340	Poor rotamers: 0 of 287	Outliers: 0 of 322	Outliers: 0 of 347	Outliers: 0 of 347	Non-Trans: 1 of 345
S 233		HOH	40.59	-	-	-	-	-	-	-
S 234		HOH	38.23	-	-	-	-	-	-	-
S 235		HOH	44.76	-	-	-	-	-	-	-

#	Alt	Res	High B	Clash > 0.4Å	Ramachandran	Rotamer	Cβ deviation	Bond lengths	Bond angles	Cis Peptides
S 236		HOH	33.8	-	-	-	-	-	-	-
S 237		HOH	48.35	-	-	-	-	-	-	-
S 238		HOH	42.89	-	-	-	-	-	-	-
S 239		HOH	44.32	-	-	-	-	-	-	-
S 240		HOH	26.71	-	-	-	-	-	-	-
S 241		HOH	34.87	0.85Å O with A 11 GLU OE1	-	-	-	-	-	-
S 242		HOH	28.41	-	-	-	-	-	-	-
S 243		HOH	35.96	0.57Å O with A 97 ASP OD1	-	-	-	-	-	-
S 244		HOH	33.34	-	-	-	-	-	-	-
S 245		HOH	50.32	-	-	-	-	-	-	-
S 246		HOH	31.1	-	-	-	-	-	-	-
S 247		HOH	36.32	-	-	-	-	-	-	-
S 248		HOH	20.6	-	-	-	-	-	-	-
S 249		HOH	26.22	-	-	-	-	-	-	-
S 250		HOH	32.22	-	-	-	-	-	-	-
S 251		HOH	30.16	-	-	-	-	-	-	-
S 252		HOH	36.21	-	-	-	-	-	-	-
**#**	**Alt**	**Res**	**High B**	**Clash > 0.4Å**	**Ramachandran**	**Rotamer**	**Cβ deviation**	**Bond lengths**	**Bond angles**	**Cis Peptides**
			Avg: 29.18	Clashscore: 5	Outliers: 1 of 340	Poor rotamers: 0 of 287	Outliers: 0 of 322	Outliers: 0 of 347	Outliers: 0 of 347	Non-Trans: 1 of 345
S 253		HOH	41.52	-	-	-	-	-	-	-
S 254		HOH	33.49	-	-	-	-	-	-	-

S 255	HOH	45.6	-	-	-	-	-	-	-
S 256	HOH	49.77	-	-	-	-	-	-	-
S 257	HOH	26.33	-	-	-	-	-	-	-
S 258	HOH	45.13	-	-	-	-	-	-	-
S 259	HOH	45.7	0.43Å O with A 29 LYS NZ	-	-	-	-	-	-
S 260	HOH	44.19	0.41Å O with A 303 LYS HB2	-	-	-	-	-	-
S 261	HOH	47.53	-	-	-	-	-	-	-
S 262	HOH	35.38	-	-	-	-	-	-	-
S 263	HOH	45.76	-	-	-	-	-	-	-
S 264	HOH	39.55	-	-	-	-	-	-	-
S 265	HOH	42.7	-	-	-	-	-	-	-
S 266	HOH	35.81	-	-	-	-	-	-	-

About MolProbity | Website for the Richardson Lab | Using ecloud x-H | Internal reference 4.4
