# Supplementary material for: Biochemical and structural characterization of a novel arginine kinase from the spider Polybetes pythagoricus
Source: PeerJ. 2017 Sep 11;5:e3787. doi: 10.7717/peerj.3787 (PMC5598448; doi:10.7717/peerj.3787)
Supplement: Supplemental Information 6 [file peerj-05-3787-s006.docx]

***Marine Genomics***

*Regular Research Paper*

***De novo* assembly and transcriptome characterization of the freshwater prawn *Palaemonetes argentinus*: implications for a detoxification response**

**Supplementary materials**

**S1. Description of the transcriptome assembly**

***P. argentinus de novo* transcriptome assembly**

A total of 50,625,940 Illumina paired-end reads from total shrimp RNA yielded a total of ~7.29 Gb. The quality for all read pairs was above Q30 (which is equivalent to the probability of an incorrect base call in 1000 times, and a base call accuracy of 99.9%) and no further quality filtering was required before the transcriptome assembly. Sequence data was *de novo* assembled using the Trinity pipeline (Grabherr et al., 2011).

Despite RNA-seq reads provided quality scores, only 50.9% of the transcripts had one or more translations giving a total of 15,476 predicted proteins or open reading frames (ORF) with known biological function, and the remaining reads resulted from non-coding RNAs, including rRNA which, regardless of the mRNA enrichment process in the library construction, there are always presence of it. Of these protein-coding transcripts, 66.15% found a match in at least one of the functional annotation approaches. Regarding the transcript count, those with some annotation information represented 30.24% of the total number of transcripts.

**Functional characterization of *P. argentinus* transcriptome.**

To develop molecular markers using functional candidate genes, predicted proteins with KEGG and KOG annotation were grouped based on their frequency, and the top 20 functional annotations are shown in supplementary figures S1A and S1B, respectively.

In the case of KEGG functions, three groups contained ~25% of the annotated proteins corresponding to K01183-Chitinase, K03102-Squid and K00799-Glutathione S-transferase activities. As for the KOG annotation, also ~25% of the functions were represented by KOG3627-Trypsin, KOG2462-C2H2-type Zn-finger protein, and KOG3513-Neural cell adhesion molecule L1. Finally, counts from PFAM domain frequencies (Figure S2) revealed functions with high rates such as Zinc-finger double domain (PF13465), RNA recognition motif (PF00076) and reverse transcriptase domain (PF00078) with more than 80% of frequency.

Comparing the PFAM annotation for domain functions of *P. argentinus* (2,160 different PFAM domains) and *L. vannamei* annotations (3,154) (Ghaffari et al., 2014) an overlap of 1,623 domain functions was found, which represents ~75% of the total annotated domains for *P. argentinus*. From the remaining 537 functional domain annotations, 14 domains belonged to ribosomal proteins.

A list of the 20 most expressed transcripts either with or without product annotation is shown in Supp. Table S1. Most of the highly expressed transcripts have translation product annotation related to muscle proteins, which can be explained by the predominant amount of muscle tissue in the shrimp body. In the case of un-annotated transcripts, c10387_g1_i1 (GEFN01000632.1) and c8449_g1_i1 (GEFN01022287.1), they presented a transmembrane prediction and signal peptide region, respectively.

**Mitochondrial transcripts**

To detect transcripts belonging to the *P. argentinus* mitochondrion, the translation of all the reconstructed transcripts was repeated, although using the arthropod mitochondrial genetic code. After comparing against mitochondrial genes encoding proteins from the dragonfly *P. serenus*, it was observed that most of the mitochondrial genes were expressed as shown in Supp. Table S2.

**Enzyme Classification (EC) and KEGG pathway analysis of the transcriptome**

The potentially functional enzymes on the *P. argentinus* transcriptome were characterized based on the predictions of Enzyme Commission (EC) numbers for each sequence using Blast2GO software. In total 1,881 sequences were related to EC numbers, and the enzyme classification revealed hydrolases as the largest group of *P. argentinus* enzymes (47.5%, 894 transcripts), followed by transferases (25.1%, 472 transcripts), oxidoreductases (15.3%, 287 transcripts), ligases (5.3%, 99 transcripts), isomerases (4.1%, 77 transcripts) and lyases (2.8%, 52 transcripts) (Supp. Fig. S3).

The number of sequences for each enzyme family is reported in supporting material (Supplementary Figures S4-S9, S11). The 15,476 proteins from shrimp transcriptome were further characterized by KEGG pathway analysis. The predicted enzymes were distributed in 334 KEGG pathways (Supplementary Table S1).

**S.2. Potential biomarkers in *P. argentinus*.**

Several proteins realted to with drug metabolism (12 with the drug metabolism-other enzymes, 7 with the metabolism of xenobiotics by cytochrome P450 and 5 with the drug metabolism-cytochrome P450) were found in the transcriptome, indicating a role in xenobiotic detoxification.

***Phase I Detoxification-* Cytochrome P450 monooxygenases**

Cytochrome P450 monooxygenases convert a broad range of lipophilic compounds into more hydrophilic derivatives. They play a critical role both in the synthesis and processing of endogenous metabolites plus degradation of xenobiotic compounds, being a key component of phase I detoxification systems (Feyereisen, 2006).

Four *P. argentinus* CYP sequences, related to a mitochondrial clan, showed high identity percentage to CYP302A1, CYP362A1 and CYP362A2 of *Daphnia pulex*. CYP302A1, a highly conserved Halloween gene, is involved, together with CYP314A1 and CYP315A1 in ecdysone synthesis. Insects share these three genes to synthesize the molting hormone 20-hydroxyecdysone, although they lack three mitochondrial CYP genes found in the water flea *D. pulex*: CYP362A1, CYP362A2 and CYP363A1. These suggest that these CYPs have diverged within arthropods and probably have new or novel roles in other invertebrates (Baldwin et al., 2009).

One of the 13 CYP2-clan sequences found in this transcriptome is similar to the CYP330A1 gene described in the shore crab *Carcinus maenas.* Expression of this gene was induced by ecdysone, ponasterone A, benzo(a)pyrene and phenobarbital in the hepatopancreas of male intermoult crabs (Rewitz et al., 2003), suggesting different roles both in ecdysteroid catabolism and in detoxification of environmental pollutants. Six sequences of *P. argentinus* matched with members of the CYP2L subfamily of the spiny lobster *Panulirus argus.* The last two CYP2-clan sequences found in the *P. argentinus* transcriptome showed homology with subfamily CYP379B of both lobster and crabs, and may be related to a physiological response to environmental stressors (Baldwin et al., 2009).

The CYP3 clan consists of several detoxification enzymes of both xenobiotic and endobiotic compounds. Three sequences belonging to clan 3 were identified in this study, all showed high identity to *D. pulex* CYP360A4, CYP360A5 and CYP361A1, respectively.

The CYP4 family is the most represented in aquatic invertebrates (Snyder, 2007). The seven sequences belonging to the CYP4 family found in *P. argentinus* show high sequence identity with CYP4V16 of the shore crab *C. maenas*. Genes belonging to the CYP3 and CYP4 families were proposed to have important roles in detoxification and are frequently linked in insects with the potential to acquire resistance to chemical insecticides (David et al., 2003).

***Phase II detoxification- Glutathione* S*-transferase***

Alignments of cds.comp13424 (GEFN01006054.1) and cds.comp23331 (GEFN01016146.1) from *P. argentinus* revealed similitude with the conserved microsomal GST class of the insects *Laodelphax striatela* (AEY80035) and *Papilio xuthus* (BAM18639.1). Microsomal GSTs are membrane-associated proteins that are involved in both eicosanoid and GSH metabolisms (Bresell et al., 2005). The transcript sequences found in *P. argentinus* shared the motif VERVRRXHLNDXENIX, which is described in microsomal GSTs from almost all crustaceans (Supp. Figure S10).

Although in the case of *P. argentinus*, the substitution of a glutamine residue for leucine was observed. This substitution was also found in the crustacean *C. finmarchicus* and was considered species-specific (Roncalli et al., 2015).

***Enzymatic antioxidant defense***

An inevitable consequence of aerobic metabolism is the production of oxygen reactive species (ROS) that induce oxidative damage including enzyme inactivation, protein degradation, DNA damage and lipid peroxidation. To prevent cellular damage, enzymatic antioxidant defenses are deployed to scavenge oxygen reactive compounds including superoxide dismutase (SOD, EC 1.15.1.1 ), catalase (CAT, EC 1.11.1.6) and glutathione peroxidase (GPx, EC 1.11.1.9) (Livingstone, 2003).

SODs are essential enzymes that occur in virtually all oxygen-respiring organisms, constituting the first line of defense against oxidative damage by catalyzing the dismutation of O_2_^-^ to H_2_O_2._ (Fridovich, 1995).

Regarding MnSOD, in decapod crustaceans there are two types: mitochondrial (mMnSOD) and cytosolic (cMnSOD) isoforms. mMnSODs are found in plants, bacteria, vertebrates, and invertebrates, while cMnSOD is found only in crustaceans (Li et al., 2010). Both isoforms have been identified in crustaceans, including *Callinectes sapidus* (AF264029, AF264030), *Macrobrachium rosenbergii* (DQ073104, DQ157765), *Marsupenaeus japonicas* (GQ181123, GQ478988), *Fenneropenaeus chinensis* (EF427949, DQ205424), *Procambarus clarki* (EU254488, FJ892724), *Cherax quadricarinatus* (JQ040506, JQ763321), and *Penaeus monodon* (AY726542, KC461130). *P. argentinus* showed a high percent identity with two *E. sinensis* MnSODs: one corresponding to cMnSOD (ACV41936.1) (97% identity) and the other to mMnSOD isoform (AGV76057.1) (85% identity). Both sequences in *P. argentinus* have the putative MnSOD signature motif (DVWEHAYY) the same as in the decapod *Exopalaemon carinicauda* {Ren, 2015 #75}. In addition, mMnSOD in *P. argentinus* have four putative manganese-binding sites (H48, H96, D180, and H184), which are consistent with other mMnSOD sequences from marine decapods (Cheng et al., 2006). The origin of cMnSOD may be a duplication event, as old as the origin of the arthropod phylum) resulting a protein with a new subcellular location (Brouwer et al., 2003).

Four sequences encoding for glutathione peroxidases (GPx) were identified in the *P. argentinus* transcriptome, with sequences ranging from approximately 68 to 138 amino acids. After a BLASTp search, the best hit corresponded to GPx from horseshoe crabs, crabs, lobster or prawns. The sequence of *P. argentinus* cds.comp10039 (GEFN01000482.1) showed a high percent identity with GPx-like of *Limulus polyphemus* (XP013772703.1), cds.comp10174 (GEFN01000201.1) with phospholipid GPx of *Macrophthalmus japonicus* (AHJ86274.1), cds.comp9895 (GEFN01024176.1) with GPx of *Palaemon carinicauda* (AGJ03551.1) and cds.comp6948 (GEFN01022279.1) with glutathione GPx of *Procambarus clarkii*. Each of the four sequences has a conserved active site motif “^151^WNFEKF^156^”. Also in cds.comp10039 it was substituted in the active site, F153 by W; and in cds.comp10174 (GEFN01000201.1), E154 was substituted by T.

It is important to highlight that in cds.comp9895 the presence of the Se-GPx signature motif ^63^LAFPCNQF^70^ was observed which shared identity with the selenium-dependent subclass GPx (Se-GPx). This isoform is responsible for the reduction of organic and inorganic peroxides, such as hydroperoxide (ROOH) and hydrogen peroxide (H_2_O_2_). Moreover, the amino acids Q73 and W141 were found that are involved in the fixation of selenium and the putative N-glycosylation site ^75^NNT^77^ (Duan et al., 2013).

As mentioned above, catalase (CAT) is an important antioxidant enzyme that exists in virtually all oxygen-respiring organisms. The main function of CAT is catalyzing the conversion of H_2_O_2_ to water and molecular oxygen, but also uses H_2_O_2_ to oxidize toxins such as phenols, formic acid, formaldehyde and alcohols (Chelikani et al., 2004). Two CAT coding sequences were identified in the *P. argentinus* transcriptome, with translated sequences for approximately 101 (cds.comp20365, GEFN01012618.1) and 240 (cds.comp15814, GEFN01004874.1) amino acids. After a BLAST search against non-redundant protein sequences (nr) using Blastp, the best hit corresponded to different regions of CAT from the prawn *Palaemon carinicauda* catalase-clade-3 (KF367466.1) with high percent identities (96 and 93%, respectively).

It should be noted that GPx plays a significant role in H_2_O_2_ detoxification in vertebrates, while CAT fulfills this function in invertebrates (Livingstone et al., 1992).

**Osmoregulation protein comparison with a marine shrimp**

*P. argentinus* is a widely distributed crustacean species in South America in fresh water and lagoons with variable salinity levels. A key factor for growth, distribution, and survival of crustaceans inhabiting the marine environment is salinity (Anger, 2003).

A study has recently described the adaptation mechanisms and pathways related to salinity stress in *Litopenaeus vannamei* using transcriptomics, and a significantly differential expression of some genes involved in osmotic regulation was observed (Hu et al., 2015). A G-protein (guanine nucleotide-binding protein) activates the K^+^, Cl^−^, and Na^+^ channels through a signaling pathway, while the α-subunit can regulate ion channels indirectly (through second messengers) or directly by physically interacting with the channel protein (Dascal, 2001). In this study, a 72-residue fragment of the guanine nucleotide-binding protein G(q) alpha subunit of *P. argentinus* was found in the transcriptome (cds.comp19945_c0_seq1, GEFN01005018.1). This sequence showed high identity with those reported in other crustaceans such as *Marsupenaeus japonicus* (43%, AB488394.1), *Limulus polyphemus* (41%, NM_001314113.1), *Daphnia pulex* (39%, EFX80373.1) and *L. vannamei* (43%, AY626792.1).

The STE20-like kinases belong to the MAP kinase cascade and participate in chloride transport regulation and cell volume regulation, via phosphorylation of channels (Strange et al., 2006). An 88-amino acid fragment (cds.comp1882_c0_seq1, GEFN01017124.1) was found in the transcriptome of *P. argentinus*. This fragment shared a 76% pairwise identity to the STE20-like kinase of *L. vannamei* (KP322128.1), and the closest sequence available in GenBank corresponds to that reported for the coelacanth fish (*Latimeria chalumnae* XP_005994681) with a 44% pairwise identity at the amino acid level.

The crustacean gills are multifunctional organs that serve, among other functions, as the primary site of osmoregulatory ion transport. A nearly universal assumption is that the Na/K pump, also called Na^+^/K^+^ ATPase, functions as the major driving force for ion transport across gills in decapods and other crustaceans (Towle and Weihrauch, 2001). In this study, an 863 amino acid fragment, identified as a Na^+^/K^+^ ATPase, was found within the transcripts of the grass shrimp *P. argentinus* (cds.comp4027_c0_seq1, GEFN01018493.1). This sequence shared an 85% identity with the a1-subunit of the Na+/K+ ATPase of the crustacean *Artemia parthenogenetica* (AJ269679.2).

In addition, two other sequences putatively encoding for a vacuolar-type H+-ATPase (V-ATPase) (cds.comp4196_c0_seq1, GEFN01018798.1) and a sarco/endoplasmic reticulum calcium-ATPase (SR Ca2+ ATPase) (cds.comp5637_c0_seq1, GEFN01019318.1) were identified from the transcriptome of the grass shrimp *P. argentinus*. The V-ATPase of *P. argentinus* showed an 88.79% identity with that of the white shrimp *Litopenaeus vannamei* (ACM16806.1), while the SR Ca^2+^ATPase found in this study shared an 80% identity with that reported on *Daphnia pulex* (EFX65740.1). Previous work has demonstrated that a V-ATPase participates together with the Na^+^/K^+^-ATPase in energizing osmoregulatory ion uptake in the freshwater crayfish (Weihrauch et al., 2001). This information is consistent with the notion that *P. argentinus*, which inhabits low-salinity conditions, possesses a gene encoding for a V-ATPase. A recent report presents a sarco/endoplasmic reticulum Ca^2+^ATPase in the white shrimp *P. vannamei*, and it highlights the essential role played by this enzyme in osmotic adjustment (Wang et al., 2013).

**DNA replication**

DNA replication is an essential biochemical process that must be conducted with high fidelity. Primary components of this process are the DNA polymerases and the current RNA-seq analysis identified several candidate genes encoding enzymes responsible for DNA replication. Transcripts encoding for the DNA polymerase delta (δDNApol) catalytic subunit ranging from 86 (cds.comp23154_c0_seq1, GEFN01003375.1) to 245 amino acids (cds.comp12866_c0_seq1, GEFN01002652.1). This domain is essential for nuclear DNA replication and the elongation and maturation of the Okazaki fragments in the lagging strand. These fragments were most similar to the δDNApol subunits of freshwater crayfish *Procambarus clarkii* (87%) (BAO20827.1) and the mantis shrimp *Oratosquilla oratoria* (87%) (BAO20826.1).

In addition, a transcript encoding for a DNA polymerase epsilon (εDNApol) (cds.comp14989_c0_seq1, GEFN01008396.1) was identified. This transcript, involved in the replication of the leading strand, had a 110 amino acid length. It was most similar to that of the jumping ant *Harpegnathos saltator* (51%) (EFN77324.1) and the fruit fly *Drosophila melanogaster* (64%) (NP_611669.1) (Sanchez Garcia et al., 2009).

Even one of the four subunits of the alpha polymerase-primase complex were identified in the KEGG pathway. This complex is involved in the initiation of the DNA replication because all polymerases lack the capacity to start DNA replication from a single strand, all requires the 3´-end of a primer and here is where the alpha-primase complex is involved (Garg and Burgers, 2005). In the *P. argentinus* transcriptome the α-2 sub unit was identified with a 210 amino acids length (cds.comp14347_c0_seq1, GEFN01010600.1), and had identity with the Atlantic horseshoe crab *Limulus Polyphemus* (52%) (XP_013771709.1), with the Asian swallow tail *Papilio xuthus* (47%) (KPI92391.1), and with the owl limpet *Lottia gigantea* (47%) (XP_009046367.1).

**Bioenergetics**

ATP is considered as the “universal energy currency” for most biological processes (Baldwin et al., 2009). Since central metabolic pathways and bioenergetics are essential in conferring to all living organisms, those were analyzed in the *P. argentinus* transcriptome. According to the cellular components classification (Supplementary Figure 8), both membrane bound and non-membrane bound organelle components are highly represented in the transcriptome of this species.

Mitochondrial functions are essential to cellular homeostasis, besides ATP synthesis, for the antioxidant response to oxidative stress, apoptotic mechanisms among others (Tuena de Gomez-Puyou et al., 1999).

The *P. argentinus* transcriptome include partial and full sequences encoding nine enzymes from glycolysis and gluconeogenesis pathways from the α-D-glucose-1-PO_4_ conversion to D-glucose-6–PO_4_ by phosphoglucomutase (E.C. 5.4.2.2) to all those proteins that participate in the conversion of glucose into pyruvate (Müller et al., 2012).

As well, the existence of a transcript encoding an L-lactate dehydrogenase (comp6159_c0_seq1, GEFN01000673.1) confirms the ability of this species to undergo anaerobic metabolism, probably during oxidative stress conditions that are common in the aquatic environment due to periods of low oxygen concentration. This full-length protein shares length and high identity to other crustacean proteins such as that of the white leg shrimp *Litopenaeus vannamei* (90%, AEC12819), and the water flea *Daphnia pulicaria* (69%, AGR85217), and mosquitoes species such as *Aedes aegypti* (69%, XP001662150) and *Culex quinquefasciatus* (69%; XP001864915).

All of the Krebs cycle enzymes were found as annotated transcripts in the current *P. argentinus* transcriptomic database including the proteins that catalyze each cycle step from pyruvate conversion into acetyl-CoA to the malate dehydrogenase that reversibly catalyzes the oxidation of malate to oxaloacetate. An especially interesting protein is the succinate dehydrogenase or complex II, which is located in the inner mitochondrial membrane and functions in the Krebs cycle and the respiratory chain in mitochondria (Cimen et al., 2010). The complex is comprised in *P. argentinus* by four subunits including the succinate dehydrogenase subunit (cds.comp10307_c0_seq1, GEFN01000551.1).

Cells can accumulate large quantities of phosphagens, which are widely spread in nature. In invertebrates, high-energy phosphorylated compounds, such as phosphoarginine, phospholombricine, phosphoglycocyamine and phosphotaurocyamine (Ellington, 2001).

Despite substrate specificity and source, phosphagen kinases shares well-conserved amino acid sequence (~40% identity) in invertebrates. Most described arginine kinases (AKs) are monomeric (~40 kDa) (Garcia-Orozco et al., 2007; Zhou et al., 1998). However, in the sea cucumber (*Stichopus japonicus*) and in sea anemona (*Anthopleura japonicus)* a homodimeric protein of approximately 80 kDa was found (Suzuki et al., 1999). The current *P. argentinus* transcriptome analysis shows that AK is one of most highly expressed transcripts related to muscle proteins (see Table 2, section A). Sequence alignments show four expressed transcripts in *P. argentinus* transcriptome annotated as AK protein (cds.comp6637, GB GEFN01018817.1; cds.comp8961, GB GEFN01023367.1; cds.comp9908, GB GEFN01020995.1; cds.comp22727, GB GEFN01015443.1).

Detailed analysis shows two partial transcripts (cds.comp22727, 51 amino acids; and cds.comp6637, 260 amino acids) having 89% and 87 % identity to monomeric AK from *Homarus gammarus* (CAA48654.1) and *L. vannamei* ABI98020), respectively.

Surprisingly, transcript cds.comp9908 is more similar to dimeric AKs from sea cucumber *Apostichopus japonicus* (Q9XY07) and sea anemone *Anthopleura japónica* (BAA22888.1) with 44 % and 46% identity, respectively. Also shows similar values toward octameric-mitochondrial creatine kinase type-U from chicken (45 %, X96403.1) and human (46%, J04469). *P. argentinus* cds.comp9908 transcript show similar identity between both dimeric AKs and creatine kinase, which is consistent with previous reports of multimeric AKs. Overall *P. argentinus* annotated AK transcript is more similar to creatine kinase than monomeric AKs. To the best of our knowledge, this is the first result supporting the presence of monomeric and dimeric AKs in the same crustacean. These findings open the possibility to study phylogenetic and evolutionary aspects related to substrate specificity in guanidine kinases family.

**Digestion**

Crustaceans produce highly active digestive enzymes that hydrolyze major food items (Fernández-Gimenez, 2013). These are comprised by proteases, lipases and glucanases. In the present work, several trypsin-like sequences ranging from 85 to 266 amino acids in length were identified. However, only two canonical trypsin sequences (cds.comp.5870_c0_seq1; GEFN01018754.1; cds.comp.5870_c1_seq1, GEFN01018755.1) were found. Those were not assembled as a single transcript, so distinguishing whether they are isoforms or if *P. argentinus* has two trypsin genes is a difficult task.

After a BLASTP search against the non-redundant protein database, high identity hits with *Panulirus argus* trypsin isoform 1a (ADB66711.1), *Litopenaeus vannamei* trypsin (CAA60129.1) and *Daphnia pulex* trypsin (EFX80960.1) were found, and catalytic and active site pocket residues as well.

For carbohydrate digestion, we found two transcripts: cds.comp.10280_c0_se11 (GEFN01000718.1) and cds.comp.10280_c1_seqc1 (GEFN01000716.1) which are similar to *Macrobrachium rosenbergii*, *L. vannamei* and *D. pulex* amylases. The sequence of *P. argentinus* cds.comp10280_c1 has the catalytic site active of amylase and the sequence cds.comp10280_c0 contain the putative Ca^2+^ binding site. The adaptive significance of α-amylase polymorphism in the shrimp is not understood yet, and constitutes at challenge since it is important for aquaculture to know if diets with more carbohydrates than the natural diet can be properly utilized for energy production (Van Wormhoudt and Sellos, 2003).

**REFERENCES**

Anger, K., 2003. Salinity as a key parameter in the larval biology of decapod crustaceans. Invertebr. Reprod. Dev. 43, 29-45.

Baldwin, W.S., Marko, P.B., Nelson, D.R., 2009. The cytochrome P450 (CYP) gene superfamily in *Daphnia pulex*. BMC Genomics. 10, 169.

Bresell, A., Weinander, R., Lundqvist, G., Raza, H., Shimoji, M., Sun, T.H., Balk, L., Wiklund, R., Eriksson, J., Jansson, C., Persson, B., Jakobsson, P.J., Morgenstern, R., 2005. Bioinformatic and enzymatic characterization of the MAPEG superfamily. FEBS J. 1 272, 1688-1703.

Brouwer, M., Hoexum-Brouwer, T., Grater, W., Brown-Peterson, N., 2006. Replacement of a cytosolic copper/zinc superoxide dismutase by a novel cytosolic manganese superoxide dismutase in crustaceans that use copper (haemocyanin) for oxygen transport. Biochem J. 374, 219-228.

Chelikani, P., Fita, I., Loewen, P.C., 2004. Diversity of structures and properties among catalases. Cell Mol. Life Sci. 61, 192-208.

Cheng, W., Tung, Y.H., Chiou, T.T., Chen, J.C., 2006. Cloning and characterisation of mitochondrial manganese superoxide dismutase (mtMnSOD) from the giant freshwater prawn *Macrobrachium rosenbergii*. Fish Shellfish Immunol. 21, 453-466.

Cimen, H., Han, M.J., Yang, Y., Tong, Q., Koc, H., Koc, E.C., 2010. Regulation of succinate dehydrogenase activity by SIRT3 in mammalian mitochondria. Biochemistry. 49, 304-311.

Dascal, N., 2001. Ion-channel regulation by G proteins. Trends Endocrinol. Metab. 12, 391-398.

David, P., Dauphin-Villemant, C., Mesneau, A., Meyran, J.C., 2003. Molecular approach to aquatic environmental bioreporting: differential response to environmental inducers of cytochrome P450 monooxygenase genes in the detritivorous subalpine planktonic Crustacea, *Daphnia pulex*. Mol. Ecol. 12, 2473-2481.

Duan, Y., Liu, P., Li, J., Li, J., Chen, P., 2013. Expression profiles of selenium dependent glutathione peroxidase and glutathione S-transferase from *Exopalaemon carinicauda* in response to *Vibrio anguillarum* and WSSV challenge. Fish Shellfish Immunol. 35, 661-670.

Ellington, W.R., 2001. Evolution and physiological roles of phosphagen systems. Ann. Rev. Physiol. 63, 289-325.

Fernández-Gimenez, A.V., 2013. Digestive physiology of three species of decapod crustaceans of Argentina. J. Shellfish Res. 32, 767-777.

Feyereisen, R., 2006. Evolution of insect P450. Biochem. Soc. Trans. 34, 1252-1255.

Fridovich, I., 1995. Superoxide radical and superoxide dismutases. Annu. Rev. Biochem. 64, 97-112.

Garcia-Orozco, K.D., Aispuro-Hernandez, E., Yepiz-Plascencia, G., Calderon-de la-Barca, A.M., Sotelo-Mundo, R.R., 2007. Molecular characterization of arginine kinase, an allergen from the shrimp *Litopenaeus vannamei*. Int. Arch. Allergy Immunol. 144, 23-28.

Garg, P., Burgersm P.M., 2005. DNA polymerases that propagate the eukaryotic DNA replication fork. Crit. Rev. Biochem. Mol. Biol. 40, 115-128.

Ghaffari, N., Sánchez-Flores, A., Doan, R., García-Orozco, K.D., Chen, P.L., Ochoa-Leyva, A., Lopez-Zavala, A.A., Carrasco, J.S., Hong, C., Brieba, L.G., Rudiño-Piñera, E., Blood, P.D., Sawyer, J.E., Johnson, C.D., Dindot, S.V., Sotelo-Mundo, R.R., Criscitiello, M.F., 2014. Novel transcriptome assembly and improved annotation of the whiteleg shrimp (*Litopenaeus vannamei*), a dominant crustacean in global seafood mariculture. Sci. Rep 4, 7081.

Grabherr, M.G., Haas, B.J., Yassour, M., Levin, J.Z., Thompson, D.A., Amit, I., Adiconis, X., Fan, L., Raychowdhury, R., Zeng, Q., Chen, Z., Mauceli, E., Hacohen, N., Gnirke, A., Rhind, N., di Palma, F., Birren, B.W., Nusbaum, C., Lindblad-Toh, K., Friedman, N., Regev, A., 2011. Full-length transcriptome assembly from RNA-Seq data without a reference genome. Nat. Biotechnol. 29, 644-652.

Hu, D.X., Pan, L.Q., Zhao, Q., Ren, Q., 2015. Transcriptomic response to low salinity stress in gills of the Pacific white shrimp, *Litopenaeus vannamei*. Mar. Genom. 24, 297-304.

Li, C., Sun, H., Chen, A., Ning, X., Wu, H., Qin, S,, Xue, Q., Zhao, J., 2010. Identification and characterization of an intracellular Cu, Zn-superoxide dismutase (icCu/Zn-SOD) gene from clam *Venerupis philippinarum*. Fish Shellfish Immunol. 28, 499-503.

Livingstone, D.R., 2003. Oxidative stress in aquatic organisms in relation to pollution and aquaculture. Rev. Med. Vet. 154, 427-430.

Livingstone, D.R., Lips, F., Martinez, P.G., Pipe, R.K., 1992. Antioxidant enzymes in digestive gland of the common mussel, *Mytilus edulis*. Mar. Biol. 112, 265-276.

Müller, M., Mentel, M., van Hellemond, J.J., Henze, K., Woehle, C., Gould, S.B., Yu, R.Y., van der Giezen, M., Tielens, A.G., Martin, W,F., 2012. Biochemistry and evolution of anaerobic energy metabolism in eukaryotes. Microbiol. Mol. Biol. Rev. 76, 444-495.

Rewitz, K., Styrishave, B., Andersen, O.. 2003. CYP330A1 and CYP4C39 enzymes in the shore crab *Carcinus maenas*: sequence and expression regulation by ecdysteroids and xenobiotics. Biochem. Biophys. Res. Commun. 310, 252-260.

Roncalli, V., Cieslak, M.C., Passamaneck, Y., Christie, A.E., Lenz, P.H., 2015. Glutathione S-Transferase (GST) gene diversity in the crustacean *Calanus finmarchicus*-Contributors to cellular detoxification. PLoS One. 10, e0123322.

Sanchez Garcia, J., Baranovskiy, A.G., Knatko, E.V., Gray, F.C., Tahirov, T.H., MacNeill, S.A., 2009. Functional mapping of the fission yeast DNA polymerase δ B-subunit Cdc1 by site-directed and random pentapeptide insertion mutagenesis. BMC Mol. Biol. 10, 82.

Snyder, M.J., 2007. Aquatic P450 species. In: Sigel, A., Sigel, H., Sigel, R.K.O., (Eds.,), The ubiquitous roles of cytochrome P450 proteins: metal ions pp. 97-126.

Strange, K., Denton, J., Nehrke, K., 2006. Ste20-type kinases: Evolutionarily conserved regulators of ion transport and cell volume. Physiology. 21, 61-68.

Suzuki, T., Kamidochi, M., Inoue, N., Kawamichi, H., Yazawa, Y., Furukohri, T., Ellington, W.R., 1999. Arginine kinase evolved twice: evidence that echinoderm arginine kinase originated from creatine kinase. Biochem. J. 340, 671-675.

Towle, D.W., Weihrauch, D., 2001. Osmoregulation by gills of euryhaline crabs: Molecular analysis of transporters. Am. Zool. 41, 770-780.

Tuena de Gomez-Puyou, M., Perez-Hernandez, G., Gomez-Puyou, A., 1999. Synthesis and hydrolysis of ATP and the phosphate-ATP exchange reaction in soluble mitochondrial F_1_ in the presence of dimethylsulfoxide. Eur. J. Biochem. 266, 691-696.

Van Wormhoudt, A., Sellos, D., 2003. Highly variable polymorphism of the alpha-amylase gene family in *Litopenaeus vannamei* (Crustacea Decapoda). J. Mol. Evol. 57, 659-671.

Wang, Y., Luo, P., Zhang, L., Hu, C., Ren, C., Xia, J., 2013. Cloning of sarco/endoplasmic reticulum Ca(^2+^)-ATPase (SERCA) gene from white shrimp, *Litopenaeus vannamei* and its expression level analysis under salinity stress. Mol. Biol. Rep. 40, 6213-6221.

Weihrauch, D., Ziegler, A., Siebers, D., Towle, D.W., 2001. Molecular characterization of V-type H+-ATPase (B-subunit) in gills of euryhaline crabs and its physiological role in osmoregulatory ion uptake. J. Exp. Biol. 204, 25-37.

Zhou, G., Somasundaram, T., Blanc, E., Parthasarathy, G., Ellington, W.R., Chapman, M.S., 1998. Transition state structure of arginine kinase: implications for catalysis of bimolecular reactions. Proc. Natl. Acad. Sci. U. S. A. 95, 8449-8454.

**Supplementary Figure Captions**

**Figure S1**. Counts for KEGG (A) and KOG (B) functions from annotated transcript products.

**Figure S2**. Counts for PFAM domains from annotated transcript products.

**Figure S3**. Enzyme Code (EC) distribution.

**Figure S4**. Enzyme code distribution of transferase.

**Figure S5**. Enzyme code distribution of oxidoreductases.

**Figure S6**. Enzyme code distribution of lyases.

**Figure S7**. Enzyme code distribution of ligases.

**Figure S8**. Enzyme code distribution of isomerases.

**Figure S9**. Enzyme code distribution of hydrolases.

**Figure S10**. Alignment of microsomal GST from *P. argentinus* and insects. The motif VERVRRXHLNDXENIX from microsomal GSTs is shaded (X is any residue).

**Figure S11**. Cellular component classification by GO terms.

**Supplementary tables**

**Table S1**.- Top 20 most abundant **A**) annotated and **B**) no annotation transcripts. Prot %ID = Amino acid identity percentage with most similar Swiss Prot; FPKM = Fragments per kb of transcript per million mapped reads.

A)

| Unigene ID | Isoform ID | Swissprot ID | Prot. ID% | Product name | FPKM | GenBank |
| --- | --- | --- | --- | --- | --- | --- |
| c8373_g1 | c8373_g1_i1 | SCP1_ASTLP | 85.86 | Sarcoplasmic_calcium-binding_protein_1 | 22887.19 | GEFN01022190.1 |
| c9552_g3 | c9552_g3_i1 | MYSA_DROME | 75 | Myosin_heavy_chain_muscle | 22523.57 | GEFN01023696.1 |
| c8909_g2 | c8909_g2_i1 | MLC1_DROSI | 58.39 | Myosin_light_chain_alkali | 19732.23 | GEFN01022858.1 |
| c10023_g6 | c10023_g6_i1 | ART2_YEAST | 63.64 | Putative_uncharacterized_protein_ART2 | 17542.53 | GEFN01000046.1 |
| c8909_g1 | c8909_g1_i1 | MLR_BOMMO | 60 | Myosin_regulatory_light_chain_2 | 15143.44 | GEFN01022857.1 |
| c10904_g3 | c10904_g3_i4 | ACT_PROCL | 86.67 | Actin | 13673.66 | GEFN01002258.1 |
| c3352_g1 | c3352_g1_i1 | TNNI_ASTLP | 91.67 | Troponin_I | 11900.54 | GEFN01002258.1 |
| c3116_g1 | c3116_g1_i1 | TNNCA_HOMAM | 82.67 | Troponin_C_isoform_2A | 11007.48 | GEFN01016977.1 |
| c7351_g1 | c7351_g1_i1 | KARG_STIJA | 43.91 | Arginine_kinase | 9996.807 | GEFN01020995.1 |
| c9552_g5 | c9552_g5_i1 | MYSA_DROME | 76.92 | Myosin_heavy_chain_muscle | 9242.349 | GEFN01023698.1 |
| c10904_g3 | c10904_g3_i1 | ACT2_XENTR | 85.94 | Actin_alpha_cardiac_muscle_2 | 8133.635 | GEFN01002255.1 |
| c10492_g1 | c10492_g1_i1 | TPM_PANBO | 98.94 | Tropomyosin | 5566.303 | GEFN01000821.1 |
| c10904_g3 | c10904_g3_i5 | ACT_PROCL | 87.7 | Actin | 5075.626 | GEFN01002259.1 |
| c10904_g3 | c10904_g3_i2 | ACT2_XENTR | 86.47 | Actin_alpha_cardiac_muscle_2 | 4977.216 | GEFN01002256.1 |
| c3915_g2 | c3915_g2_i1 | COX2_DROLO | 69.33 | Cytochrome_c_oxidase_subunit_2 | 4726.941 | GEFN01017688.1 |
| c17302_g1 | c17302_g1_i1 | COX1_ANOGA | 73.63 | Cytochrome_c_oxidase_subunit_1 | 4027.057 | GEFN01009456.1 |
| c19755_g1 | c19755_g1_i1 | ACT_MESVI | 88 | Actin | 3912.429 | GEFN01012077.1 |
| c3915_g1 | c3915_g1_i1 | COX3_AEDAE | 67.44 | Cytochrome_c_oxidase_subunit_3 | 3553.638 | GEFN01017687.1 |
| c9552_g9 | c9552_g9_i1 | MYSA_DROME | 65.22 | Myosin_heavy_chain_muscle | 2751.925 | GEFN01023702.1 |
| c21639_g1 | c21639_g1_i1 | CYB_DROYA | 73.84 | Cytochrome_b | 2681.274 | GEFN01014084.1 |

B)

| Unigene ID | Isoform ID | FPKM |
| --- | --- | --- |
| c10387_g1 | c10387_g1_i1 | 1.234474e+04 |
| c9552_g7 | c9552_g7_i1 | 1.058587e+04 |
| c6765_g1 | c6765_g1_i1 | 7.327312e+03 |
| c8449_g1 | c8449_g1_i1 | 5.590003e+03 |
| c4714_g2 | c4714_g2_i1 | 5.256098e+03 |
| c10098_g1 | c10098_g1_i1 | 4.891362e+03 |
| c17455_g1 | c17455_g1_i1 | 3.902346e+03 |
| c8372_g4 | c8372_g4_i1 | 3.078985e+03 |
| c4714_g1 | c4714_g1_i1 | 2.256161e+03 |
| c10098_g1 | c10098_g1_i2 | 1.352252e+03 |
| c9378_g1 | c9378_g1_i1 | 8.491003e+02 |
| c17609_g1 | c17609_g1_i1 | 7.398843e+02 |
| c10133_g1 | c10133_g1_i1 | 7.257838e+02 |
| c5326_g1 | c5326_g1_i1 | 6.978764e+02 |
| c5208_g1 | c5208_g1_i1 | 6.802441e+02 |
| c9459_g1 | c9459_g1_i1 | 6.044678e+02 |
| c10280_g1 | c10280_g1_i1 | 5.710552e+02 |
| c7436_g1 | c7436_g1_i1 | 5.257572e+02 |
| c6658_g1 | c6658_g1_i1 | 4.805796e+02 |
| c10805_g2 | c10805_g2_i3 | 4.263200e+02 |

**Table S2**. Mitochondrial transcripts. %IDE = Amino acid identity percentage; FPKM = Fragments per kilobase of transcript per million mapped reads.

| **gene\|prot_ID** | **Product** | **%IDE** | **Completeness** | | **FPKM** | **GenBank** |
| --- | --- | --- | --- | --- | --- | --- |
| c3915_g2_i1\|m.7377 | Cytochrome c oxidase subunit II | 82.17 | complete | 4.726941e+03 | | GEFN01017688.1 |
| c3915_g1_i1\|m.7375 | ATP synthase F0 subunit 8 | 62.75 | partial | 3.553638e+03 | | GEFN01017687.1 |
| c3915_g1_i1\|m.7372 | ATP synthase F0 subunit 6 | 80.80 | complete | 3.553638e+03 | | GEFN01017687.1 |
| c3915_g1_i1\|m.7371 | Cytochrome c oxidase subunit III | 81.68 | complete | 3.553638e+03 | | GEFN01017687.1 |
| c10976_g1_i1\|m.38964 | NADH dehydrogenase subunit 5 | 74.91 | partial | 7.936385e+02 | | GEFN01002608.1 |
| c21770_g1_i1\|m.62408 | NADH dehydrogenase subunit 4 | 75.85 | complete | 7.288552e+02 | | GEFN01014226.1 |
| c21770_g1_i1\|m.62409 | NADH dehydrogenase subunit 4L | 69.89 | partial | 7.288552e+02 | | GEFN01014226.1 |
| c21639_g1_i1\|m.61979 | NADH dehydrogenase subunit 6 | 64.78 | complete | 2.681274e+03 | | GEFN01014084.1 |
| c21639_g1_i1\|m.61978 | Cytochrome b | 89.68 | complete | 2.681274e+03 | | GEFN01014084.1 |
| c17316_g2_i1\|m.52592 | NADH dehydrogenase subunit 1 | 85.62 | complete | 1.227179e+03 | | GEFN01009470.1 |
| c17302_g1_i1\|m.52551 | NADH dehydrogenase subunit 2 | 65.62 | complete | 4.027057e+03 | | GEFN01009456.1 |
